# Supplementary material for: Sarcoeleganolides C–G, Five New Cembranes from the South China Sea Soft Coral Sarcophyton elegans
Source: Mar Drugs. 2022 Sep 10;20(9):574. doi: 10.3390/md20090574 (PMC9506240; doi:10.3390/md20090574)
Supplement: Supplementary file 1 [file marinedrugs-20-00574-s001.zip › marinedrugs-1902328-supplementary.pdf]

Supporting Information for Original article
Sarcoeleganolides C–G, Five New Cembranes from the South China Sea Soft Coral *Sarcophyton elegans*

Cili Wang [1,2], Jiarui Zhang [1,2], Xing Shi [1,2], Kai Li [1,2], Fengling Li [3], Xuli Tang [4], Guoqiang Li [1,2,*] and Pinglin Li [1,2,*]

[1] Key Laboratory of Marine Drugs, Chinese Ministry of Education, School of Medicine and Pharmacy, Ocean University of China, Qingdao 266003, China;
[2] Laboratory of Marine Drugs and Biological Products, National Laboratory for Marine Science and Technology, Qingdao 266235, China
[3] Biology Institute, Qilu University of Technology (Shandong Academy of Sciences), Jinan 250103, China;
[4] College of Chemistry and Chemical Engineering, Ocean University of China, Qingdao 266100, China;
* Correspondence: liguoqiang@ouc.edu.cn (G.L.); lipinglin@ouc.edu.cn (P.L.); Tel.: +86-532-8203-2323 (G.L.); +86-532-8203-3054 (P.L.)

Abstract: Five new cembranes, named sarcoeleganolides C–G (**1**–**5**), along with three known analogs (**6**–**8**) were isolated from soft coral *Sarcophyton elegans* collected from the Yagong Island, South China Sea. Their structures and absolute configurations were determined by extensive spectroscopic analysis, QM-NMR and TDDFT-ECD calculations. In addition, compound **3** exhibited better anti-inflammation activity compared with the indomethacin as positive control in zebrafish at 20 μM.

Keywords: *Sarcophyton elegans*; sarcoeleganolides C–G; cembranes; anti-inflammation activity

Table

1.NMR data of 1–5. 2
2.The Determination of Relative and Absolute Configurations for Compounds 1–5 4
2.1 Conformational search 4
2.2 Quantum chemical NMR calculation 4
2.3 Elucidation of absolute configurations by TDDFT-ECD 7
3.Anti-inflammation assay of 1–5 8
4.Computational Details 8
5.The 1D and 2D NMR spectra of 1–5 33

1. NMR data of 1–5.

Table S1. ^{1}H and ^{13}C NMR Data of sarcoeleganolide C (**1**).

Position	1[a]					
	type	δH[b] (J in Hz)	δC[c]	^{1}H-^{1}H COSY	HMBC	NOESY/1D-NOE
1	qC		160.7			
2	CH	4.94, m	79.1	H-3		H-18
3	CH	2.77, d, (4.2)	61.5	H-2	C-2	
4	qC		61.5			
5a	CH_2	1.37, m	38.8	H-6a,H-6b	C-4, C-6, C-7, C-18	
5b		2.08, m		H-6a,H-6b		
6a	CH_2	2.20, m	23.7	H-5a,H-5b,H-7		
6b		2.08, m		H-5a,H-5b,H-7		
7	CH	5.05, t, (7.2)	124.3	H-6a,H-6b	C-6,C-9,C-19	
8	qC		135.2			
9a	CH_2	2.11, m	38.8	H-10a,H-10b	C-8,C-11,C-19	
9b		2.18, m		H-10a,H-10b		
10a	CH_2	2.26, m	24.4	H-9a,H-9b,H-11	C-9,C-11,C-12	
10b		2.20, m		H-9a,H-9b,H-11		
11	CH	5.09, t, (6.6)	126.4	H-10a,H-10b	C-10,C-13,C-20	
12	qC		133.6			
13a	CH_2	2.04, m	36.6	H-14a,H-14b	C-1,C-11,C-12,C-20,C-14	
13b		2.45, m		H-14a,H-14b	C-1,C-11,C-12,C-14	
14a	CH_2	2.74, m	24.9	H-13a,H-13b	C-1,C-2,C-12,C-13,C-15	
14b		2.39, m		H-13a,H-13b	C-1,C-12,C-12,C-15	
15	qC		124.0			
16	qC		174.4			
17	CH_3	1.83, s	8.8		C-1,C-15,C-16	
18	CH_3	1.53, s	17.9		C-3,C-4,C-5	
19	CH_3	1.58, s	16.1		C-7,C-8,C-9	H-6a
20	CH_3	1.68, s	17.0		C-11,C-12,C-13	H-10a

[a] In chloroform -d4. [b]Recorded at 600 MHz. [c] Recorded at 150 MHz.

Table S2. ^{1}H and ^{13}C NMR Data of sarcoeleganolides D (**2**).

Position	2[a]					
	type	δH[b] (J in Hz)	δC[c]	^{1}H-^{1}H COSY	HMBC	NOESY
1	qC		158.3			
2	qC		108.3			H_2-5
3	CH	5.16, s	120.6		C-2,C-5,C-18	
4	qC		143.8			
5a	CH_2	2.20, m	40.2	H-6a,H-6b	C-4,C-3,C-18,C-6,C-7	
5b		2.20, m		H-6a,H-6b	C-4,C-3,C-18,C-6,C-7	
6a	CH_2	2.35, m	24.6	H-5a,H-5b,H-7	C-4,C-5,C-7,C-8	
6b		2.14, m		H-5a,H-5b,H-7	C-4,C-5,C-7,C-8	
7	CH	5.02, t, (6.6)	125.7	H-6a,H-6b	C-5,C-6,C-9,C-19	
8	qC		134.3			
9a	CH_2	2.29, m	37.0	H-10a,H-10b	C-7, C-8,C-11	
9b		2.03, m		H-10a,H-10b	C-7, C-10,C-19	
10a	CH_2	2.06, m	24.2	H-9a,H-9b,H-11	C-8,C-11,C-12	
10b		1.34, m		H-9a,H-9b,H-11	C-8	
11	CH	2.69, dd, (9.6, 3.3)	61.5	H-10a,H-10b	C-10	H-13a
12	qC		61.6			
13a	CH_2	1.68, m	34.0	H-14a,H-14b	C-11,C-12,C-20	
13b		1.89, m		H-14a,H-14b	C-11,C-12,C-14	
14a	CH_2	2.45, m	23.4	H-13a,H-13b	C-1,C-2,C-13,C-15	
14b		2.14, m		H-13a,H-13b	C-1	
15	qC		126.4			
16	qC		172.2			
17	CH_3	1.90, s	8.8		C-1,C-15,C-16	

Position	type	δH^b (*J* in Hz)	δC^c	^1H-^1H COSY	HMBC	NOESY
18	CH_3	1.57, s	15.9		C-3,C-4,C-5	
19	CH_3	1.66, s	15.0		C-7,C-8,C-9	H-6a
20	CH_3	1.29, s	16.6		C-11,C-12,C-13	H-13b
21	CH_3	3.14, s	50.2		C-2	H-13a

[a] In chloroform -d4. [b]Recorded at 600 MHz. [c] Recorded at 150 MHz.

Table S3. ^1H and ^{13}C NMR Data of sarcoeleganolide E (**3**).

Position	3[a]					
	**type**	**δH^b (*J* in Hz)**	**δC^c**	**^1H-^1H COSY**	**HMBC**	**NOESY**
1	qC		160.0			
2	CH	4.91, d, (10.0)	78.4	H-3		H-18
3	CH	4.74, d, (10.0)	125.3	H-2	C-2,C-5,C-18	H-5a
4	qC		139.0			
5a	CH_2	2.38, dd, (10.0, 3.0)	46.2	H-6	C-3,C-4,C-6,C-7,C-18	
5b		2.04, t, (11.5)		H-6	C-3,C-4,C-6,C-7,C-18	
6	CH	5.36, td, (10.0, 2.0)	71.1	H-5a,H-5b,H-7		H-3,H-9a
7	CH	5.19, d, (10.0)	127.3	H-6	C-5,C-6,C-9,C-19	
8	qC		141.9			
9a	CH_2	2.61, td, (14.0, 2.5)	29.1	H-10a,H-10b	C-7,C-8,C-10,C-11,C-19	
9b		1.76, m		H-10a,H-10b		
10a	CH_2	1.24, m	24.3	H-9,H-9b,H-11		
10b		1.86, m		H-9,H-9b,H-11	C-9,C-11,C-12	
11	CH	2.30, dd, (10.5, 2.5)	58.8	H-10a,H-10b	C-10	
12	qC		59.9			
13a	CH_2	1.09, m	35.2	H-14a,H-14b	C-14,C-20	H-2,H-11
13b		1.63, m		H-14a,H-14b	C-14	
14a	CH_2	1.74, m	22.0	H-13a,H-13b	C-1,C-13	
14b		1.59, m		H-13a,H-13b	C-2,C-13	
15	qC		124.0			
16	qC		173.9			
17	CH_3	1.61, s	8.8		C-1,C-15,C-16	
18	CH_3	1.34, s	18.3		C-3,C-4,C-5	H-5b
19	CH_3	1.46, s	22.4		C-7,C-8,C-9	H-7
20	CH_3	1.11, s	17.3		C-11,C-12,C-13	H-9a
21	qC		169.4			
22	qCH_3	1.65, s	20.9		C-21	

[a] In benzene -d6. [b]Recorded at 500 MHz. [c] Recorded at 125 MHz.

Table S4. ^1H and ^{13}C NMR Data of sarcoeleganolide F (**4**).

Position	4[a]					
	**type**	**δH^b (*J* in Hz)**	**δC^c**	**^1H-^1H COSY**	**HMBC**	**NOESY**
1	qC		151.9			
2	qC		148.2			
3	CH	5.23, s	117.0		C-1,C-2,C-4,C-5,C-18	H_2-14
4	qC		72.0			
5a	CH_2	2.16, m	48.5	H-6	C-3,C-4,C-6,C-7,C-18	
5b		2.03, m		H-6	C-3,C-6,C-7	
6	CH	4.30, t, (9.0)	73.4	H-5a,H-5b,H-7		H-5a
7	CH	4.88, d, (9.0)	124.8	H-6	C-9,C-19	
8	qC		141.1			
9a	CH_2	2.10, m	38.7	H-10a,H-10b		
9b		2.10, m		H-10a,H-10b		
10a	CH_2	2.22, m	24.3	H-9a,H-9b,H-11	C-11	
10b		2.10, m		H-9a,H-9b,H-11		
11	CH	4.86, d, (4.0)	125.9	H-10a,H-10b		H_2-13
12	qC		131.7			
13a	CH_2	2.33, m	36.2	H-14a,H-14b	C-1,C-11,C-12,C-14,C-20	
13b		2.33, m		H-14a,H-14b	C-1,C-11,C-12,C-14,C-20	
14a	CH_2	2.54, m	22.5	H-13a,H-13b	C-1,C-2,,C-12,C-13,C-15,	
14b		2.54, m		H-13a,H-13b	C-1,C-2,,C-12,C-13,C-15,	

15	qC		123.2			
16	qC		170.0			
17	CH_3	1.93, s	9.3		C-1,C-15,C-16	
18	CH_3	1.45, s	32.9		C-3,C-4,C-5	H-5a
19	CH_3	1.66, s	17.2		C-7,C-8,C-9	H-6
20	CH_3	1.60, s	17.1		C-11,C-12,C-13	
21	CH_3	3.21, s	55.1		C-6	

[a] In chloroform -d4. [b] Recorded at 500 MHz. [c] Recorded at 125 MHz.

Table S5. ^1H and ^{13}C NMR Data of sarcoeleganolide G (**5**).

Position	5[a]					
	type	δH^b (*J* in Hz)	δC^c	^1H-^1H COSY	HMBC	NOESY
1	qC		161.6			
2	CH	5.45, d, (10.5)	79.9	H-3	C-3	H-14a, H-18
3	CH	4.89, d, (10.5)	120.0	H-2	C-5,C-18	H-5a
4	qC		144.7			
5a	CH_2	2.20, m	39.5	H-6a,H-6b	C-6	
5b		2.32, m		H-6a,H-6b		
6a	CH_2	2.20, m	24.2	H-5a,H-5b,H-7		
6b		2.35, m		H-5a,H-5b,H-7	C-4,C-7,C-8	
7	CH	4.92, d, (5.0)	123.0	H-6a,H-6b	C-5,C-19	H_2-9
8	qC		135.5			
9a	CH_2	2.03, m	33.9	H-10a,H-10b	C-7,C-8,C-11	
9b		2.03, m		H-10a,H-10b	C-7,C-8,C-11	
10a	CH_2	1.71, m	34.4	H-9a,H-9b,H-11	C-8,C-9,C-11,C-12	
10b		1.71, m		H-9a,H-9b,H-11	C-8,C-9,C-11,C-12	
11	CH	3.98, t, (6.5)	72.1	H-10a,H-10b	C-10,C-12,C-13,C-20	H-14a
12	qC		151.9			
13a	CH_2	2.24, m	32.1	H-14a,H-14b	C-11,C-12,C-14,C-20	
13b		2.17, m		H-14a,H-14b	C-11,C-12,C-14,C-20	
14a	CH_2	2.26, m	27.0	H-13a,H-13b	C-1, C-2	
14b		2.46, m		H-13a,H-13b	C-2, C-13	
15	qC		124.2			
16	qC		174.9			
17	CH_3	1.87, s	9.0		C-1,C-15,C-16	
18	CH_3	1.78, s	15.9		C-3,C-4,C-5	
19	CH_3	1.64, s	17.1		C-7,C-8,C-9	
20a	CH_2	5.19, s	110.9		C-11,C-12,C-13	
20b		5.02, s				

[a] In chloroform -d4. [b] Recorded at 500 MHz. [c] Recorded at 125 MHz.

2. The Determination of Relative and Absolute Configurations for Compounds 1–5

2.1 Conformational search

Conformational search of all possible configurations were carried out by MacroModel integrated in Maestro V11.9 (Schrödinger Inc.)[30]. The OPLS3e force field[31] and an energy below a threshold of 10 kJ mol^{-1} were employed[32]. Eliminating redundant conformer used root-mean-squared-distance (RMSD) cutoff of 0.5 Å and the maximum iterations was 2500. After energy minimization, the unstable configurations were excluded.

2.2 Quantum chemical NMR calculation

In order to establish the relative configuration of molecules **1**, **4**. ^{13}C NMR chemical shifts were calculated by

Gaussian 16 program package[33]. Excluding unstable conformers by conformational search, the remaining conformers were optimized with the density functional theory (DFT) at the B3LYP/6-31G (d, p) level[34], and all minima displayed no imaginary frequencies by vibrational frequency analysis at the same level. The populations of conformers were calculated according to the Boltzmann distribution theory and their relative Gibbs free energy. GIAO calculations of NMR shielding were accomplished for all stable conformations by DFT GIAO model at PCM/mPW1PW91/6-31+G**[35] level for DP4+ calculations. The qccNMR results were shown in the following Table S6-S7 and Figures S1-S4.

Table S6. Experimental NMR data and calculated NMR data for **1.**

**Nuclei**	**SP^2**	Experiment **1**	Calculation	
			Conf.1（2*R*,3*S*,4*S*）	Conf.1（2*S*,3*S*,4*R*）
**C**	**X**	135.2	141.3	141.7
C		38.8	46.3	41.2
C		24.4	32.6	36.6
C	X	126.4	131.4	130.8
C		38.8	44.7	32.7
C		23.7	33.0	26.5
C	X	124.3	127.6	133.7
C		61.5	72.0	65.3
C		36.6	44.4	33.9
C		24.9	32.5	29.0
C	X	160.7	169.1	171.3
C		79.1	86.3	80.7
C		61.5	69.5	65.3
C	X	133.6	140.6	139.4
C		16.1	22.9	18.5
C	X	124.0	127.7	124.9
C	X	174.4	176.6	176.3
C		8.8	16.2	10.7
C		17.0	23.2	25.4
C		17.9	22.9	21.8
H		2.11	2.07	2.33
H		2.18	2.30	1.92
H		2.26	2.77	2.18
H		2.20	2.03	2.65
H	X	5.09	5.33	5.49
H		2.08	2.00	1.88
H		1.37	1.20	1.35
H		2.20	2.66	2.48
H		2.08	2.09	2.29
H	X	5.05	5.21	5.71
H		2.77	2.56	2.82
H		2.45	2.45	3.00
H		2.04	2.05	1.89
H		2.74	2.63	2.68
H		2.39	2.76	1.95
H		4.94	4.99	4.61
H		1.58	1.82	1.75
H		1.58	1.82	1.75
H		1.58	1.82	1.75
H		1.83	1.85	1.92
H		1.83	1.85	1.92
H		1.83	1.85	1.92
H		1.58	1.92	1.90
H		1.58	1.92	1.90
H		1.58	1.92	1.90
H		1.53	1.56	1.44
H		1.53	1.56	1.44
H		1.53	1.56	1.44

Functional	Solvent?	Basis Set	Type of Data
mPW1PW91	PCM	6-31+G(d,p)	Unscaled Shifts

	Isomer 1	Isomer 2	Isomer 3	Isomer 4	Isomer 5	Isomer 6
sDP4+ (H data)	99.97%	0.03%	-	-	-	-
sDP4+ (C data)	100.00%	0.00%	-	-	-	-
sDP4+ (all data)	100.00%	0.00%	-	-	-	-
uDP4+ (H data)	99.98%	0.02%	-	-	-	-
uDP4+ (C data)	0.00%	100.00%	-	-	-	-
uDP4+ (all data)	0.01%	99.99%	-	-	-	-
DP4+ (H data)	100.00%	0.00%	-	-	-	-
DP4+ (C data)	98.47%	1.53%	-	-	-	-
DP4+ (all data)	100.00%	0.00%	-	-	-	-

Figure S1. The DP4+ results between calculated and experimental NMR data for **1.**

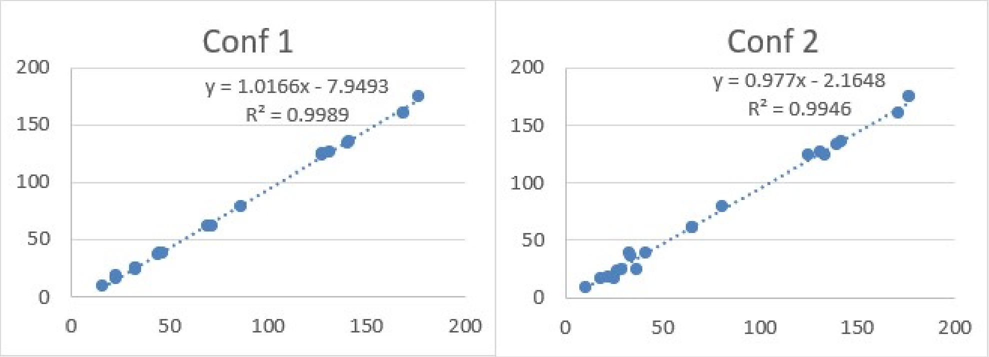

Figure S2. Correlation of experimental and calculated chemical shifts of compound **1.**

Table S7. Experimental NMR data and calculated NMR data for **4.**

Nuclei	SP²	Experiment **4**	Calculation	
			Conf.1（4*R*, 6*S*）	Conf.1（4*R*, 6*R*）
**C**	**X**	141.1	141.2	147.8
C		38.7	38.9	32.1
C		24.3	26.2	33.9
C	X	125.9	131.1	129.3
C		48.5	44.1	55.3
C		73.4	82.5	80.1
C	X	124.8	123.2	128.6
C	X	117	127.7	125.8
C		36.2	38.9	43.7
C		22.5	24.3	27.2
C	X	151.9	156.8	158.4
C	X	148.2	147.2	150.1
C		72	76.8	81.3
C	X	131.7	134.5	137.6
C	X	123.2	123.9	127.3
C	X	170	171.6	172.5
C		17.2	18.8	24.7
C		9.3	11	18.5
C		17.1	16.9	25.2
C		32.9	30.8	39.2
C		55.1	56.3	60.4
H		2.1	1.96	3.2
H		2.1	1.96	3.2
H		2.1	1.96	3.36
H		2.22	2.41	3.41

H	X	4.86	4.91	6.02
H		2.03	2.16	2.95
H		2.16	2.76	4.02
H		4.3	4.25	5.15
H	X	4.88	5.21	5.96
H	X	5.23	5.91	6.62
H		2.33	2.31	3.56
H		2.33	2.31	3.56
H		2.54	2.61	3.63
H		2.54	2.61	3.81
H		1.66	1.68	2.95
H		1.66	1.68	2.95
H		1.66	1.68	2.95
H		1.93	1.92	3.08
H		1.93	1.92	3.08
H		1.93	1.92	3.08
H		1.6	1.85	2.89
H		1.6	1.85	2.89
H		1.6	1.85	2.89
H		1.45	1.29	2.55
H		1.45	1.29	2.55
H		1.45	1.29	2.55
H		3.21	3.3	4.15
H		3.21	3.3	4.15
H		3.21	3.3	4.15

	A–B	C–D	E–F	G–H
1	Functional	Solvent?	Basis Set	Type of Data
2	mPW1PW91	PCM	6-31+G(d, p)	Unscaled Shifts
3				

	A–B	C	D	E	F	G	H	Is
4		Isomer 1	Isomer 2	Isomer 3	Isomer 4	Isomer 5	Isomer 6	Is
5	sDP4+ (H data)	54.47%	45.53%	–	–	–	–	
6	sDP4+ (C data)	0.24%	99.76%	–	–	–	–	
7	sDP4+ (all data)	0.29%	99.71%	–	–	–	–	
8	uDP4+ (H data)	100.00%	0.00%	–	–	–	–	
9	uDP4+ (C data)	100.00%	0.00%	–	–	–	–	
10	uDP4+ (all data)	100.00%	0.00%	–	–	–	–	
11	DP4+ (H data)	100.00%	0.00%	–	–	–	–	
12	DP4+ (C data)	100.00%	0.00%	–	–	–	–	
13	DP4+ (all data)	100.00%	0.00%	–	–	–	–	

Figure S3. The DP4+ results between calculated and experimental NMR data for **4.**

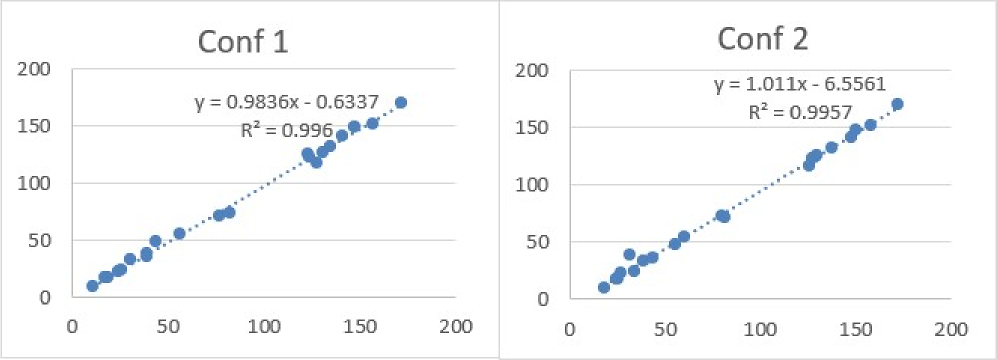

Figure S4. Correlation of experimental and calculated chemical shifts of compound **4.**

2.3 Elucidation of absolute configurations by TDDFT-ECD

To determine absolute configurations of compounds **1–5**, the spin-allowed excitation energies and rotatory

(Rn) and oscillator strengths (fn) of the lowest excited states of stable conformers were calculated for ECD spectra using TD-DFT method at the CAM-B3LYP/6-311G(d,p) level[36] with IEFPCM solvent model for methanol in agreement with the experiment condition. All the calculations in this article were performed using the Gaussian 09. Electronic transitions were expanded as Gaussian curves with a FQHM (full width at half maximum) for each peak of 0.40 eV. The ECD spectra were combined after Boltzmann weighting according to their population contribution.

3. Anti-inflammation assay of 1–5

Healthy macrophage fluorescent transgenic zebrafish (Tg: zlyz-EGFP) was provided by Biology Institute of Shandong Academy of Scienced (Jinan, China). Zebrafish maintenance and anti-inflammation assay were carried out as previously described[26]. Each zebrafish larva was photographed by a fluorescence microscope (AXIO, Zom.V16), and the number of macrophages around the nerve mound was calculated through Image-Pro Plus software[28]. One-way analysis of variance was calculated by GraphPad Prism 7.00 software[29]. Sarcoeleganolides C–G (**1**–**5**) were tested for anti-inflammatory activities with zebrafish models. Three dpf (days post fertilization) healthy macrophage fluorescent transgenic zebrafish were used as animal models to evaluated the anti-inflammatory effects of **1**–**5**.

Table S8. Effects of samples on the anti-inflammatory effects of zebrafish internodes.

Group	Concentration	Number of macrophages around nerve mound (mean ± SEM)	Inhibition rate
Control	—	2.333 ± 0.954	—
$CuSO_4$ model	20 μM	18.2 ± 1.685	—
Indomethacin	20 μM	10.36 ± 0.845	43.08%
**1**	20 μM	16.17 ± 1.364	11.15%
**2**	20 μM	15.45 ± 1.540	15.11%
**3**	20 μM	7.938 ± 1.302	56.38%
**4**	20 μM	11.57 ± 1.221	36.43%
**5**	20 μM	13.79 ± 1.538	24.23%

4. Computational Details

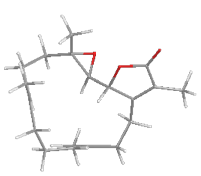
Conf 1
1a
33.85%

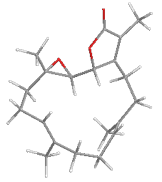
Conf 1
1b
31.73%

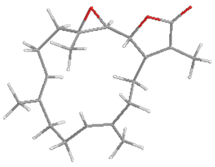
Conf 1
1c
12.96%

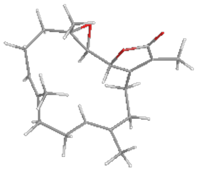
Conf 1
1d
10.56%

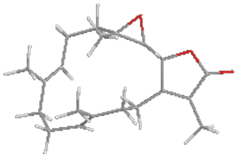
Conf 1
1e
6.89%

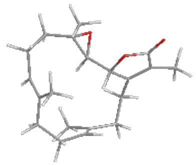
Conf 1
1f
2.04%

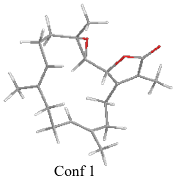
Conf 1
1g
1.04%

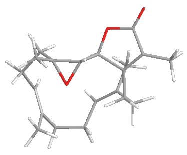
Conf 2
1a
52.16%

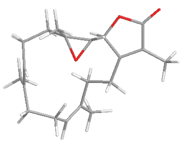
Conf 2
1b
11.14%

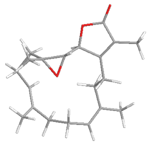
Conf 2
1c
10.78%

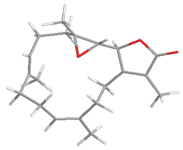
Conf 2
1d
7.86%

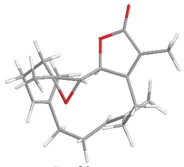
Conf 2
1e
4.52%

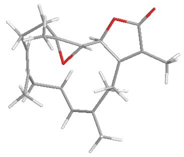
Conf 2
1f
4.42%

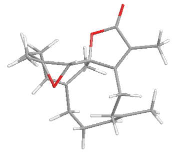
Conf 2
1g
3.05%

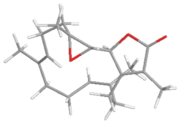
Conf 2
1h
2.98%

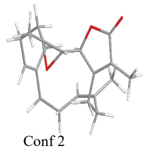
Conf 2
1i
2.01%

Figure S5. Stable conformers of compound **1** for relative configurations.

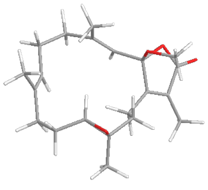
2a
69.25%

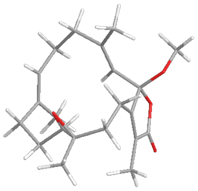
2b
23.28%

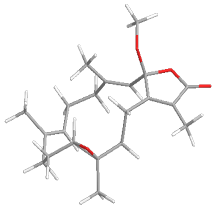
2c
3.94%

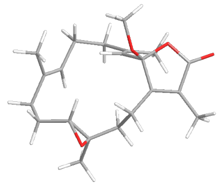
2d
3.43%

Figure S6. Stable conformers of compound **2** for relative configurations.

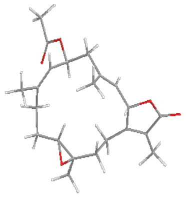
3a
69.76%

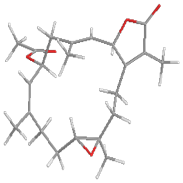
3b
17.63%

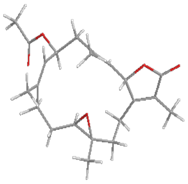
3c
5.91%

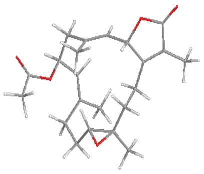
3d
4.56%

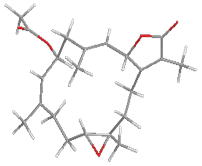
3e
1.28%

Figure S7. Stable conformers of compound **3** for relative configurations.

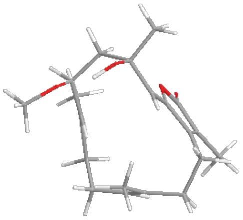

Conf 1
4a
66.20%

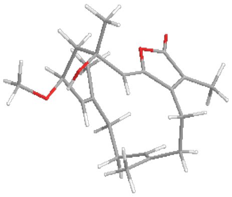

Conf 1
4b
22.77%

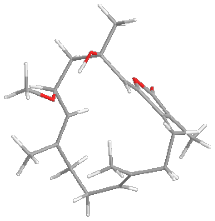

Conf 1
4c
6.05%

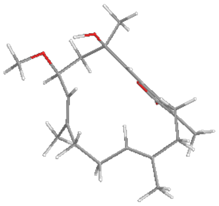

Conf 1
4d
1.71%

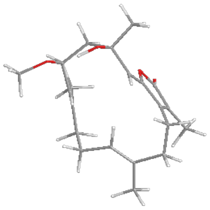

Conf 1
4e
1.09%

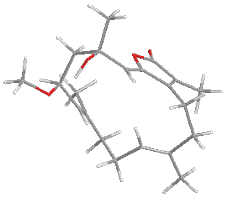

Conf 1
4f
1.06%

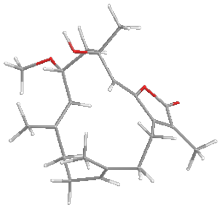

Conf 2
4a
44.54%

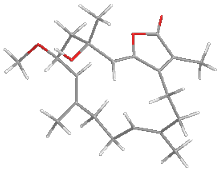

Conf 2
4b
17.74%

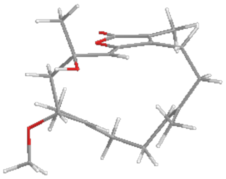

Conf 2
4c
17.05%

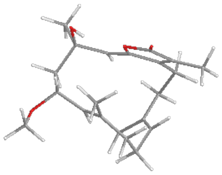

Conf 2
4d
15.68%

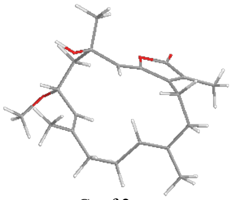

Conf 2
4e
1.94%

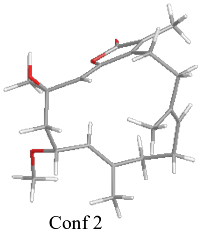

Conf 2
4f
1.30%

Figure S8. Stable conformers of compound **4** for relative configurations.

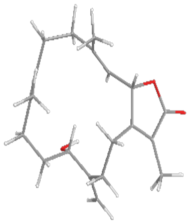

5a
45.54%

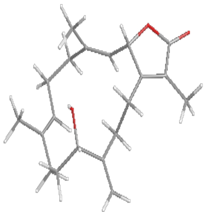

5b
24.53%

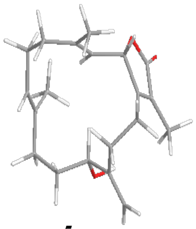

5c
14.14%

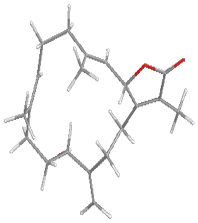

5d
10.51%

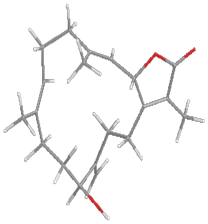

5e
2.33%

Figure S9. Stable conformers of compound **5** for relative configurations.

Table S9. Optimized Z-Matrixes of compounds **1-5** in the Gas Phase (Å) at B3LYP/6-31G (d, p) level.

		Con f. 1- **1**a			Con f. 1-**1** b	
C	-3.05318	-0.29069	-0.99297	-3.2968	-0.42333	-0.56977
C	-3.08345	1.05024	-1.70492	-3.34566	0.647	-1.6385
C	-2.66238	2.3083	-0.92647	-2.9826	2.0772	-1.17071
C	-1.17177	2.46515	-0.79455	-1.51257	2.31803	-0.9605
C	-1.67986	-1.9156	1.97224	-1.37256	-2.53457	1.28135
C	-2.96836	-1.68371	1.15482	-2.46222	-2.71004	0.20133
C	-2.93106	-0.41737	0.33781	-2.5666	-1.53658	-0.74378
C	0.24176	-0.68529	0.88963	0.31318	-0.78957	0.56076
C	1.05214	2.97986	0.17669	0.60131	2.99241	0.15953
C	1.98153	1.95018	0.87593	1.56301	2.08573	0.9785
C	2.19428	0.68642	0.10837	2.10101	0.93069	0.2018
C	1.05894	-0.22642	-0.29734	1.20762	-0.10225	-0.44697
C	-0.44658	-1.96555	1.09076	-0.01865	-2.21733	0.67588
C	-0.43873	2.73274	0.29855	-0.86493	2.61033	0.18023
C	-3.23765	-1.44813	-1.94433	-4.15356	-0.17661	0.64659
C	3.35344	0.14402	-0.31489	3.38511	0.60262	-0.04191
C	3.04278	-1.11688	-0.99983	3.3955	-0.62026	-0.85443
O	1.69142	-1.3105	-0.99911	2.11068	-1.00301	-1.11122
C	4.76806	0.60205	-0.18148	4.66648	1.24862	0.36958
C	-1.00594	2.88673	1.68667	-1.51653	2.664	1.53907
O	3.79522	-1.92175	-1.52297	4.34179	-1.25616	-1.28789
O	0.80471	-1.64914	1.79437	0.91823	-1.57363	1.60329
C	-0.37693	-3.12367	0.12441	0.57201	-3.30241	-0.19555
H	-2.46349	0.97645	-2.61105	-2.69826	0.36456	-2.47806
H	-4.10859	1.19606	-2.07925	-4.37333	0.6875	-2.03112
H	-3.16842	2.34721	0.04226	-3.56086	2.32683	-0.27653
H	-3.03671	3.17271	-1.49535	-3.32113	2.76792	-1.9563
H	-0.63798	2.39881	-1.74534	-0.92014	2.2981	-1.87774
H	-1.76795	-2.85572	2.53162	-1.29517	-3.45302	1.87781
H	-1.55554	-1.10906	2.70455	-1.65763	-1.72166	1.95842
H	-3.16325	-2.55847	0.52852	-3.41527	-2.88954	0.71133
H	-3.8026	-1.62433	1.86866	-2.24621	-3.61546	-0.37824
H	-2.79301	0.48643	0.92933	-1.96656	-1.60763	-1.65157
H	-0.20687	0.17234	1.38802	-0.44353	-0.09299	0.92003
H	1.27415	3.95516	0.63167	0.68542	4.00205	0.58481
H	1.33626	3.05142	-0.87904	0.96175	3.05754	-0.87303
H	1.57384	1.70307	1.86487	1.04618	1.71102	1.8713
H	2.95329	2.41964	1.05228	2.40317	2.68622	1.3373
H	0.37584	0.26925	-0.99564	0.56122	0.34364	-1.21129
H	-2.38878	-1.51102	-2.63933	-5.13878	0.21346	0.35793
H	-3.33563	-2.41427	-1.44536	-4.30678	-1.08039	1.24248
H	-4.13295	-1.29758	-2.56383	-3.70041	0.57489	1.30558
H	4.83823	1.56315	0.33345	5.25652	0.56957	0.9977
H	5.36009	-0.1328	0.37807	5.27859	1.48443	-0.50948
H	5.23461	0.70335	-1.16907	4.4957	2.17139	0.92932
H	-2.07577	2.67137	1.73649	-2.60489	2.59112	1.49148
H	-0.5002	2.22488	2.40186	-1.16105	1.84574	2.17982
H	-0.85031	3.9116	2.05219	-1.25973	3.59844	2.05591
H	-1.07842	-2.97474	-0.70278	0.39456	-4.28033	0.26703
H	0.62383	-3.23779	-0.28993	0.10128	-3.30783	-1.18459
H	-0.6567	-4.05033	0.63932	1.64435	-3.1653	-0.32875
		Con f. 1- **1**c			Con f. 1-**1** d	
C	3.65029	-0.4093	-0.40179	3.15188	0.57614	0.30692
C	3.94961	1.0469	-0.68821	3.18899	2.05991	0.0023

C	3.65877	1.99464	0.5065	1.95082	2.85818	0.44961
C	2.23001	1.85804	0.94975	0.69137	2.47215	-0.29409
C	0.85298	-2.85317	-1.02715	2.2303	-2.55914	-1.16743
C	2.35993	-2.53519	-0.986	3.4453	-1.82246	-0.56305
C	2.73045	-1.08098	-1.11585	3.35374	-0.32286	-0.67049
C	-1.31549	-1.67787	-0.26946	0.11651	-1.22956	-0.68107
C	-0.22336	2.0872	0.83835	-1.74455	2.6354	-0.93163
C	-0.89359	1.44673	-0.40741	-1.78461	1.22565	-1.5618
C	-2.13595	0.69149	-0.08574	-1.98068	0.14772	-0.54274
C	-2.07417	-0.66835	0.56139	-0.84773	-0.49064	0.23037
C	-0.01251	-2.2806	0.09866	0.96148	-2.38625	-0.35198
C	1.17322	2.54489	0.49011	-0.52266	3.01303	-0.11394
C	4.4499	-1.00724	0.72938	2.92051	0.21838	1.75331
C	-3.42365	1.00837	-0.31689	-3.14317	-0.39254	-0.12827
C	-4.25836	-0.11138	0.14479	-2.84307	-1.36251	0.93164
O	-3.45772	-1.09524	0.65253	-1.49667	-1.37945	1.15769
C	-4.03908	2.21913	-0.93468	-4.54993	-0.13285	-0.55347
C	1.26454	3.69727	-0.47727	-0.78914	4.10048	0.89574
O	-5.47123	-0.23345	0.1158	-3.59969	-2.06871	1.57647
O	-1.26658	-3.01425	0.25139	-0.26724	-2.53175	-1.14669
C	0.67696	-1.96618	1.40495	0.95312	-3.0762	0.9917
H	5.01135	1.15208	-0.95831	4.0629	2.49327	0.51193
H	3.3598	1.38295	-1.54928	3.34546	2.21005	-1.07329
H	3.89218	3.01825	0.19417	2.16549	3.92544	0.2993
H	4.33537	1.75618	1.3358	1.81204	2.73798	1.53195
H	2.03105	1.03242	1.63331	0.81531	1.69477	-1.04599
H	0.74876	-3.94431	-0.9743	2.45603	-3.63092	-1.23933
H	0.43604	-2.54819	-1.99418	2.04915	-2.19523	-2.18588
H	2.80669	-3.08828	-1.82643	3.5876	-2.14216	0.47366
H	2.79201	-2.96909	-0.08062	4.33611	-2.15461	-1.11341
H	2.21436	-0.54213	-1.91345	3.48198	0.06222	-1.68415
H	-1.56345	-1.63014	-1.33114	0.49455	-0.57262	-1.46371
H	-0.85034	2.92107	1.18112	-1.86036	3.36929	-1.74318
H	-0.18273	1.34903	1.6469	-2.6382	2.75263	-0.30487
H	-0.1705	0.75423	-0.85717	-0.87692	1.04536	-2.14632
H	-1.11106	2.21905	-1.15005	-2.62142	1.19832	-2.2667
H	-1.69165	-0.62196	1.58367	-0.28835	0.25287	0.8068
H	4.35773	-2.09315	0.79706	1.87628	0.40161	2.04145
H	5.51539	-0.76709	0.61377	3.14106	-0.82889	1.97181
H	4.14034	-0.58738	1.69624	3.53996	0.84107	2.41275
H	-4.64236	1.94395	-1.80844	-5.05519	-1.0718	-0.80944
H	-4.71222	2.71433	-0.22395	-5.12246	0.32746	0.26173
H	-3.2804	2.94037	-1.24911	-4.59312	0.5331	-1.41902
H	2.27943	4.09339	-0.56691	0.11889	4.46445	1.38279
H	0.93756	3.40074	-1.48317	-1.2861	4.9576	0.41998
H	0.60471	4.51733	-0.16371	-1.47376	3.74067	1.67709
H	1.24053	-2.84206	1.74596	1.3002	-4.10987	0.87657
H	1.38122	-1.13999	1.27668	1.63291	-2.57059	1.68517
H	-0.03856	-1.70453	2.18727	-0.04204	-3.08438	1.43282
		Con f. 1- **1** e			Con f. 1-**1** f	
C	-3.53531	0.40439	-0.7211	3.03359	-0.27299	0.59676
C	-3.87277	-0.88792	-1.43535	3.58022	1.13931	0.55853
C	-3.49137	-2.16267	-0.63945	2.77462	2.22646	1.30413
C	-2.0165	-2.1584	-0.36697	1.32117	2.44354	0.94168
C	-0.74825	2.93333	-0.67536	1.37937	-2.69443	-1.48715
C	-2.25629	2.62215	-0.72227	2.68644	-2.46791	-0.69839
C	-2.6391	1.2616	-1.24005	3.03654	-1.01365	-0.52376

C	1.42126	1.65492	-0.09174	-0.28792	-0.93092	-0.73698
C	0.1202	-1.93448	0.90648	-0.68217	3.00463	-0.41292
C	0.78344	-1.42065	-0.38668	-1.53663	1.96646	-1.19543
C	2.10402	-0.77349	-0.15102	-2.00674	0.84595	-0.32957
C	2.19286	0.54281	0.57965	-1.05957	-0.14731	0.30433
C	0.10211	2.16963	0.34539	0.14594	-2.33394	-0.68088
C	-1.37663	-2.15502	0.8112	0.78511	2.66606	-0.26947
C	-4.27585	0.62085	0.57431	2.59247	-0.77237	1.94772
C	3.33818	-1.14076	-0.5424	-3.2644	0.54615	0.05153
C	4.28306	-0.10123	-0.10522	-3.20206	-0.61192	0.95128
O	3.60188	0.88643	0.54923	-1.89938	-0.98468	1.1177
C	3.81327	-2.33817	-1.29525	-4.57554	1.1786	-0.2794
C	-2.04667	-2.31458	2.15234	1.5794	2.65377	-1.55553
O	5.49118	-0.04274	-0.25877	-4.10474	-1.20352	1.5196
O	1.34124	2.88544	0.64331	-0.99907	-1.91688	-1.50198
C	-0.61262	1.61789	1.55567	-0.17678	-3.27313	0.45951
H	-4.95321	-0.91911	-1.6397	4.57533	1.12666	1.03148
H	-3.36221	-0.91412	-2.40576	3.74491	1.43133	-0.4819
H	-3.76903	-3.03452	-1.24907	3.32063	3.1727	1.16761
H	-4.07909	-2.22081	0.28097	2.82309	2.01713	2.37844
H	-1.42082	-2.04871	-1.2721	0.64114	2.48874	1.79297
H	-0.64527	3.99537	-0.41901	1.30973	-3.7482	-1.78523
H	-0.3133	2.81246	-1.67455	1.3941	-2.09482	-2.40509
H	-2.69516	2.80973	0.26082	3.49847	-2.95022	-1.25953
H	-2.69447	3.37702	-1.39315	2.62946	-2.98589	0.26391
H	-2.16616	0.98481	-2.18502	3.3594	-0.52186	-1.4439
H	1.6874	1.77986	-1.14286	0.32706	-0.26049	-1.33756
H	0.62481	-2.85499	1.23401	-0.75902	3.95351	-0.96091
H	0.28987	-1.21097	1.71391	-1.13578	3.16748	0.57091
H	0.12483	-0.666	-0.83485	-0.95243	1.5744	-2.03709
H	0.88561	-2.23136	-1.11348	-2.40702	2.47145	-1.62312
H	1.91686	0.45307	1.63289	-0.33732	0.34744	0.96195
H	-3.9936	-0.13847	1.315	3.36516	-0.58353	2.70585
H	-4.09533	1.60133	1.02003	1.69164	-0.2411	2.28277
H	-5.35856	0.51537	0.41956	2.36668	-1.84115	1.95359
H	4.34311	-2.03667	-2.20706	-5.25023	0.44742	-0.74136
H	4.52141	-2.91808	-0.69039	-5.07045	1.54022	0.63046
H	2.98371	-2.99221	-1.57571	-4.45849	2.02122	-0.96517
H	-3.11183	-2.54544	2.07627	1.09603	3.27353	-2.31948
H	-1.56938	-3.12264	2.72436	2.59565	3.03345	-1.41158
H	-1.93519	-1.40206	2.75424	1.66559	1.64046	-1.96926
H	-1.18295	2.41419	2.04715	-0.03949	-4.30994	0.13119
H	-1.31428	0.8346	1.25498	0.49469	-3.09792	1.30671
H	0.08558	1.2042	2.2853	-1.20131	-3.14543	0.80485
		Con f. 1- **1** g				
C	-3.3341	0.00314	-0.26364			
C	-3.18039	1.27612	-1.06896			
C	-2.64171	2.50286	-0.29738			
C	-1.25623	2.3422	0.26946			
C	-1.752	-2.81214	0.90217			
C	-2.91314	-2.51539	-0.07428			
C	-2.82141	-1.15205	-0.71609			
C	0.12375	-1.1594	0.53749			
C	1.16726	2.9154	0.68632			
C	1.49592	1.6015	1.43137			
C	1.97806	0.52589	0.5112			
C	1.0517	-0.32805	-0.32364			

C	-0.39891	-2.50942	0.28612			
C	-0.15264	3.03347	-0.06077			
C	-4.11535	0.1247	1.01967			
C	3.24926	0.16526	0.24739			
C	3.22069	-0.89008	-0.77313			
O	1.92588	-1.13939	-1.12747			
C	4.54748	0.66429	0.78894			
C	-0.09933	4.08267	-1.14382			
O	4.14433	-1.49203	-1.29417			
O	0.66755	-2.27108	1.26727			
C	-0.01657	-3.36191	-0.90079			
H	-2.53706	1.08722	-1.93695			
H	-4.1703	1.55733	-1.46156			
H	-3.32962	2.72096	0.5318			
H	-2.69502	3.36588	-0.967			
H	-1.1819	1.60562	1.06769			
H	-1.78747	-3.86596	1.20737			
H	-1.87044	-2.20189	1.80453			
H	-2.91586	-3.276	-0.86448			
H	-3.85432	-2.63429	0.47357			
H	-2.25084	-1.11398	-1.64473			
H	-0.51311	-0.51209	1.13813			
H	1.2006	3.73159	1.42329			
H	1.98879	3.12516	-0.01045			
H	0.62862	1.25916	2.00556			
H	2.28481	1.81662	2.15798			
H	0.43791	0.28137	-0.99648			
H	-3.54681	0.68421	1.7747			
H	-5.04973	0.67968	0.85923			
H	-4.36671	-0.84682	1.45347			
H	4.4	1.40883	1.57511			
H	5.14852	1.12032	-0.00773			
H	5.1371	-0.16406	1.20014			
H	-1.06046	4.26033	-1.63048			
H	0.25694	5.03976	-0.73735			
H	0.62209	3.78897	-1.91893			
H	-0.24625	-4.4125	-0.68749			
H	-0.5893	-3.06761	-1.78725			
H	1.04367	-3.27219	-1.13249			
		Con f. 2- **1**a			Con f. 2-**1** b	
C	-3.36525	0.53547	-0.06095	-3.40905	-0.37567	0.26125
C	-3.43313	2.04316	0.07418	-3.8585	0.52834	-0.87138
C	-2.11236	2.76204	-0.28485	-2.70852	1.3973	-1.45335
C	-1.05501	2.59339	0.77507	-2.32761	2.60188	-0.63416
C	-1.47601	-2.20786	1.21157	-0.7654	-2.42029	1.03147
C	-2.94607	-1.73142	1.07353	-2.29901	-2.58817	0.91517
C	-3.08014	-0.2329	1.0015	-2.92251	-1.59309	-0.02819
C	0.43298	-1.81975	-0.56725	1.14976	-1.64941	-0.572
C	0.93885	2.61625	-0.7547	-0.26278	1.63548	0.42959
C	1.08563	1.23918	-1.46011	0.90526	1.61978	-0.58506
C	1.8534	0.25362	-0.6404	2.01791	0.71115	-0.16869
C	1.17161	-0.76735	0.23219	1.7646	-0.66251	0.40702
C	-0.80795	-2.48609	-0.12418	-0.02487	-2.50226	-0.28959
C	0.27538	2.55122	0.60717	-1.26036	2.74353	0.16707
C	-3.61153	0.01572	-1.4531	-3.49521	0.19943	1.65068
C	3.18649	0.13306	-0.48996	3.34653	0.93582	-0.17384
C	3.43958	-0.91893	0.50339	4.01491	-0.24598	0.38856

O	2.2437	-1.42468	0.9427	3.07315	-1.17172	0.74789
C	4.30457	0.89921	-1.11401	4.14128	2.11741	-0.62124
C	1.20388	2.46035	1.79374	-0.9796	4.04135	0.88044
O	4.49854	-1.33612	0.93971	5.20321	-0.45378	0.56373
O	-0.82861	-1.39773	-1.10143	-0.12637	-1.30911	-1.12966
C	-1.13119	-3.83132	-0.73429	-0.16137	-3.79603	-1.05773
H	-4.22635	2.42877	-0.5797	-4.25118	-0.08781	-1.68899
H	-3.70645	2.31794	1.10071	-4.67673	1.18326	-0.54244
H	-2.33637	3.83487	-0.39644	-3.03411	1.75577	-2.43982
H	-1.75599	2.4215	-1.26312	-1.84615	0.74653	-1.62737
H	-1.43517	2.55442	1.79743	-3.00403	3.45362	-0.72835
H	-1.44628	-3.14516	1.77902	-0.36373	-3.19823	1.69405
H	-0.91575	-1.47322	1.79697	-0.56934	-1.45592	1.50677
H	-3.49419	-2.0798	1.95849	-2.71568	-2.49438	1.92376
H	-3.41395	-2.21932	0.21257	-2.53038	-3.60608	0.57869
H	-2.91206	0.27417	1.95369	-2.90527	-1.88283	-1.07978
H	1.07567	-2.34794	-1.27396	1.87718	-2.00164	-1.30527
H	0.374	3.26621	-1.43099	0.14926	1.76459	1.43849
H	1.9382	3.05521	-0.65033	-0.75534	0.66179	0.40881
H	1.59855	1.39847	-2.4155	0.51549	1.2929	-1.55777
H	0.09057	0.84144	-1.67631	1.3004	2.631	-0.72624
H	0.50862	-0.29105	0.95852	1.19231	-0.59566	1.33478
H	-4.6227	0.28556	-1.78924	-3.11081	-0.47578	2.42002
H	-2.91483	0.47692	-2.16613	-2.93684	1.14108	1.71359
H	-3.50102	-1.06703	-1.53757	-4.53773	0.44198	1.90173
H	4.77194	1.57181	-0.38343	3.4965	2.94285	-0.93291
H	5.08663	0.21962	-1.47219	4.79555	2.47019	0.18508
H	3.95288	1.50187	-1.95581	4.78894	1.8503	-1.46579
H	1.87852	1.59761	1.72308	0.02951	4.40903	0.64581
H	1.84941	3.34848	1.85009	-1.69705	4.82301	0.60931
H	0.64991	2.38126	2.73516	-1.01319	3.90696	1.971
H	-0.73643	-4.63625	-0.10394	0.42038	-3.76393	-1.98397
H	-2.21559	-3.96811	-0.81726	0.19755	-4.6346	-0.44995
H	-0.69317	-3.92231	-1.73285	-1.20825	-3.98929	-1.31497
		Con f. 2- **1**c			Con f. 2- **1**d	
C	-3.44852	-0.19231	0.3036	-3.31428	-0.31178	0.4133
C	-3.42551	1.31895	0.3666	-3.89015	0.57106	-0.67601
C	-2.52592	1.90301	-0.76825	-2.83253	1.42179	-1.42772
C	-1.82079	3.1795	-0.39031	-2.30915	2.59392	-0.64709
C	-0.96967	-2.63932	1.07905	-0.77098	-2.50135	1.03906
C	-2.48512	-2.3729	1.25359	-2.31469	-2.58857	1.02785
C	-2.78893	-0.90102	1.23196	-2.96884	-1.5755	0.12207
C	0.72734	-1.62488	-0.66206	1.11226	-1.78097	-0.59619
C	0.18364	2.01117	0.56532	0.14904	2.01418	-0.62989
C	1.14717	1.5838	-0.57092	0.66087	1.2716	0.62995
C	2.06114	0.46881	-0.17592	1.8956	0.46127	0.39093
C	1.54622	-0.85136	0.35034	1.91536	-1.04465	0.47486
C	-0.46014	-2.44699	-0.33905	-0.1378	-2.52213	-0.33792
C	-0.61739	3.24669	0.20154	-1.04309	2.88807	-0.3092
C	-4.10573	-0.78418	-0.91602	-3.13772	0.32224	1.76808
C	3.40786	0.42668	-0.19571	3.14667	0.89702	0.1456
C	3.8342	-0.88782	0.3058	4.03197	-0.27128	0.06419
O	2.73109	-1.62545	0.64024	3.30167	-1.40836	0.28384
C	4.41801	1.44094	-0.61861	3.67728	2.28571	0.00439
C	0.03468	4.56373	0.53289	-0.72706	4.14933	0.45798
O	4.95918	-1.33544	0.44565	5.23375	-0.32329	-0.13477
O	-0.58516	-1.10428	-0.90314	-0.14294	-1.22878	-1.01794

C	-0.83708	-3.53449	-1.32003	-0.47654	-3.69145	-1.23191
H	-4.43726	1.73971	0.28391	-4.3934	-0.05876	-1.41907
H	-3.0232	1.63283	1.33561	-4.65338	1.24188	-0.25728
H	-3.13419	2.07094	-1.66458	-3.31974	1.80968	-2.33547
H	-1.78657	1.14388	-1.03713	-2.02727	0.76031	-1.76035
H	-2.34283	4.11273	-0.606	-3.0827	3.30115	-0.3388
H	-0.7501	-3.67669	1.35995	-0.36501	-3.34014	1.62032
H	-0.42351	-2.00315	1.78183	-0.48211	-1.58058	1.55393
H	-2.77758	-2.79483	2.22519	-2.65754	-2.47367	2.06219
H	-3.05141	-2.91892	0.49349	-2.61573	-3.59504	0.71354
H	-2.32839	-0.34321	2.05023	-3.13264	-1.90995	-0.90326
H	1.29319	-1.87309	-1.56204	1.74579	-2.11085	-1.41985
H	0.77516	2.21578	1.46654	-0.08814	1.27562	-1.39742
H	-0.47806	1.17384	0.80145	0.96157	2.63839	-1.02292
H	0.54599	1.26644	-1.43232	0.88654	2.00705	1.41156
H	1.74274	2.44296	-0.89421	-0.13775	0.62974	1.00871
H	0.99702	-0.71573	1.28513	1.63683	-1.37429	1.48075
H	-5.11843	-0.37871	-1.04554	-2.49399	1.20731	1.69777
H	-3.54586	-0.52375	-1.82472	-2.70001	-0.35814	2.50382
H	-4.1806	-1.87388	-0.87643	-4.10348	0.67278	2.15932
H	3.94454	2.36724	-0.95293	4.62631	2.38915	0.54263
H	5.09829	1.67724	0.20878	3.87433	2.52847	-1.04765
H	5.03433	1.05275	-1.43898	2.97069	3.02311	0.39516
H	0.18666	4.66798	1.6167	-0.24259	3.93087	1.41916
H	1.0305	4.63912	0.07339	-0.02753	4.78471	-0.10367
H	-0.56295	5.41436	0.18818	-1.62931	4.7347	0.66388
H	-0.47261	-3.29604	-2.32384	0.05567	-3.61876	-2.18528
H	-0.40349	-4.49163	-1.00886	-0.1915	-4.6317	-0.74559
H	-1.92496	-3.65494	-1.36756	-1.551	-3.7306	-1.43912
		Con f. 2- **1**e			Con f. 2- **1**f	
C	-3.17066	0.49384	0.85783	3.24784	-0.48381	-0.43042
C	-3.50554	1.82652	0.21661	3.76394	0.70324	-1.22438
C	-2.36428	2.39819	-0.65533	2.73546	1.83767	-1.45053
C	-1.13997	2.82382	0.10882	2.403	2.6435	-0.22645
C	-1.45872	-2.29735	0.60055	0.6271	-2.93313	-0.63528
C	-2.98046	-2.03634	0.61736	2.17412	-2.82296	-0.53795
C	-3.36033	-0.6437	0.17164	2.71734	-1.52964	-1.08447
C	0.392	-1.5098	-1.06279	-1.07478	-1.51803	0.8002
C	0.56317	2.01538	-1.54982	-0.10161	2.3951	-0.26528
C	1.93759	1.31372	-1.54798	-0.95908	1.66186	0.80379
C	2.14315	0.26898	-0.49692	-1.94687	0.74571	0.16416
C	1.03818	-0.63293	-0.00771	-1.53234	-0.61881	-0.32705
C	-0.82298	-2.2998	-0.77919	-0.06366	-2.58467	0.67011
C	0.14737	2.68634	-0.25354	1.19718	2.92426	0.29501
C	-2.57845	0.54728	2.24267	3.37902	-0.38462	1.06644
C	3.27936	-0.04322	0.15733	-3.25463	0.92481	-0.10117
C	2.99047	-1.15278	1.07494	-3.75164	-0.28396	-0.77468
O	1.6711	-1.49256	0.97099	-2.72647	-1.18322	-0.91074
C	4.65008	0.54108	0.07505	-4.15881	2.08036	0.17199
C	1.24871	3.29043	0.58267	1.0683	3.8166	1.50474
O	3.73457	-1.73397	1.84662	-4.87068	-0.53739	-1.18536
O	-0.88375	-1.05406	-1.54196	0.27187	-1.28058	1.24105
C	-1.08623	-3.52509	-1.62378	-0.08635	-3.69266	1.69898
H	-4.38617	1.71217	-0.42694	4.6496	1.12364	-0.72785
H	-3.76917	2.56195	0.98878	4.09044	0.3581	-2.21243
H	-2.76441	3.27768	-1.18528	1.84118	1.4086	-1.91224
H	-2.11635	1.66288	-1.42675	3.17144	2.52046	-2.19642

H	-1.34637	3.36996	1.03152	3.2732	3.08318	0.26629
H	-1.25794	-3.2816	1.04271	0.34244	-3.96255	-0.88056
H	-0.97635	-1.56276	1.247	0.26931	-2.30811	-1.45843
H	-3.32692	-2.21919	1.64262	2.60961	-3.64339	-1.12274
H	-3.48928	-2.77434	-0.01312	2.492	-2.984	0.49696
H	-3.77949	-0.56242	-0.83192	2.67237	-1.45971	-2.17401
H	1.06899	-1.8697	-1.83785	-1.80873	-1.60687	1.60324
H	-0.19911	1.29877	-1.86387	-0.70311	3.22137	-0.66981
H	0.60934	2.77578	-2.34317	0.09013	1.71341	-1.09631
H	2.74374	2.04994	-1.47341	-0.29692	1.07326	1.44688
H	2.05748	0.83318	-2.5298	-1.4751	2.38878	1.43697
H	0.26721	-0.04925	0.50372	-0.76723	-0.54998	-1.1055
H	-2.46686	-0.44118	2.69658	2.81375	0.47762	1.44293
H	-1.58863	1.02365	2.22852	3.02109	-1.27405	1.59016
H	-3.20535	1.15762	2.90697	4.42733	-0.21752	1.35035
H	5.0149	0.80554	1.07466	-5.02249	1.76476	0.76994
H	5.35824	-0.18525	-0.34358	-3.63766	2.87804	0.70781
H	4.67047	1.43613	-0.55173	-4.55177	2.49435	-0.76494
H	1.8435	4.00347	-0.00607	0.39959	4.66461	1.29831
H	0.84394	3.82254	1.4497	0.63465	3.28062	2.35946
H	1.94976	2.53043	0.94985	2.03923	4.21532	1.81647
H	-2.16298	-3.67558	-1.76144	-0.50427	-3.3384	2.64619
H	-0.62214	-3.42602	-2.6098	-0.69545	-4.52926	1.33821
H	-0.68006	-4.41705	-1.13347	0.92671	-4.06762	1.88495
		Con f. 2- **1**g			Con f. 2- **1**h	
C	-0.61689	2.37741	1.30638	3.76892	-0.45281	0.13416
C	0.02212	3.62123	0.72005	3.64979	-1.89148	-0.33486
C	0.87572	3.34011	-0.53956	2.25079	-2.38554	-0.72648
C	2.10347	2.5135	-0.28104	1.29647	-2.40806	0.44437
C	-3.45263	0.09994	0.2245	1.34662	2.31638	0.86544
C	-2.61299	0.77844	1.31778	2.749	1.88539	0.37871
C	-1.81795	1.97253	0.8625	2.78083	0.43667	-0.04178
C	-1.43155	-1.15367	-1.0446	-0.84654	1.90997	-0.54571
C	1.66962	0.66341	-1.93429	-0.80784	-2.5836	-0.90947
C	1.61801	-0.87689	-1.81728	-1.17709	-1.20446	-1.52737
C	1.03534	-1.39699	-0.54362	-1.96691	-0.34928	-0.59038
C	-0.31608	-0.94904	-0.04308	-1.32461	0.71654	0.25879
C	-2.81764	-0.655	-0.93469	0.39361	2.66282	-0.26992
C	2.47832	1.36152	-0.86153	-0.04026	-2.50888	0.39589
C	0.18684	1.66407	2.36422	5.09504	-0.13461	0.77721
C	1.53044	-2.31873	0.30514	-3.27822	-0.41243	-0.28889
C	0.53773	-2.54295	1.36531	-3.54507	0.54849	0.78938
O	-0.5526	-1.75102	1.13867	-2.37893	1.18496	1.12811
C	2.81625	-3.07691	0.30022	-4.35376	-1.29607	-0.82649
C	3.79995	0.72389	-0.50723	-0.86064	-2.57931	1.6606
O	0.58638	-3.29269	2.32544	-4.58961	0.79819	1.36577
O	-1.83146	0.03502	-1.75923	0.3111	1.68944	-1.36153
C	-3.84928	-1.39474	-1.76607	0.47517	4.08972	-0.76147
H	-0.75712	4.34024	0.44045	4.33747	-2.04241	-1.18099
H	0.65115	4.11017	1.47639	4.03532	-2.54398	0.46375
H	1.19496	4.31314	-0.94413	2.35958	-3.40377	-1.13007
H	0.23358	2.88494	-1.29946	1.85073	-1.78428	-1.55149
H	2.78234	2.94252	0.45929	1.76694	-2.36859	1.42808
H	-4.11325	0.85156	-0.2305	1.43723	3.21334	1.48837
H	-4.11936	-0.61972	0.71546	0.9378	1.53225	1.5099
H	-1.98736	0.04033	1.82369	3.46958	2.07462	1.18054
H	-3.33958	1.11517	2.07372	3.04859	2.5346	-0.45826

H	-2.30959	2.58811	0.10622	1.87669	0.1016	-0.53746
H	-1.29014	-2.02705	-1.68267	-1.66426	2.42869	-1.04946
H	0.64872	1.05038	-1.96695	-0.22849	-3.12665	-1.66438
H	2.11651	0.88218	-2.91533	-1.7358	-3.14597	-0.75169
H	2.61435	-1.30331	-1.96036	-1.7606	-1.3869	-2.43711
H	0.99888	-1.24527	-2.6475	-0.26205	-0.68297	-1.81824
H	-0.29079	0.09843	0.25954	-0.52405	0.30043	0.87571
H	0.29676	2.30329	3.25178	5.92146	-0.37515	0.09331
H	-0.26075	0.72072	2.68693	5.19678	0.91545	1.06227
H	1.20139	1.454	2.00398	5.25021	-0.74902	1.6754
H	2.62599	-4.15719	0.30001	-4.6451	-2.05103	-0.08506
H	3.42633	-2.83532	-0.57311	-5.25174	-0.71287	-1.0617
H	3.39881	-2.8548	1.2029	-4.02698	-1.81503	-1.73167
H	3.66608	-0.25504	-0.03033	-1.60592	-1.77555	1.71236
H	4.40967	0.55293	-1.40581	-1.42409	-3.52214	1.70772
H	4.37542	1.35405	0.1786	-0.23034	-2.51391	2.55399
H	-3.38868	-1.84681	-2.64949	0.1511	4.77901	0.02654
H	-4.31947	-2.1876	-1.17351	1.50738	4.34413	-1.02954
H	-4.63615	-0.70716	-2.0976	-0.15969	4.23874	-1.64015
		Con f.2- **1**i				
C	-3.11681	0.99207	0.44575			
C	-3.0753	2.28713	-0.34481			
C	-1.67663	2.6724	-0.87922			
C	-0.639	2.84132	0.20109			
C	-1.96183	-2.07359	0.81836			
C	-3.36969	-1.53701	0.47409			
C	-3.35938	-0.17325	-0.17515			
C	0.19543	-1.93913	-0.67334			
C	1.37205	2.37245	-1.24224			
C	1.36013	0.88017	-1.66761			
C	1.84846	-0.04857	-0.60344			
C	0.89985	-0.90899	0.18671			
C	-1.14922	-2.48177	-0.39626			
C	0.68853	2.69112	0.07594			
C	-2.8491	1.08585	1.92925			
C	3.10293	-0.26825	-0.16707			
C	3.03863	-1.24585	0.92796			
O	1.73316	-1.60977	1.13496			
C	4.39742	0.332	-0.6038			
C	1.60488	2.87402	1.25999			
O	3.938	-1.71428	1.60428			
O	-0.93569	-1.44699	-1.40844			
C	-1.49602	-3.82777	-0.99127			
H	-3.74914	2.21112	-1.20656			
H	-3.44966	3.10918	0.28057			
H	-1.78429	3.61218	-1.44455			
H	-1.35906	1.91476	-1.60323			
H	-1.02221	3.13512	1.17817			
H	-2.05889	-2.96236	1.454			
H	-1.43611	-1.32044	1.40978			
H	-3.94367	-1.50655	1.40894			
H	-3.88502	-2.2517	-0.17769			
H	-3.53311	-0.14964	-1.25099			
H	0.88509	-2.5539	-1.25441			
H	0.90831	2.93966	-2.05767			
H	2.41607	2.70016	-1.18347			
H	1.99443	0.7791	-2.55651			

H	0.34743	0.58993	-1.95601			
H	0.18208	-0.2953	0.73788			
H	-1.77774	1.19933	2.13922			
H	-3.19549	0.20178	2.47308			
H	-3.34637	1.9646	2.35919			
H	5.16446	-0.44277	-0.71736			
H	4.76821	1.04661	0.14216			
H	4.28873	0.85957	-1.55529			
H	2.19089	1.96462	1.44872			
H	2.33141	3.67885	1.07911			
H	1.0478	3.11384	2.17207			
H	-1.28514	-4.62526	-0.26976			
H	-0.91399	-4.01476	-1.89881			
H	-2.56076	-3.87551	-1.24644			
		Con f. 1- **2**a			Con f. 1- **2**b	
C	3.52882	-0.85763	-0.63024	1.51792	2.35553	1.27114
C	3.7758	0.55742	-1.11679	2.94886	1.96926	0.95568
C	3.69072	1.64057	-0.02183	3.14401	0.4526	0.7361
C	2.33708	1.65979	0.63936	2.19938	-0.06855	-0.31422
C	0.74401	-3.32498	-0.83973	-1.41678	3.18538	-0.76892
C	2.26539	-3.065	-0.90806	-0.6058	3.57381	0.48014
C	2.60882	-1.6346	-1.22575	0.82159	3.10463	0.40225
C	-1.04555	-1.74184	-0.28913	-1.6423	0.82772	0.0128
C	-0.17771	2.06551	0.55746	1.33298	-1.9722	-1.86991
C	-0.7488	1.33743	-0.67771	-0.0811	-1.41816	-1.67197
C	-1.99413	0.55566	-0.42045	-0.67342	-1.48939	-0.29929
C	-1.94648	-0.7456	0.38679	-1.9211	-0.65914	-0.00252
C	0.01229	-2.44133	0.15113	-1.73671	1.71778	-0.98662
C	1.22853	2.56437	0.28648	2.46942	-1.14804	-1.27804
C	4.40824	-1.30356	0.51203	0.964	1.80211	2.55965
C	-3.23885	0.75187	-0.88341	-0.33439	-2.18246	0.80403
C	-4.07815	-0.37809	-0.43722	-1.3276	-1.88208	1.85432
C	-3.81579	1.84049	-1.72421	0.79559	-3.11889	1.10409
O	-5.25863	-0.58181	-0.65674	-1.38005	-2.29913	2.99756
C	0.58017	-2.456	1.54663	-2.19756	1.41506	-2.38909
O	-3.32646	-1.25315	0.28567	-2.25851	-1.01392	1.37696
O	2.01332	2.78908	1.4934	2.48146	0.24857	-1.70433
C	1.36431	3.63125	-0.77423	3.81107	-1.84109	-1.34068
O	-1.63588	-0.57647	1.7342	-2.94213	-1.11078	-0.84306
C	-2.50681	0.27943	2.48654	-4.24222	-0.55789	-0.59098
H	4.78486	0.61171	-1.55159	3.27904	2.49572	0.05259
H	3.07007	0.79965	-1.92051	3.61078	2.28355	1.77506
H	3.91844	2.61551	-0.46207	2.94448	-0.09287	1.66754
H	4.45224	1.4583	0.74681	4.18628	0.26032	0.46661
H	2.04384	0.70536	1.08092	1.15813	0.11291	-0.06549
H	0.59712	-4.37668	-0.5534	-0.88523	3.55062	-1.65904
H	0.309	-3.20071	-1.83787	-2.37159	3.73258	-0.76231
H	2.73154	-3.38298	0.02793	-1.10632	3.22162	1.38697
H	2.66962	-3.72396	-1.69126	-0.61602	4.6724	0.53362
H	2.04426	-1.20196	-2.0544	1.33926	3.42188	-0.50574
H	-1.32044	-1.84676	-1.33738	-1.28093	1.15448	0.98073
H	-0.82893	2.90542	0.82924	1.40417	-2.99449	-1.48704
H	-0.14	1.38207	1.40902	1.51824	-2.0409	-2.9496
H	-0.93113	2.0557	-1.48176	-0.1044	-0.3738	-2.00416
H	0.01372	0.63319	-1.03728	-0.76704	-1.95027	-2.34589
H	4.32403	-2.37089	0.72694	0.00033	2.23963	2.83093
H	5.4622	-1.08857	0.28807	1.66165	1.98079	3.38885

H	4.17085	-0.75839	1.4349	0.82712	0.71342	2.49741
H	-4.26085	1.42842	-2.638	0.74422	-3.42295	2.15348
H	-4.61701	2.35933	-1.18363	0.74928	-4.02326	0.48827
H	-3.05795	2.57591	-2.00479	1.76773	-2.64667	0.93448
H	-0.10373	-2.04755	2.28636	-2.46211	0.36988	-2.54091
H	0.83876	-3.48411	1.83012	-3.06793	2.03585	-2.64232
H	1.51273	-1.87765	1.58302	-1.40751	1.68372	-3.10252
H	2.37551	4.04258	-0.80425	4.6292	-1.16977	-1.07118
H	1.12665	3.22828	-1.76501	3.9946	-2.212	-2.35644
H	0.66737	4.45325	-0.5705	3.82722	-2.70205	-0.66223
H	-2.58014	1.27754	2.04424	-4.87569	-0.90056	-1.41137
H	-3.50627	-0.15827	2.5701	-4.63886	-0.92903	0.35954
H	-2.05649	0.3539		-4.21928	0.53547	-0.57473
		Con f. 1- **2**c			Con f. 1- **2**d	
C	3.71786	-0.87293	-0.34229	3.20522	-1.35088	0.10078
C	4.25255	0.54217	-0.43286	4.18003	-0.21163	-0.12275
C	3.82152	1.45451	0.73608	3.91545	1.04011	0.74112
C	2.31775	1.50681	0.84906	2.46587	1.45307	0.67291
C	0.67912	-2.70215	-1.4829	0.23107	-2.69302	-1.7765
C	2.21388	-2.69437	-1.32774	1.49534	-2.95907	-0.94757
C	2.82441	-1.32254	-1.23972	2.45566	-1.79888	-0.91921
C	-1.25984	-1.42646	-0.67072	-0.71734	-1.24687	0.02392
C	0.04918	1.85822	-0.35163	0.36403	1.89219	-0.75398
C	-0.77632	1.32564	0.83632	-0.55743	1.94182	0.48718
C	-2.06034	0.74762	0.33895	-1.76073	1.07279	0.34441
C	-2.26631	-0.75703	0.23489	-1.71704	-0.39047	0.76489
C	-0.1197	-2.06569	-0.35726	-0.84518	-1.80621	-1.18859
C	1.45839	2.32532	-0.0189	1.83789	2.14633	-0.46008
C	4.26878	-1.68555	0.80216	3.17006	-1.91681	1.49729
C	-3.14198	1.3803	-0.14393	-2.9943	1.37169	-0.09398
C	-4.10024	0.36266	-0.60756	-3.82706	0.16218	0.02577
C	-3.43979	2.8375	-0.26487	-3.55922	2.65139	-0.61234
O	-5.19868	0.51601	-1.11019	-5.0025	0.00853	-0.25156
C	0.4126	-2.28951	1.03265	-2.0569	-1.65268	-2.0814
O	-3.57413	-0.88111	-0.41574	-3.07518	-0.86357	0.5189
O	1.70251	2.71943	1.36367	2.1362	2.86425	0.7741
C	2.03508	3.25913	-1.05861	2.63521	2.5871	-1.66479
O	-2.37434	-1.25479	1.53847	-1.47878	-0.39725	2.15018
C	-2.80822	-2.61902	1.6451	-1.49969	-1.68763	2.7759
H	5.35132	0.51475	-0.45506	5.20134	-0.55961	0.09027
H	3.92304	0.99812	-1.37341	4.1551	0.07807	-1.17896
H	4.22377	2.45982	0.57942	4.56069	1.85713	0.40401
H	4.24042	1.09115	1.68224	4.17461	0.84264	1.78841
H	1.87193	0.60302	1.26273	1.8077	0.82404	1.2728
H	0.37061	-3.75615	-1.55534	-0.24901	-3.65476	-2.01216
H	0.40108	-2.23421	-2.4347	0.5196	-2.2774	-2.75399
H	2.61903	-3.20815	-2.21257	2.00483	-3.81422	-1.41703
H	2.49644	-3.30852	-0.46865	1.21995	-3.28805	0.05947
H	2.5099	-0.63205	-2.02522	2.58065	-1.30435	-1.88508
H	-1.52136	-1.31734	-1.72129	0.21446	-1.36186	0.56755
H	0.13334	1.07923	-1.11887	0.30139	0.91011	-1.23439
H	-0.50166	2.68747	-0.81131	0.0047	2.62189	-1.48897
H	-0.22327	0.56096	1.38704	-0.01712	1.61705	1.37986
H	-0.98076	2.14752	1.53	-0.86844	2.97527	0.66453
H	5.36539	-1.72917	0.74517	2.51759	-2.78849	1.58803
H	4.0285	-1.22077	1.76724	2.82612	-1.16576	2.22124
H	3.89396	-2.71081	0.82507	4.17786	-2.21625	1.81696

H	-2.70328	3.4398	0.27335	-4.44313	2.94712	-0.03463
H	-3.43883	3.15033	-1.31675	-2.82401	3.45899	-0.565
H	-4.43561	3.06026	0.1359	-3.88028	2.53768	-1.65526
H	0.50633	-3.36525	1.2353	-2.39259	-0.61283	-2.14458
H	1.41926	-1.8671	1.11836	-1.82463	-1.98817	-3.09727
H	-0.21357	-1.84325	1.80242	-2.90138	-2.25054	-1.72061
H	3.06986	3.53459	-0.84552	3.698	2.70066	-1.43956
H	1.9963	2.78969	-2.04845	2.52638	1.85629	-2.47461
H	1.43665	4.17712	-1.10266	2.2558	3.54879	-2.03101
H	-2.63501	-2.90819	2.68354	-1.33075	-1.50292	3.83882
H	-3.87531	-2.69931	1.41429	-2.4711	-2.17391	2.63915
H	-2.24164	-3.27765	0.98067	-0.70795	-2.33947	2.39111
		Con f. 1- **3**a			Con f. 1- **3**b	
C	0.65305	2.69825	0.36502	1.11103	2.69271	0.12074
C	1.07309	-2.26644	0.96253	1.06169	-2.17238	1.20584
C	2.09426	-1.42625	0.17833	2.05536	-1.40533	0.31139
C	-1.2804	-1.62182	0.68893	-1.24858	-1.39723	0.9388
C	-1.83345	2.14994	0.11683	-1.41872	2.31646	-0.03574
C	-2.0675	0.9758	-0.85675	-1.78811	1.07267	-0.86952
C	-2.97199	-0.07491	-0.31038	-2.79244	0.19352	-0.20483
C	-2.56736	-0.8884	0.89917	-2.45494	-0.52609	1.08302
C	-0.15159	-1.49209	1.40322	-0.10258	-1.30295	1.6309
C	-0.63374	2.97631	-0.30164	-0.16414	2.98588	-0.56249
C	-4.1611	-0.51246	-0.76291	-4.03071	-0.15681	-0.59756
C	-4.59733	-1.61881	0.1036	-4.56549	-1.11244	0.38555
O	-3.6608	-1.83911	1.06963	-3.63813	-1.33345	1.35979
C	-4.99507	-0.07224	-1.91895	-4.83731	0.24688	-1.78588
O	-0.13213	3.86087	0.73904	0.41874	3.94543	0.36319
O	-5.61608	-2.28799	0.04122	-5.65197	-1.66868	0.40393
C	0.00063	-0.64175	2.63393	0.1439	-0.36986	2.78354
C	-0.70507	3.59607	-1.6773	-0.21239	3.44319	-2.00147
O	3.04956	-2.43425	-0.32656	2.90482	-2.39674	-0.375
C	3.73413	-2.15016	-1.44597	2.41715	-2.93745	-1.50534
O	3.57607	-1.13327	-2.10274	1.32341	-2.65724	-1.97231
C	4.72335	-3.23441	-1.76863	3.38726	-3.91347	-2.10743
C	2.86042	-0.44746	1.01353	2.98575	-0.50894	1.06583
C	3.14217	0.83938	0.75053	3.43765	0.69508	0.67788
C	2.73488	1.58528	-0.50232	3.10631	1.35323	-0.64363
C	2.0144	2.92358	-0.24265	2.47745	2.75595	-0.51558
C	3.95737	1.63424	1.7402	4.36538	1.47206	1.5765
H	0.63468	1.92296	1.13278	1.05025	2.01449	0.97345
H	1.57866	-2.69997	1.83418	1.59851	-2.56342	2.07811
H	0.76864	-3.09005	0.31048	0.68459	-3.02252	0.63271
H	1.61344	-0.96336	-0.68305	1.5007	-0.87287	-0.46028
H	-1.28581	-2.28369	-0.17614	-1.31426	-2.1175	0.12427
H	-2.72863	2.78211	0.15023	-2.25196	3.02897	-0.0546
H	-1.65991	1.77553	1.12989	-1.25131	2.03797	1.00888
H	-1.09873	0.50381	-1.06771	-0.87848	0.47809	-1.02744
H	-2.45887	1.34649	-1.80668	-2.15102	1.3742	-1.85465
H	-2.54844	-0.27075	1.80022	-2.35792	0.1784	1.91282
H	-5.98767	0.24441	-1.5792	-4.30534	0.97101	-2.40612
H	-4.53215	0.75905	-2.45449	-5.0817	-0.62587	-2.40216
H	-5.14637	-0.89828	-2.62313	-5.78782	0.69242	-1.47124
H	-0.93121	-0.17365	2.95486	-0.73385	0.21371	3.06543
H	0.7427	0.14695	2.47483	0.95882	0.32422	2.5533
H	0.37582	-1.25654	3.46121	0.46689	-0.94494	3.66014
H	-1.66497	4.10802	-1.80705	-1.14047	3.99447	-2.1885

H	0.09134	4.32717	-1.82535	0.62559	4.10155	-2.23617
H	-0.62332	2.83261	-2.45744	-0.18427	2.58967	-2.68629
H	5.1081	-3.09564	-2.77915	2.9693	-4.33542	-3.02134
H	5.55294	-3.18081	-1.05495	4.3325	-3.40774	-2.32718
H	4.26107	-4.21957	-1.66808	3.59853	-4.71268	-1.39041
H	3.25185	-0.87821	1.93492	3.33204	-0.9073	2.01856
H	3.65108	1.80282	-1.06697	4.04454	1.45878	-1.20414
H	2.11388	0.96212	-1.14887	2.45344	0.71918	-1.24902
H	2.60704	3.54763	0.43515	3.11938	3.4006	0.09518
H	1.93452	3.47358	-1.18353	2.42584	3.21435	-1.50611
H	4.37529	0.99446	2.52191	4.69244	0.87328	2.43063
H	4.78233	2.15467	1.23802	3.87658	2.37288	1.96636
H	3.35076	2.40632	2.22855	5.25221	1.80953	1.02602
		Con f. 1- **3**c			Con f. 1- **3**d	
C	0.22298	2.10886	-0.57766	-1.44928	1.93969	-0.87441
C	1.34419	-2.16432	0.66446	-1.01859	-2.5648	0.9214
C	2.37781	-1.17764	0.09491	-2.05733	-1.46606	1.23423
C	-0.98752	-1.45599	0.28488	1.25253	-1.81654	0.31522
C	-2.34501	2.21694	-1.02634	1.04246	1.57414	-1.34271
C	-2.39555	0.76669	-1.56713	1.76959	1.2387	-0.02358
C	-3.00261	-0.23355	-0.63649	2.76158	0.131	-0.13029
C	-2.28551	-0.76332	0.5799	2.33941	-1.26064	-0.54808
C	0.06392	-1.49618	1.11767	0.02283	-2.16026	-0.09847
C	-1.09954	2.68454	-0.28381	-0.11139	2.52673	-1.08855
C	-4.21828	-0.80835	-0.71935	4.07377	0.12384	0.16981
C	-4.36564	-1.73547	0.41252	4.57462	-1.24744	-0.01149
O	-3.23584	-1.71315	1.16818	3.55501	-2.05598	-0.41771
C	-5.32136	-0.64208	-1.71021	4.98365	1.21176	0.63356
O	-0.48314	1.74349	0.63856	-1.11344	2.56547	-2.14221
O	-5.31494	-2.4479	0.70328	5.70304	-1.6825	0.1549
C	0.05976	-0.90657	2.50405	-0.41324	-2.15652	-1.5431
C	-1.23492	4.0915	0.2553	0.2657	3.88864	-0.5549
O	3.52504	-2.03998	-0.25108	-2.73529	-1.08441	0.00458
C	4.29941	-1.68472	-1.28752	-3.75637	-1.84721	-0.42147
O	4.08776	-0.71532	-1.99929	-4.16412	-2.82784	0.17982
C	5.46031	-2.62571	-1.4468	-4.30591	-1.33544	-1.72097
C	2.82896	-0.14623	1.08125	-1.43852	-0.25757	1.88744
C	2.94483	1.17919	0.89723	-1.80507	1.03491	1.8873
C	2.74617	1.89867	-0.41736	-2.97309	1.62213	1.12539
C	1.53257	2.8478	-0.4706	-2.54665	2.55514	-0.03617
C	3.32829	2.06279	2.05683	-1.01168	2.03312	2.69397
H	0.25891	1.29292	-1.29925	-1.51149	0.85592	-0.95775
H	1.8089	-2.69549	1.50325	-1.56361	-3.44673	0.57037
H	1.12864	-2.89804	-0.11801	-0.53967	-2.8275	1.86981
H	1.99876	-0.73442	-0.82475	-2.81248	-1.90372	1.8963
H	-0.9222	-1.95059	-0.68212	1.49557	-1.8599	1.37535
H	-2.45918	2.89764	-1.87783	1.74915	2.02081	-2.05231
H	-3.21605	2.39235	-0.38381	0.64716	0.66315	-1.80086
H	-1.39591	0.42628	-1.8539	1.01275	0.93502	0.71115
H	-2.98774	0.77591	-2.48516	2.26212	2.13019	0.37052
H	-2.14827	0.03388	1.31262	2.06779	-1.279	-1.60648
H	-5.04933	0.05129	-2.50831	4.46903	2.17328	0.68507
H	-5.5832	-1.6059	-2.16114	5.38876	0.98178	1.6257
H	-6.2253	-0.26345	-1.21924	5.83812	1.312	-0.04517
H	-0.94442	-0.67115	2.86003	0.42683	-2.24953	-2.23512
H	0.66117	0.00673	2.54647	-0.95385	-1.23937	-1.79643
H	0.50752	-1.61607	3.20883	-1.0982	-2.99009	-1.7304

H	-0.36493	4.38604	0.84374	-0.59832	4.554	-0.51338
H	-1.35802	4.80529	-0.56621	0.69858	3.82068	0.44731
H	-2.12114	4.16179	0.89549	1.01466	4.34534	-1.21169
H	5.97295	-2.42549	-2.3878	-3.55162	-1.45815	-2.50516
H	6.15664	-2.47824	-0.61425	-5.20388	-1.89252	-1.98806
H	5.11811	-3.66335	-1.41349	-4.5315	-0.26815	-1.6438
H	3.05911	-0.54876	2.06704	-0.58017	-0.52534	2.50085
H	3.64809	2.49948	-0.59402	-3.61032	0.83128	0.73548
H	2.68117	1.19083	-1.24563	-3.58032	2.21282	1.82288
H	1.52842	3.52393	0.38722	-3.42046	2.73869	-0.67258
H	1.62746	3.47244	-1.36815	-2.23199	3.52581	0.3548
H	3.56469	1.48002	2.95112	-0.5995	2.82331	2.05649
H	4.1947	2.68754	1.80631	-1.65674	2.53202	3.429
H	2.50985	2.74831	2.30929	-0.18216	1.56198	3.22819
		Con f. 1- **3**e				
C	0.53122	2.80726	0.34812			
C	1.25055	-2.13328	0.52738			
C	2.06487	-1.04704	-0.2038			
C	-1.16985	-1.70072	0.50753			
C	-1.92064	2.06722	0.23129			
C	-2.17348	0.93777	-0.78832			
C	-2.99884	-0.17898	-0.24501			
C	-2.47182	-1.05974	0.86745			
C	-0.0021	-1.55541	1.15291			
C	-0.8148	2.99837	-0.2283			
C	-4.20759	-0.62716	-0.63053			
C	-4.53262	-1.81174	0.18006			
O	-3.51028	-2.06821	1.04487			
C	-5.15144	-0.13574	-1.67633			
O	-0.32542	3.8799	0.82017			
O	-5.52979	-2.51468	0.14829			
C	0.18162	-0.82158	2.45288			
C	-1.02247	3.66239	-1.56892			
O	2.9002	-1.66418	-1.25964			
C	3.92803	-2.45573	-0.91866			
O	4.22816	-2.7375	0.23095			
C	4.66313	-2.94027	-2.13801			
C	2.87614	-0.16291	0.69582			
C	3.1328	1.14254	0.50911			
C	2.6694	1.94928	-0.68403			
C	1.83165	3.19094	-0.31459			
C	3.94997	1.8967	1.52691			
H	0.62627	2.00188	1.07852			
H	1.87532	-2.61355	1.2854			
H	0.9754	-2.88752	-0.21558			
H	1.37302	-0.46037	-0.80837			
H	-1.19481	-2.26741	-0.42224			
H	-2.84445	2.63959	0.37731			
H	-1.64029	1.64722	1.20188			
H	-1.20405	0.51885	-1.08916			
H	-2.64315	1.34191	-1.68791			
H	-2.41082	-0.50645	1.80775			
H	-4.76668	0.75322	-2.18006			
H	-5.33327	-0.91121	-2.42917			
H	-6.12189	0.1116	-1.23135			
H	-0.75669	-0.48598	2.89735			
H	0.83017	0.05065	2.32101			

H	0.68669	-1.47475	3.17488			
H	-2.03003	4.08912	-1.62353			
H	-0.30381	4.46802	-1.72768			
H	-0.91876	2.94007	-2.3848			
H	5.45352	-3.63042	-1.84307			
H	3.96846	-3.43501	-2.82299			
H	5.09663	-2.08508	-2.66639			
H	3.29481	-0.64948	1.57227			
H	3.56184	2.29651	-1.22107			
H	2.10785	1.32911	-1.38737			
H	2.397	3.83086	0.37219			
H	1.65144	3.78145	-1.21616			
H	4.3801	1.22428	2.27383			
H	4.76572	2.44892	1.04396			
H	3.33927	2.63834	2.05566			
		Con f. 1- **4**a			Con f. 1- **4**b	
C	-0.64298	1.55189	1.65773	-0.46941	1.72034	1.57209
C	0.50746	2.53557	1.58565	0.74631	2.62018	1.47372
C	0.90999	3.09512	0.20734	1.20411	3.08928	0.07942
C	1.83297	2.17717	-0.54101	2.05053	2.06614	-0.62181
C	-2.47592	-1.19713	0.66032	-2.50383	-0.87945	0.70368
C	-2.6256	0.33241	0.63612	-2.55865	0.65819	0.60317
C	-1.36225	1.16222	0.59544	-1.24285	1.39468	0.52717
C	-0.53317	-1.48962	-1.02143	-0.6019	-1.40406	-0.96579
C	2.76496	0.65714	-2.23421	2.86221	0.40586	-2.24485
C	2.38166	-0.83648	-2.05249	2.35884	-1.04493	-2.01438
C	1.94645	-1.13023	-0.65107	1.89997	-1.25477	-0.60571
C	0.56696	-1.39768	-0.26415	0.50344	-1.38759	-0.21061
C	-1.93234	-1.8932	-0.60561	-2.03381	-1.65831	-0.54267
C	1.69374	1.6095	-1.74824	1.8697	1.46034	-1.80483
C	2.69985	-1.12358	0.48119	2.65422	-1.27744	0.52582
C	1.80453	-1.36525	1.60997	1.74495	-1.40752	1.66136
O	0.52527	-1.56332	1.11932	0.45141	-1.50476	1.17721
C	-0.91558	1.06617	3.05996	-0.74472	1.25559	2.98112
C	4.15742	-0.87509	0.67301	4.12857	-1.14903	0.708
C	0.51043	1.7921	-2.66099	0.71069	1.70078	-2.73481
O	2.02207	-1.40395	2.80926	1.96186	-1.43357	2.86107
O	-2.81928	-1.65769	-1.71394	-2.89199	-1.35212	-1.65768
C	-1.93799	-3.41471	-0.38538	-2.19287	-3.16595	-0.29447
O	-3.45676	0.65242	-0.49921	-3.30762	1.09295	-0.56032
C	-3.95634	1.98393	-0.49901	-4.72207	0.99479	-0.40831
H	0.23481	3.37778	2.2397	0.5284	3.50605	2.08979
H	1.39122	2.08504	2.06317	1.59087	2.12758	1.97938
H	1.44218	4.04014	0.389	1.81429	3.99156	0.23081
H	0.0185	3.3533	-0.37231	0.34388	3.40107	-0.52048
H	2.74777	1.94615	0.00857	2.94104	1.78425	-0.05616
H	-3.47259	-1.61894	0.83668	-3.51165	-1.24342	0.93522
H	-1.85302	-1.46084	1.51752	-1.87237	-1.13637	1.55655
H	-3.19538	0.59321	1.5396	-3.1085	1.00151	1.48981
H	-1.07885	1.49119	-0.39923	-0.97851	1.72807	-0.47121
H	-0.40219	-1.35718	-2.09007	-0.45905	-1.32218	-2.03794
H	2.97507	0.81869	-3.29944	3.09177	0.51131	-3.31314
H	3.69488	0.84288	-1.68747	3.80055	0.53531	-1.69638
H	3.25422	-1.45187	-2.30118	3.18055	-1.73565	-2.23708
H	1.58931	-1.10747	-2.75493	1.5502	-1.27658	-2.71236
H	-1.86245	0.52873	3.15632	-1.7306	0.79933	3.10243
H	-0.11628	0.38357	3.38106	0.00342	0.51141	3.28704

H	-0.92148	1.90212	3.7719	-0.666	2.0909	3.6896
H	4.70666	-0.98671	-0.26577	4.66392	-1.36779	-0.22007
H	4.33595	0.14007	1.05176	4.39867	-0.13233	1.02312
H	4.57533	-1.57178	1.40869	4.48302	-1.83329	1.48712
H	-0.01623	0.84152	-2.81774	0.11424	0.78809	-2.86265
H	0.83815	2.13442	-3.65233	1.07132	1.98038	-3.73441
H	-0.21423	2.51549	-2.27916	0.04069	2.49058	-2.38656
H	-3.08314	-0.71676	-1.65434	-2.99732	-0.37762	-1.64503
H	-1.61367	-3.92835	-1.29671	-1.90594	-3.72881	-1.1892
H	-1.26206	-3.68915	0.42974	-1.56081	-3.48751	0.53857
H	-2.95193	-3.7488	-0.13821	-3.2388	-3.39273	-0.0592
H	-3.15025	2.72477	-0.5724	-5.05776	1.55937	0.47185
H	-4.61096	2.07679	-1.36939	-5.16437	1.43117	-1.30792
H	-4.53518	2.18077	0.41461	-5.05498	-0.04627	-0.31749
		Con f. 1- **4**c			Con f. 1- **4**d	
C	1.04104	2.07877	-1.31877	-1.31544	2.13247	0.82408
C	-0.25297	2.7174	-1.76084	-0.50318	3.22716	0.16674
C	-0.98903	3.46785	-0.61726	1.01845	3.14772	0.41186
C	-1.84107	2.55492	0.22058	1.64081	1.98234	-0.31409
C	2.32036	-1.50928	-0.87421	-2.53275	-1.21579	0.51428
C	2.44509	0.02646	-0.86748	-3.0096	0.23954	0.58624
C	1.16859	0.74562	-1.23698	-2.05042	1.28574	0.08505
C	0.46472	-1.58701	0.91221	-0.55561	-1.23824	-1.09905
C	-2.57177	1.08137	2.05763	3.4216	0.6829	-1.5201
C	-2.22944	-0.42856	2.09398	2.44107	-0.47055	-1.7825
C	-1.99376	-1.00992	0.73451	1.93072	-1.18163	-0.56386
C	-0.71033	-1.52676	0.27554	0.52243	-1.50318	-0.34729
C	1.77439	-2.16664	0.41792	-1.98157	-1.6948	-0.85123
C	-1.53714	1.95291	1.38096	2.90306	1.88242	-0.75586
C	-2.88057	-1.14016	-0.28839	2.63569	-1.68239	0.48758
C	-2.17361	-1.74267	-1.41702	1.68467	-2.31696	1.39764
O	-0.8587	-1.97408	-1.03687	0.41472	-2.19788	0.85942
C	2.16062	3.03435	-0.97852	-1.23479	2.08466	2.32842
C	-4.32436	-0.77582	-0.37374	4.0991	-1.66858	0.78683
C	-0.20963	2.08123	2.08303	3.9501	2.94772	-0.54608
O	-2.55909	-2.04456	-2.53167	1.85531	-2.88835	2.46025
O	2.7437	-2.00814	1.47097	-2.77924	-1.19932	-1.9442
C	1.63263	-3.67921	0.21087	-2.03345	-3.23168	-0.91498
O	2.92289	0.50819	0.41885	-4.23284	0.2771	-0.19256
C	4.34434	0.61537	0.49484	-4.94356	1.50756	-0.10288
H	-0.02717	3.43937	-2.55814	-0.69193	3.23214	-0.91367
H	-0.92655	1.96424	-2.18676	-0.85993	4.1905	0.55958
H	-1.63991	4.22697	-1.06894	1.20492	3.07773	1.49338
H	-0.25647	4.009	-0.00839	1.46979	4.09329	0.09151
H	-2.82847	2.3537	-0.19869	0.95949	1.15496	-0.49138
H	3.30895	-1.9528	-1.03391	-3.39242	-1.84284	0.7796
H	1.69923	-1.79672	-1.72867	-1.76872	-1.37058	1.2787
H	3.21135	0.29346	-1.60973	-3.28144	0.44159	1.62961
H	0.32468	0.12485	-1.52154	-2.01319	1.37258	-1.00055
H	0.4827	-1.21846	1.93176	-0.39285	-0.69731	-2.02467
H	-2.70214	1.40463	3.09989	3.78414	1.03124	-2.49863
H	-3.53776	1.20995	1.55945	4.31545	0.30114	-1.01135
H	-3.07037	-0.95488	2.56221	2.95539	-1.21049	-2.41049
H	-1.35887	-0.59958	2.73228	1.5985	-0.10191	-2.37435
H	3.13935	2.63148	-1.25961	-2.02911	1.48569	2.78135
H	2.02625	3.98999	-1.49779	-0.27891	1.64952	2.64947
H	2.19951	3.24935	0.09626	-1.28007	3.09547	2.7533

H	-4.46046	0.18114	-0.89443	4.40332	-0.72297	1.25347
H	-4.87768	-1.53315	-0.94029	4.34551	-2.47414	1.48588
H	-4.77171	-0.68564	0.6199	4.69669	-1.80113	-0.12013
H	0.41156	2.87537	1.66086	3.56443	3.83417	-0.03693
H	0.36496	1.14945	2.01459	4.38332	3.26545	-1.50486
H	-0.35331	2.28824	3.15219	4.78279	2.55446	0.05408
H	2.87865	-1.04143	1.5271	-3.47669	-0.62152	-1.57101
H	1.32424	-4.15783	1.14626	-1.64644	-3.58012	-1.87851
H	0.88703	-3.89831	-0.55774	-1.43721	-3.68258	-0.11642
H	2.5955	-4.10192	-0.09688	-3.07242	-3.5639	-0.81468
H	4.8348	-0.35797	0.3645	-5.87035	1.37677	-0.66737
H	4.72683	1.3137	-0.26042	-5.18471	1.73792	0.94433
H	4.57634	1.00115	1.4913	-4.37482	2.34384	-0.52732
		Con f. 1- **4**e			Con f. 1- **4**f	
C	-0.61678	1.62526	1.42466	-0.47652	1.71302	1.42202
C	0.66693	2.41902	1.28122	0.85995	2.42037	1.3056
C	1.10897	2.85094	-0.13197	1.34866	2.84212	-0.09518
C	1.6151	1.68961	-0.94027	1.7658	1.65882	-0.92214
C	-2.84382	-0.83005	0.43285	-2.82268	-0.56764	0.4849
C	-2.80252	0.70422	0.49683	-2.71115	0.96965	0.46282
C	-1.44547	1.36207	0.40379	-1.31892	1.5483	0.39278
C	-0.86956	-1.29925	-1.16063	-0.91425	-1.19989	-1.1285
C	3.26096	0.08178	-1.91895	3.28187	-0.03617	-1.96066
C	2.18699	-1.02205	-1.94154	2.13976	-1.07006	-1.96522
C	1.60741	-1.35764	-0.59668	1.56836	-1.37832	-0.61036
C	0.1745	-1.47717	-0.3412	0.13599	-1.42073	-0.32767
C	-2.34149	-1.50946	-0.85952	-2.38732	-1.3259	-0.78839
C	2.88807	1.37973	-1.22832	3.0088	1.27746	-1.25224
C	2.25512	-1.58583	0.57888	2.22488	-1.64152	0.55297
C	1.24182	-1.82481	1.60352	1.21989	-1.82041	1.59775
O	-0.00476	-1.78343	1.00757	-0.03368	-1.7104	1.02569
C	-0.87979	1.16837	2.83811	-0.77606	1.2331	2.82064
C	3.70923	-1.57425	0.91814	3.68359	-1.70587	0.86559
C	4.08057	2.23662	-0.8856	4.26002	2.06127	-0.94612
O	1.34864	-2.02799	2.80132	1.33813	-2.02498	2.79421
O	-3.10508	-1.05037	-1.98896	-3.1534	-0.8624	-1.91535
C	-2.59789	-3.02262	-0.76909	-2.72423	-2.81679	-0.639
O	-3.65086	1.18254	-0.56892	-3.41469	1.53995	-0.66969
C	-3.96997	2.56592	-0.48046	-4.83341	1.55572	-0.52794
H	0.55237	3.32043	1.90366	0.80454	3.31711	1.94269
H	1.48674	1.85232	1.7465	1.63161	1.7876	1.76829
H	1.87972	3.61907	-0.01969	2.1783	3.54236	0.03824
H	0.26387	3.33244	-0.64312	0.55075	3.40114	-0.60331
H	0.84348	0.99142	-1.24638	0.94646	1.00843	-1.2094
H	-3.89055	-1.13066	0.56089	-3.87072	-0.83329	0.66607
H	-2.28344	-1.21751	1.28569	-2.25227	-0.93349	1.34101
H	-3.28097	0.98401	1.44586	-3.21089	1.32919	1.3725
H	-1.17676	1.67313	-0.60232	-1.03894	1.89298	-0.59874
H	-0.64859	-1.05471	-2.19421	-0.7032	-0.97675	-2.169
H	3.55782	0.28639	-2.95816	3.56512	0.15818	-3.00562
H	4.16583	-0.30796	-1.43813	4.17232	-0.48552	-1.50568
H	2.64034	-1.92922	-2.36258	2.52501	-2.0008	-2.40236
H	1.38084	-0.74247	-2.62545	1.33917	-0.73324	-2.62948
H	-1.89141	0.78091	2.98467	-1.80871	0.89891	2.95098
H	-0.17871	0.36848	3.1143	-0.12134	0.39164	3.0856
H	-0.71882	1.98795	3.55097	-0.57894	2.02699	3.55352
H	4.06937	-0.55079	1.08413	4.10082	-0.70165	1.01398

H	3.88454	-2.13833	1.83963	3.84517	-2.26979	1.78965
H	4.31301	-2.01545	0.11964	4.24787	-2.18668	0.06108
H	4.72141	1.7335	-0.14813	4.89982	1.51349	-0.2403
H	3.80957	3.21525	-0.48223	4.05977	3.04785	-0.52163
H	4.70403	2.40022	-1.7758	4.85667	2.20206	-1.85836
H	-3.28642	-0.1006	-1.83646	-3.18568	0.11366	-1.83163
H	-2.29747	-3.51116	-1.70218	-2.46389	-3.35565	-1.55636
H	-2.02831	-3.46336	0.05432	-2.16869	-3.2566	0.19419
H	-3.66553	-3.20695	-0.6057	-3.79794	-2.93589	-0.45623
H	-3.07899	3.20037	-0.56916	-5.12549	2.07136	0.39666
H	-4.65246	2.78687	-1.30516	-5.22659	2.10491	-1.38771
H	-4.46839	2.78774	0.47387	-5.25914	0.54508	-0.52751
		Con f.2- **4**a			Con f.2- **4**b	
C	1.54176	1.8584	-0.62837	-1.21099	2.1753	0.32818
C	0.61355	2.67917	-1.49798	-0.02021	2.80241	1.02249
C	-0.49197	3.46206	-0.74922	1.1554	3.17816	0.09304
C	-1.65589	2.60516	-0.33989	1.78405	1.96937	-0.54206
C	2.24675	-1.7511	0.14204	-2.76404	-1.21812	0.036
C	2.74269	-0.35664	-0.24023	-2.92786	0.28539	0.26214
C	1.70703	0.54427	-0.85388	-1.67967	0.98149	0.72772
C	0.01144	-1.18178	1.31788	-0.54277	-1.22233	-1.27568
C	-3.25246	1.36187	1.07762	3.59601	0.39667	-1.27399
C	-2.97907	-0.12132	1.42517	2.56215	-0.69391	-1.59247
C	-2.28961	-0.85372	0.31748	1.8418	-1.26189	-0.40375
C	-0.90461	-1.30768	0.35024	0.39946	-1.48884	-0.36284
C	1.37154	-1.82807	1.40725	-1.99838	-1.62276	-1.2391
C	-2.00496	2.19439	0.88897	3.08815	1.66463	-0.61749
C	-2.81922	-1.22617	-0.8786	2.36291	-1.72074	0.76729
C	-1.77932	-1.92823	-1.62671	1.25769	-2.2342	1.57482
O	-0.63435	-1.97209	-0.84602	0.08351	-2.08839	0.856
C	2.29267	2.63927	0.42201	-1.80927	2.99432	-0.78798
C	-4.18962	-1.02755	-1.43235	3.77063	-1.7801	1.26339
C	-1.22785	2.49491	2.14486	4.201	2.53666	-0.09161
O	-1.7964	-2.43843	-2.73246	1.24591	-2.73558	2.6849
O	2.02021	-1.13963	2.49698	-2.57533	-0.96461	-2.38622
C	1.16399	-3.30566	1.79302	-2.09283	-3.14763	-1.43156
O	3.78791	-0.6051	-1.21512	-3.9453	0.38613	1.28913
C	4.63814	0.51061	-1.42846	-4.49065	1.69	1.4157
H	0.15143	2.04004	-2.26002	0.34528	2.13134	1.80886
H	1.2333	3.41366	-2.03412	-0.36152	3.724	1.51756
H	-0.86412	4.23778	-1.43248	1.88511	3.74708	0.67649
H	-0.05714	3.98825	0.10595	0.78625	3.86188	-0.68521
H	-2.29324	2.29149	-1.169	1.06621	1.27265	-0.96945
H	3.1374	-2.36154	0.33602	-3.77219	-1.64236	-0.04972
H	1.72217	-2.20745	-0.70099	-2.29442	-1.67762	0.90974
H	3.19897	0.10443	0.64167	-3.31974	0.7381	-0.65499
H	1.118	0.07716	-1.64365	-1.16519	0.47326	1.54416
H	-0.29845	-0.65433	2.21372	-0.21826	-0.75106	-2.19713
H	-3.85352	1.7818	1.89564	4.09886	0.66357	-2.21555
H	-3.86776	1.40141	0.17267	4.38763	-0.02398	-0.64328
H	-3.94156	-0.61027	1.61818	3.08716	-1.51975	-2.09141
H	-2.39815	-0.19038	2.34868	1.83691	-0.31657	-2.31848
H	1.64103	2.88989	1.26795	-1.14766	3.00357	-1.6642
H	3.15324	2.10002	0.82204	-2.78322	2.62616	-1.11558
H	2.65127	3.59046	0.00802	-1.92843	4.03986	-0.47481
H	-4.91336	-0.82226	-0.63849	4.46984	-2.02916	0.4596
H	-4.2133	-0.18544	-2.13651	4.08483	-0.82199	1.69625

H	-4.51451	-1.91787	-1.98226	3.85812	-2.53893	2.04747
H	-0.46195	3.25937	1.99563	3.85313	3.49274	0.30539
H	-1.89836	2.83611	2.94488	4.75713	2.02025	0.70332
H	-0.72343	1.5946	2.52065	4.92809	2.75024	-0.8878
H	2.88337	-1.56823	2.63103	-3.51077	-1.22899	-2.42251
H	0.56128	-3.3794	2.70392	-1.57803	-3.44423	-2.35102
H	0.65511	-3.84722	0.9892	-1.63824	-3.67566	-0.58773
H	2.1351	-3.78212	1.97488	-3.14525	-3.44776	-1.50518
H	5.15525	0.80316	-0.50201	-4.9661	2.01808	0.47889
H	5.38272	0.20454	-2.16963	-5.25027	1.64099	2.20178
H	4.09048	1.38116	-1.81377	-3.73118	2.43051	1.70064
		Con f.2- **4**c			Con f.2- **4**d	
C	-1.01039	1.73928	0.89075	-0.78111	1.69661	-1.02407
C	0.05942	2.81403	0.85804	0.25433	2.77773	-0.77134
C	0.76272	3.11779	-0.47922	0.68873	3.045	0.68267
C	1.86069	2.13997	-0.7876	1.50654	1.90802	1.22893
C	-2.45512	-1.22194	0.25406	-2.50655	-1.08713	0.82136
C	-2.66234	0.18915	-0.31131	-2.56921	0.08782	-0.16046
C	-1.48225	1.13811	-0.21258	-1.43792	1.07082	-0.03405
C	-0.19597	-1.75994	-0.86543	-0.07425	-1.73954	0.7988
C	3.19855	0.37487	-1.85815	2.12741	-0.06582	2.63372
C	2.84313	-1.0758	-1.43309	2.93427	-0.69185	1.47713
C	2.1332	-1.10516	-0.11629	2.23552	-0.85585	0.15595
C	0.71569	-1.40431	0.04718	0.88139	-1.36487	-0.05857
C	-1.62527	-2.17836	-0.62536	-1.48157	-2.20558	0.49911
C	2.01159	1.3135	-1.83365	1.27789	1.14788	2.30881
C	2.6327	-0.79105	1.10882	2.75441	-0.55072	-1.06549
C	1.5326	-0.86175	2.0674	1.74649	-0.84986	-2.07713
O	0.39173	-1.26677	1.39522	0.62963	-1.35151	-1.43737
C	-1.52431	1.47109	2.28409	-1.01208	1.4407	-2.49301
C	4.00675	-0.38431	1.51994	4.06598	0.03987	-1.45832
C	1.05342	1.19337	-2.98923	0.15749	1.37201	3.29092
O	1.49762	-0.6255	3.26265	1.76544	-0.71181	-3.28728
O	-2.1969	-2.25392	-1.9477	-1.6575	-2.67165	-0.84589
C	-1.62724	-3.58249	0.00686	-1.779	-3.42062	1.3878
O	-3.83953	0.68397	0.36039	-3.8186	0.74961	0.16345
C	-4.32965	1.90073	-0.17962	-4.24157	1.67357	-0.82718
H	0.82205	2.57148	1.61386	1.14888	2.54032	-1.36669
H	-0.41303	3.73843	1.22421	-0.13282	3.71298	-1.20387
H	1.21156	4.11706	-0.38357	-0.18527	3.26185	1.30425
H	0.03007	3.1885	-1.28869	1.29726	3.96091	0.67213
H	2.63779	2.10862	-0.02127	2.36003	1.65873	0.601
H	-3.45054	-1.67381	0.35108	-3.49789	-1.55512	0.81877
H	-2.02079	-1.17805	1.25417	-2.32737	-0.70898	1.83536
H	-2.91152	0.08885	-1.37717	-2.65262	-0.30676	-1.17944
H	-1.03302	1.38517	-1.16823	-1.21346	1.33794	0.99389
H	0.14414	-1.84939	-1.89122	0.17118	-1.69656	1.85246
H	3.63463	0.32805	-2.86444	2.8406	0.18124	3.43366
H	3.97548	0.74998	-1.1843	1.47439	-0.82575	3.0782
H	3.77261	-1.65249	-1.35911	3.28359	-1.67845	1.80981
H	2.23264	-1.55403	-2.20329	3.8351	-0.09854	1.29865
H	-1.79396	2.41291	2.78185	-0.07342	1.16951	-2.99171
H	-2.39907	0.82191	2.29715	-1.73624	0.64802	-2.68917
H	-0.73635	1.00972	2.89498	-1.3731	2.35478	-2.98474
H	4.74188	-0.63228	0.7499	4.61406	-0.63845	-2.12418
H	4.05739	0.69714	1.70311	3.91303	0.97556	-2.01086
H	4.29422	-0.8827	2.45285	4.69605	0.2496	-0.59122

H	0.26912	1.95395	-2.97112	0.5502	1.41002	4.3168
H	1.58923	1.28343	-3.94399	-0.39642	2.2964	3.11194
H	0.56427	0.21011	-2.99642	-0.55541	0.53569	3.26369
H	-3.1079	-2.57928	-1.84498	-1.1951	-2.06566	-1.44943
H	-1.07101	-4.28205	-0.62531	-1.04371	-4.21176	1.20734
H	-1.16654	-3.56247	0.99943	-1.74775	-3.14642	2.44689
H	-2.65802	-3.94387	0.10811	-2.77638	-3.8081	1.15538
H	-4.53836	1.80325	-1.25632	-5.19892	2.08237	-0.49006
H	-5.26208	2.12824	0.34549	-3.52749	2.49789	-0.95525
H	-3.62582	2.73075	-0.03493	-4.38595	1.18015	-1.80019
		Con f.2- **4**e			Con f.2- **4**f	
C	-2.15696	1.91581	-0.23683	1.4298	2.11248	-0.59747
C	-1.29333	3.15458	-0.08171	0.2941	2.5982	-1.4733
C	0.00281	3.03313	0.73376	-0.83658	3.3592	-0.72878
C	1.03813	2.22027	0.00371	-1.88925	2.46015	-0.13869
C	-2.1644	-1.73817	0.25133	2.55106	-0.81239	1.34943
C	-2.8774	-0.41034	0.51564	2.86931	0.16968	0.2161
C	-1.98914	0.80324	0.49689	1.69313	0.79983	-0.48222
C	-0.17681	-1.10487	-1.2212	0.13528	-1.45493	1.17682
C	3.2715	1.58233	-0.93755	-3.13801	1.09405	1.49399
C	2.67732	0.30262	-1.55173	-2.80371	-0.40938	1.66012
C	2.25203	-0.73409	-0.55304	-2.22826	-0.99934	0.41096
C	0.91761	-1.32477	-0.4797	-0.84477	-1.44445	0.26428
C	-1.48907	-1.85017	-1.13486	1.55421	-1.96768	1.10991
C	2.35122	2.4623	-0.1167	-1.95941	1.95854	1.10419
C	3.01854	-1.32883	0.40156	-2.85095	-1.18206	-0.78341
C	2.17971	-2.29046	1.11272	-1.86954	-1.73149	-1.71598
O	0.91442	-2.26658	0.55137	-0.66534	-1.88331	-1.04608
C	-3.22355	2.07975	-1.29206	2.24039	3.20925	0.04706
C	4.45822	-1.14676	0.75193	-4.25589	-0.90414	-1.19921
C	3.06754	3.62109	0.52944	-0.9223	2.18635	2.1724
O	2.4347	-3.04118	2.03749	-1.97053	-2.04257	-2.88873
O	-2.31272	-1.24243	-2.15124	1.72427	-2.78539	2.3002
C	-1.26636	-3.33013	-1.49136	1.89229	-2.8219	-0.11103
O	-3.4546	-0.56451	1.83222	3.68925	-0.53321	-0.74308
C	-4.45405	0.40273	2.11679	4.36864	0.33423	-1.63788
H	-1.04411	3.53052	-1.08513	0.72308	3.28188	-2.22048
H	-1.92848	3.93443	0.36573	-0.14188	1.75628	-2.02387
H	-0.22373	2.58426	1.71196	-1.32639	4.02233	-1.45236
H	0.37067	4.04182	0.94618	-0.39875	4.01056	0.03461
H	0.63131	1.34865	-0.49631	-2.69124	2.19003	-0.82764
H	-2.91714	-2.53188	0.33529	2.1922	-0.22902	2.20535
H	-1.42117	-1.91689	1.0335	3.49916	-1.27396	1.65108
H	-3.69591	-0.30694	-0.20277	3.49454	0.94819	0.66984
H	-1.17772	0.75737	1.22092	1.05643	0.10322	-1.02353
H	-0.10336	-0.36397	-2.00957	-0.12865	-1.0668	2.15587
H	3.68102	2.18612	-1.76114	-3.55373	1.44237	2.44934
H	4.14132	1.31802	-0.32291	-3.92827	1.19376	0.74333
H	3.44842	-0.14599	-2.19137	-3.72937	-0.94026	1.91311
H	1.84268	0.55789	-2.21052	-2.11864	-0.54982	2.49992
H	-4.01372	1.32874	-1.23897	3.12166	2.84492	0.57822
H	-3.68989	3.07029	-1.21378	2.57422	3.92669	-0.71476
H	-2.77453	2.02101	-2.29333	1.63856	3.7787	0.76588
H	5.04501	-0.83504	-0.11703	-4.31788	0.02116	-1.78695
H	4.5871	-0.38829	1.53495	-4.63536	-1.71379	-1.83289
H	4.87379	-2.08429	1.13644	-4.91502	-0.79911	-0.33329
H	2.39579	4.30646	1.05175	-0.18906	2.94571	1.89086

H	3.80643	3.25867	1.25813	-1.39411	2.4958	3.11488
H	3.62864	4.19838	-0.21873	-0.37089	1.26148	2.38511
H	-3.1522	-1.73289	-2.16966	1.11186	-3.53756	2.21624
H	-0.77437	-3.4143	-2.46564	1.20963	-3.67673	-0.17268
H	-0.64603	-3.82626	-0.73963	1.82677	-2.25958	-1.04209
H	-2.23278	-3.84612	-1.53864	2.91384	-3.19884	-0.00464
H	-4.83024	0.18646	3.12115	4.98439	-0.29709	-2.28511
H	-4.05503	1.426	2.09747	3.67357	0.91391	-2.25962
H	-5.28754	0.33975	1.4006	5.02095	1.03612	-1.09604
		Con f.1- **5**a			Con f.1- **5**b	
C	3.404	0.36466	0.14077	3.40629	-0.55411	-0.57287
C	3.34548	1.83518	-0.2124	3.57589	0.9012	-0.96607
C	2.00441	2.49503	0.16479	3.47453	1.9092	0.19403
C	0.79662	1.82981	-0.50263	2.08837	1.99801	0.85972
C	1.90883	-2.64769	-1.08556	0.65535	-3.0859	-0.89797
C	3.28768	-2.05371	-0.70962	2.16405	-2.76723	-0.89552
C	3.31039	-0.55746	-0.82994	2.45195	-1.31097	-1.14314
C	-0.31072	-1.77823	-0.41286	-1.23013	-1.64486	-0.23059
C	-0.93946	2.10791	1.38338	-0.14004	1.57294	-0.4568
C	-1.0479	0.59853	1.69094	-1.1669	1.47604	0.70183
C	-1.93674	-0.20249	0.78862	-2.30721	0.5611	0.40231
C	-1.54872	-1.61784	0.41955	-2.2163	-0.9279	0.64328
C	0.81058	-2.43196	-0.0678	-0.15632	-2.35417	0.14949
C	-0.50755	2.44093	-0.0277	1.00537	2.49208	-0.10699
C	3.51253	0.05452	1.61183	4.38355	-1.06684	0.45729
C	-3.14736	0.07399	0.2641	-3.5264	0.8586	-0.08554
C	-3.60388	-1.11582	-0.4723	-4.30861	-0.38424	-0.14631
O	-2.67553	-2.10548	-0.36609	-3.5494	-1.42271	0.30375
C	-4.02661	1.28112	0.35095	-4.11992	2.16312	-0.50258
C	-1.22221	3.25787	-0.80791	1.06798	3.73115	-0.60622
O	-4.6435	-1.28738	-1.08971	-5.46236	-0.55148	-0.50983
C	1.06481	-3.03028	1.2908	0.31296	-2.51296	1.57022
O	0.98132	1.94736	-1.91883	1.74606	0.7624	1.50649
H	4.1481	2.37525	0.3091	4.57162	1.02299	-1.41714
H	3.51503	1.96511	-1.28601	2.84487	1.16493	-1.73962
H	2.02088	3.5531	-0.12623	3.73363	2.90424	-0.18443
H	1.86686	2.46437	1.25068	4.20883	1.6668	0.97124
H	0.80329	0.76519	-0.23911	2.16292	2.71887	1.68093
H	2.03161	-3.73343	-1.21223	0.54103	-4.16669	-0.72825
H	1.59918	-2.24594	-2.05769	0.24306	-2.87467	-1.89155
H	4.02525	-2.47785	-1.40513	2.62493	-3.37359	-1.69
H	3.57666	-2.38766	0.29125	2.61121	-3.10503	0.04365
H	3.20065	-0.19034	-1.85235	1.8151	-0.83801	-1.89275
H	-0.39551	-1.35402	-1.41263	-1.46789	-1.57277	-1.2914
H	-1.89256	2.60097	1.58978	0.22545	0.56898	-0.69213
H	-0.21687	2.5224	2.09873	-0.64972	1.9508	-1.34938
H	-0.05201	0.1422	1.69036	-0.64767	1.12264	1.5972
H	-1.42337	0.48876	2.71848	-1.54907	2.48059	0.91085
H	-1.50908	-2.23294	1.32363	-2.06206	-1.13729	1.70535
H	2.6072	0.37184	2.14725	4.16232	-0.65159	1.44831
H	3.66137	-1.00845	1.81519	4.37943	-2.15579	0.54166
H	4.34811	0.60687	2.06304	5.40422	-0.752	0.2033
H	-3.78758	2.00377	-0.43782	-3.43392	2.99384	-0.31742
H	-5.07295	0.98612	0.22135	-4.36396	2.1527	-1.57241
H	-3.92708	1.78842	1.31372	-5.05544	2.35541	0.03675
H	-2.14858	3.7079	-0.46086	0.3166	4.10239	-1.29981
H	-0.89782	3.51124	-1.81298	1.8654	4.42238	-0.33957

H	0.17052	-3.08239	1.91678	-0.38313	-2.10012	2.30411
H	1.47522	-4.04323	1.19276	0.471	-3.57427	1.80257
H	1.80994	-2.43945	1.83759	1.27882	-2.01371	1.71658
H	0.24004	1.48173	-2.34174	1.79127	0.05668	0.83483
		Con f.1- **5**c			Con f.1- **5**d	
C	-2.26964	1.56077	1.12723	-3.52965	-0.88318	-0.10676
C	-1.8285	2.93396	0.6408	-3.99663	0.55348	0.00568
C	-0.83123	2.84411	-0.52926	-3.00863	1.55958	-0.62656
C	0.43557	2.0467	-0.20469	-1.90325	1.98788	0.34287
C	-3.29791	-1.53571	-0.59853	-0.83744	-2.97142	1.23992
C	-3.57603	-0.58545	0.58014	-2.35654	-2.89109	0.96463
C	-3.06603	0.81063	0.35116	-2.88889	-1.48591	0.90879
C	-0.87149	-1.66344	0.00631	1.02103	-1.49587	0.53879
C	0.7389	0.55118	-2.25545	0.34486	1.85493	-0.85551
C	1.57278	-0.72888	-1.98312	1.34114	1.61696	0.30582
C	1.58216	-1.19481	-0.56081	2.37034	0.57453	0.02745
C	0.54353	-2.16285	-0.03758	1.97027	-0.81628	-0.40116
C	-1.87981	-2.03157	-0.79651	0.03529	-2.33529	0.18141
C	1.21952	1.75017	-1.46698	-0.79146	2.72656	-0.38026
C	-1.703	1.13394	2.45635	-3.84593	-1.5292	-1.43225
C	2.46687	-0.9245	0.41872	3.70955	0.63792	0.14797
C	2.07037	-1.68604	1.61252	4.25704	-0.69137	-0.16359
O	0.95865	-2.42187	1.33632	3.2408	-1.54416	-0.46861
C	3.69587	-0.07657	0.43826	4.61217	1.76038	0.54017
C	2.26271	2.49174	-1.84862	-0.80579	4.05212	-0.5436
O	2.60346	-1.72677	2.71045	5.42155	-1.06015	-0.16921
C	-1.72748	-2.96489	-1.96969	-0.2538	-2.73848	-1.24081
O	1.16926	2.80521	0.7621	-2.53932	2.78586	1.34995
H	-2.69583	3.51502	0.30353	-4.96771	0.65291	-0.49639
H	-1.37428	3.49465	1.46547	-4.15274	0.82752	1.05468
H	-0.525	3.84846	-0.85057	-3.53318	2.47104	-0.93986
H	-1.33106	2.37219	-1.38218	-2.55571	1.12834	-1.52668
H	0.13934	1.08605	0.23559	-1.47717	1.08515	0.80612
H	-3.63545	-1.06128	-1.53279	-0.57166	-4.03712	1.30772
H	-3.93396	-2.42655	-0.48876	-0.61543	-2.52584	2.21674
H	-3.18226	-1.01594	1.5065	-2.85744	-3.43122	1.78119
H	-4.66849	-0.54463	0.70237	-2.58992	-3.44024	0.04836
H	-3.40082	1.25191	-0.59127	-2.73861	-0.90567	1.82171
H	-1.04229	-0.94407	0.79893	1.17862	-1.28046	1.59441
H	0.80474	0.75501	-3.32956	0.87156	2.31771	-1.69643
H	-0.31211	0.34561	-2.02739	-0.04956	0.8919	-1.20259
H	1.17534	-1.53295	-2.61437	1.82019	2.56482	0.56714
H	2.60328	-0.54082	-2.30158	0.77344	1.29411	1.19025
H	0.62492	-3.11364	-0.57343	1.58045	-0.80258	-1.42232
H	-2.07949	1.78416	3.25902	-3.46053	-0.9223	-2.2627
H	-1.9453	0.1018	2.72257	-3.44096	-2.53759	-1.53857
H	-0.61016	1.24368	2.46147	-4.93441	-1.5874	-1.57388
H	4.57283	-0.68103	0.70214	4.05727	2.68793	0.70224
H	3.872	0.40114	-0.52735	5.15529	1.51796	1.4622
H	3.61429	0.70887	1.19996	5.36573	1.93813	-0.23692
H	2.8052	2.28287	-2.76848	-0.00925	4.56762	-1.07582
H	2.61279	3.32766	-1.24892	-1.60918	4.66503	-0.14327
H	-0.73999	-3.42814	-2.03457	-1.14491	-2.22343	-1.61768
H	-2.48101	-3.76221	-1.93152	0.57278	-2.52558	-1.92223
H	-1.89691	-2.41752	-2.90718	-0.46321	-3.81388	-1.29346
H	1.95576	2.28608	1.00008	-1.85017	3.05317	1.98103
		Con f.1- **5**e				

C	-3.34498	-0.71957	0.23105
C	-3.33431	0.78724	0.35696
C	-2.541	1.435	-0.80012
C	-1.8991	2.76435	-0.41393
C	-0.83988	-3.22547	1.07285
C	-2.36074	-2.93882	1.04897
C	-2.63303	-1.46292	1.09273
C	0.96052	-1.72957	0.28696
C	0.1074	1.51874	0.72581
C	1.02478	1.45337	-0.52141
C	2.11252	0.43805	-0.37586
C	1.89791	-1.03319	-0.65445
C	-0.05911	-2.54373	-0.03074
C	-0.8896	2.65597	0.71815
C	-4.12063	-1.26115	-0.94177
C	3.39246	0.63724	-0.00726
C	4.09323	-0.65326	-0.06627
O	3.22737	-1.62255	-0.46978
C	4.11299	1.88233	0.39117
C	-0.90623	3.56703	1.69793
O	5.26411	-0.90466	0.17479
C	-0.50693	-2.85676	-1.43259
O	-1.27033	3.27045	-1.61104
H	-4.35671	1.18986	0.36366
H	-2.87709	1.06497	1.31325
H	-3.18888	1.60865	-1.66627
H	-1.75377	0.74842	-1.13121
H	-2.68566	3.46616	-0.10068
H	-0.69526	-4.31237	0.98705
H	-0.43309	-2.92362	2.04503
H	-2.80162	-3.43757	1.92435
H	-2.81185	-3.40228	0.16699
H	-2.1276	-0.93623	1.90485
H	1.19775	-1.56657	1.33749
H	-0.42572	0.56507	0.81198
H	0.73602	1.60929	1.6182
H	0.42841	1.21855	-1.40812
H	1.47228	2.4384	-0.68677
H	1.62718	-1.19114	-1.70149
H	-5.17156	-0.9452	-0.87881
H	-3.73587	-0.86165	-1.88943
H	-4.10237	-2.35172	-1.00399
H	3.46426	2.75996	0.33169
H	4.48411	1.79994	1.42049
H	4.98649	2.04888	-0.25098
H	-0.18874	3.54471	2.51575
H	-1.64228	4.36864	1.71698
H	-1.48984	-2.41164	-1.62979
H	0.18144	-2.49259	-2.19899
H	-0.62027	-3.94089	-1.56163
H	-0.83664	4.10672	-1.3703

5 The 1D and 2D NMR spectra of 1–5

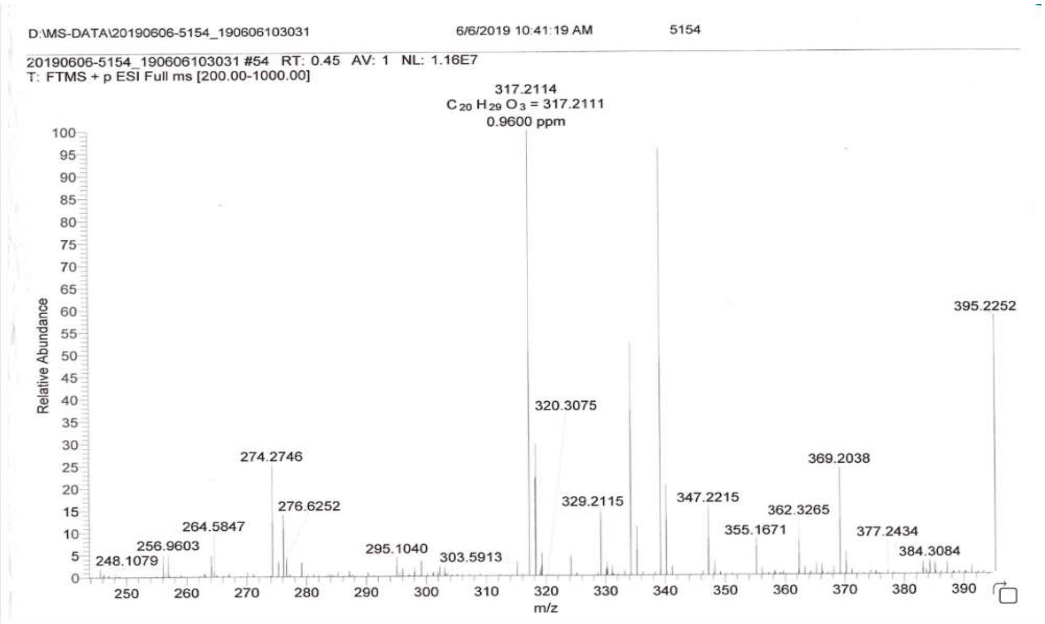

Figure S10. The positive HRESIMS spectrum of compound **1.**

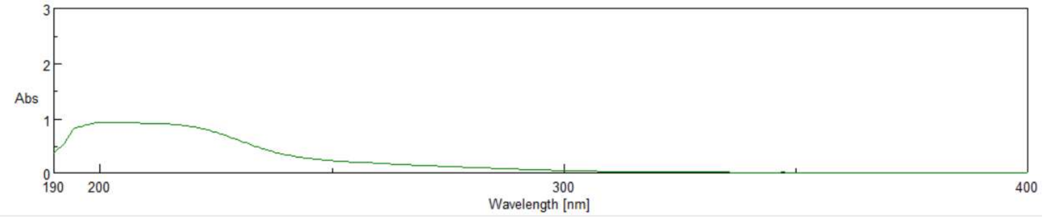

Figure S11. UV spectrum of compound **1.**

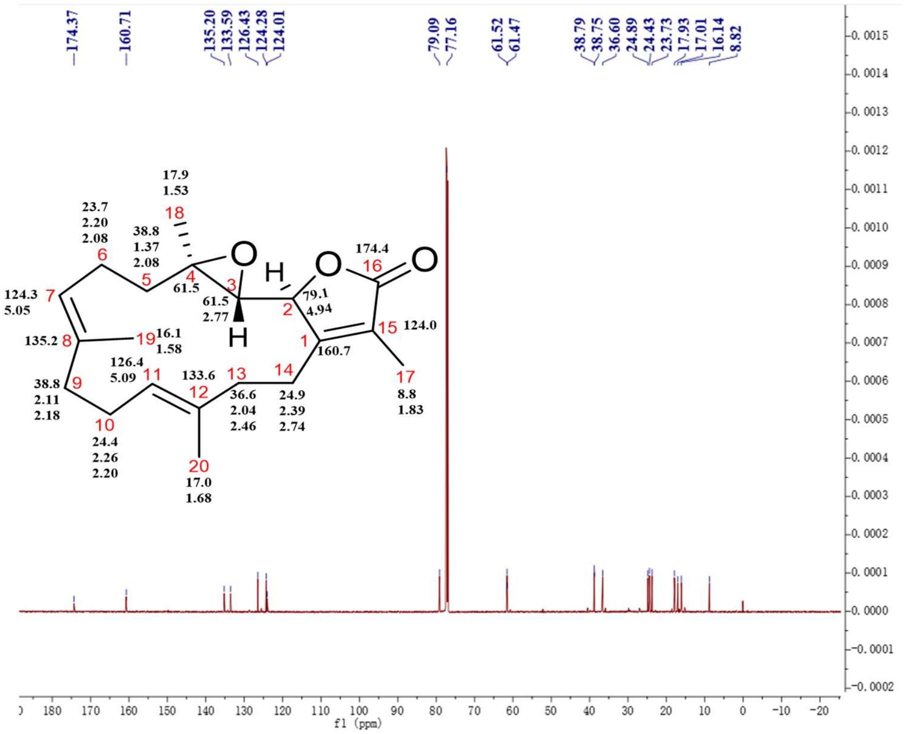

Figure S12. ^{1}H NMR spectrum (600 MHz, $CDCl_3$) of compound **1.**

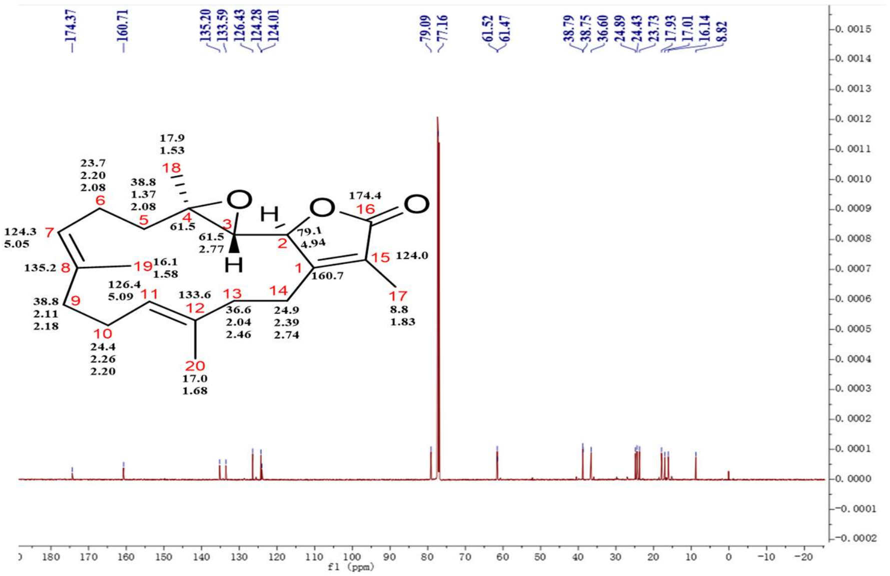

Figure S13. ^{13}C NMR spectrum 150 MHz, $CDCl_3$) of compound **1.**

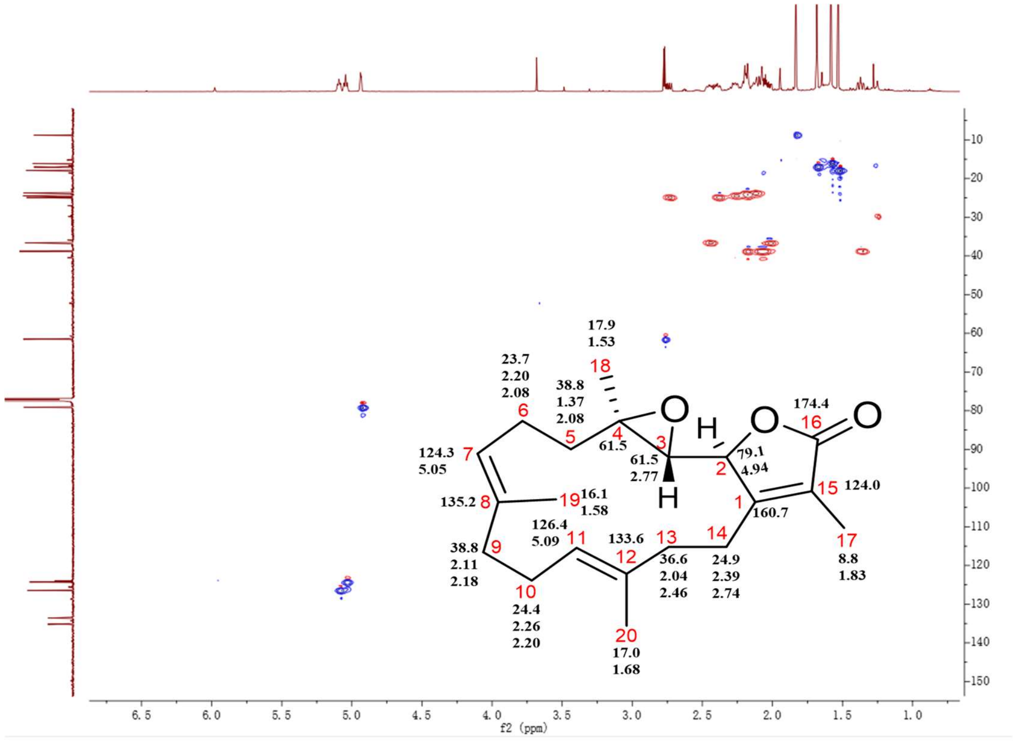

Figure S14. HSQC (600 M Hz, $CDCl_3$) of compound **1.**

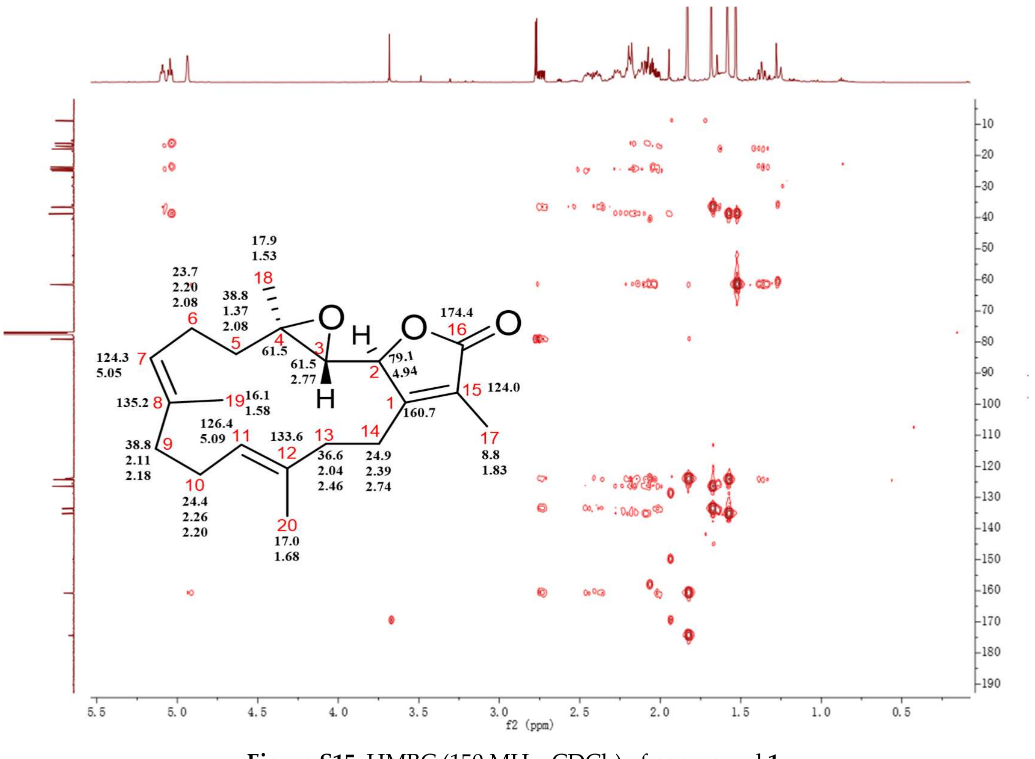

Figure S15. HMBC (150 MHz, $CDCl_3$) of compound **1.**

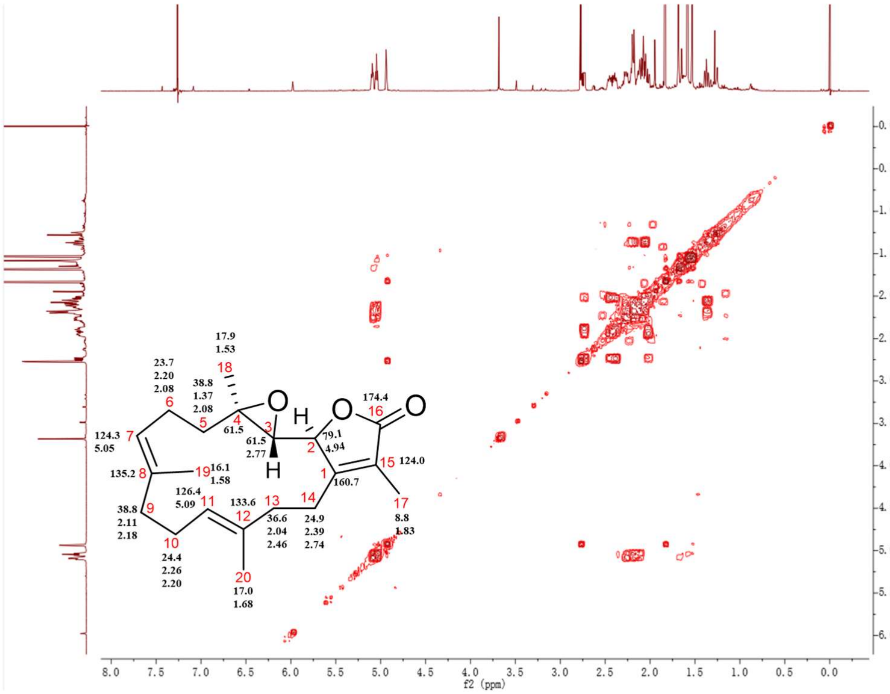

Figure S16. ^{1}H-^{1}H COSY (600 MHz, $CDCl_3$) of compound **1.**

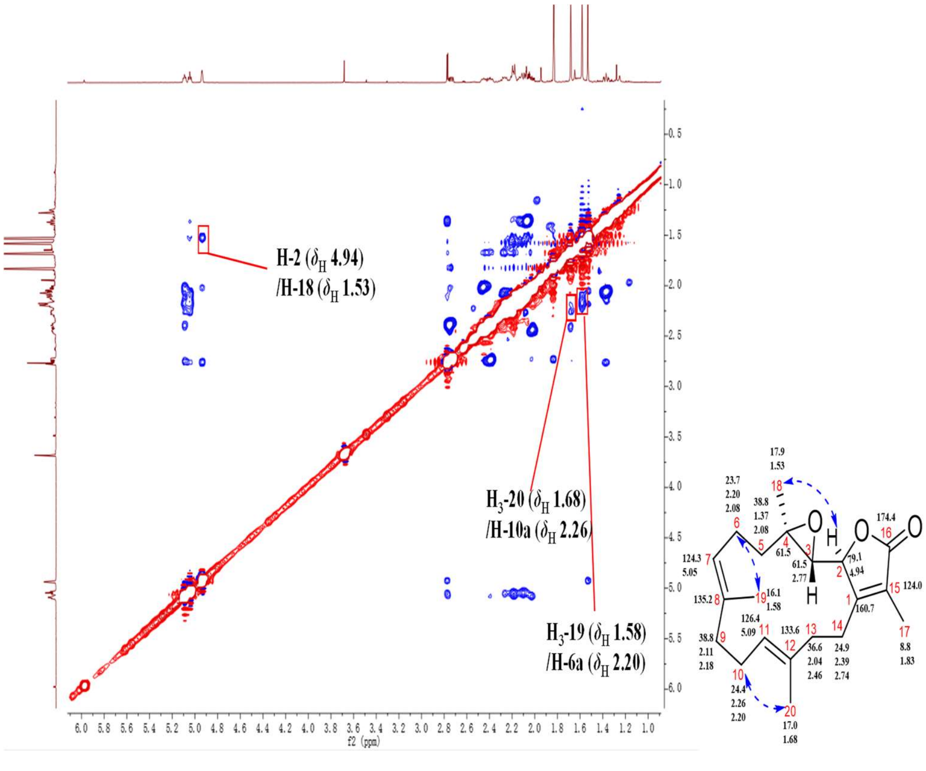

Figure S17. NOESY (600 MHz, $CDCl_3$) of compound **1.**

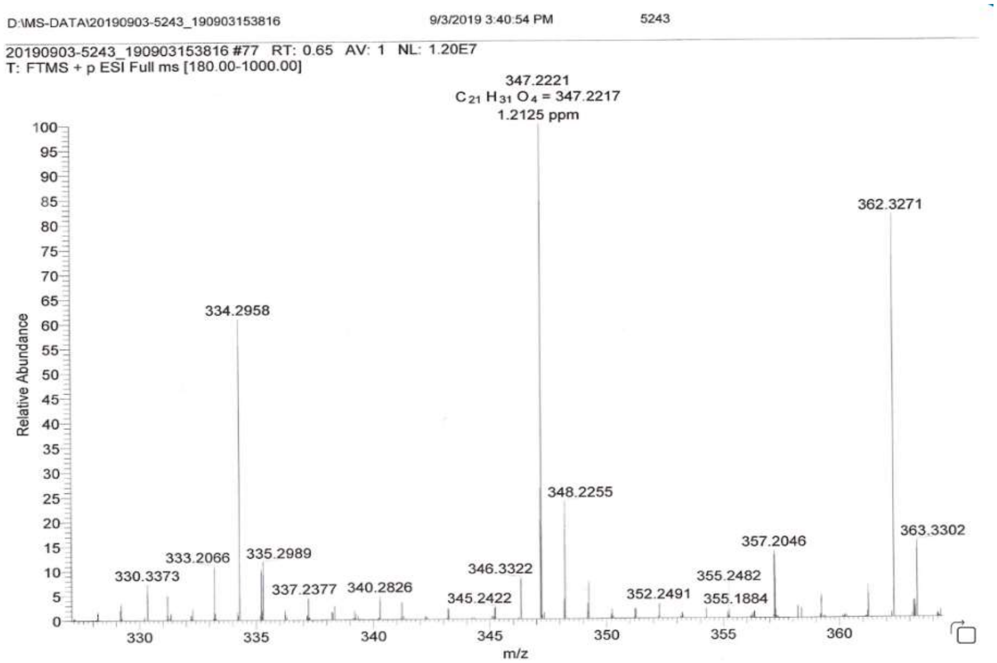

Figure S18. The positive HRESIMS spectrum of compound **2.**

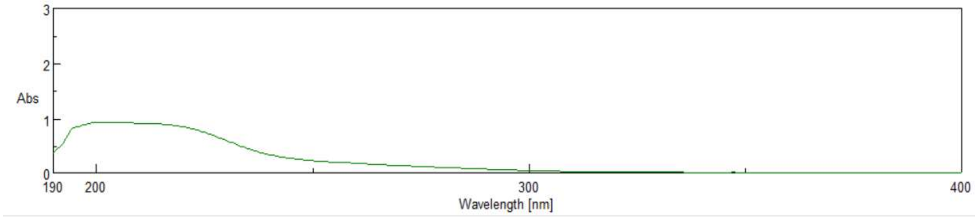

Figure S19. UV spectrum of compound **2.**

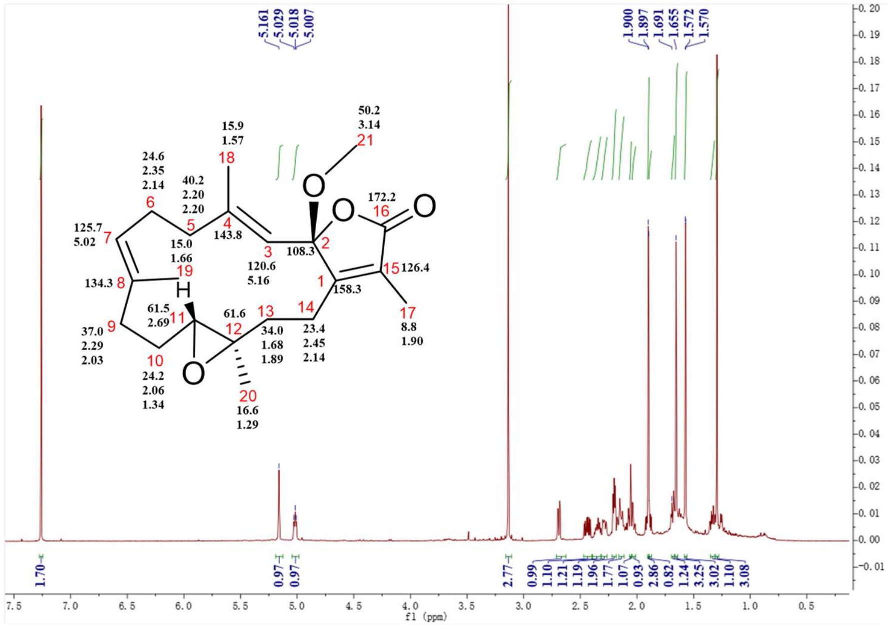

Figure S20. ^{1}H NMR spectrum (600 MHz, $CDCl_3$) of compound **2.**

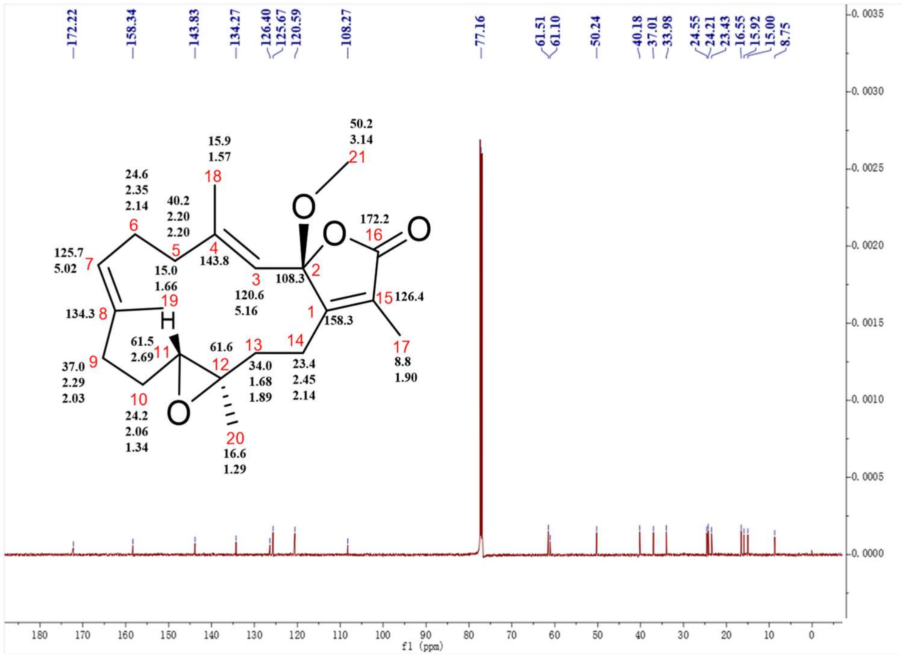

Figure S21. ^{13}C NMR spectrum (150 MHz, $CDCl_3$) of compound **2.**

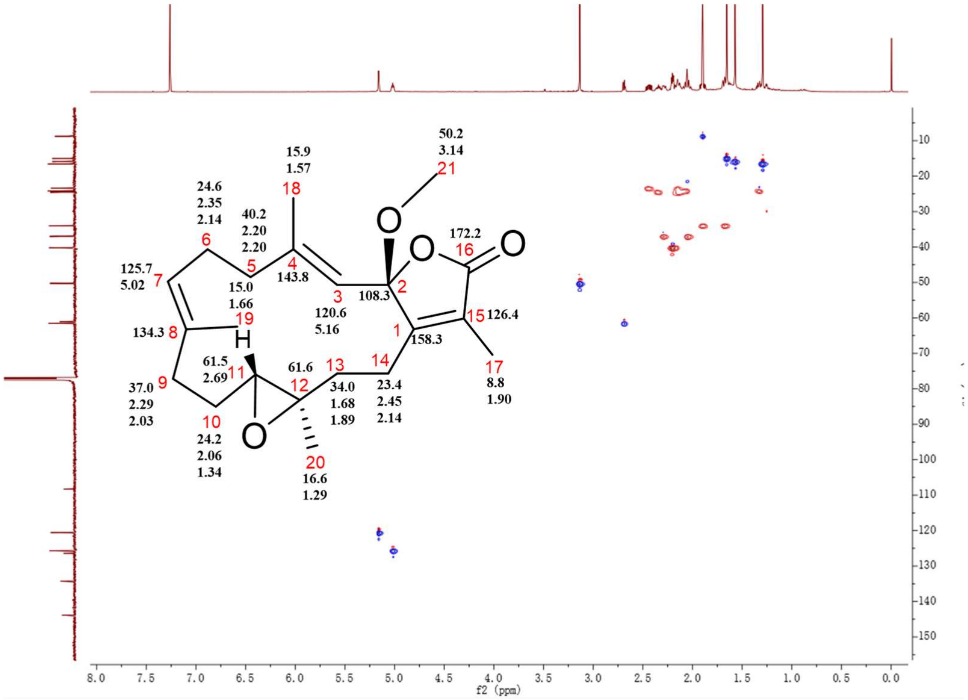

Figure S22. HSQC (600 MHz, $CDCl_3$) of compound **2.**

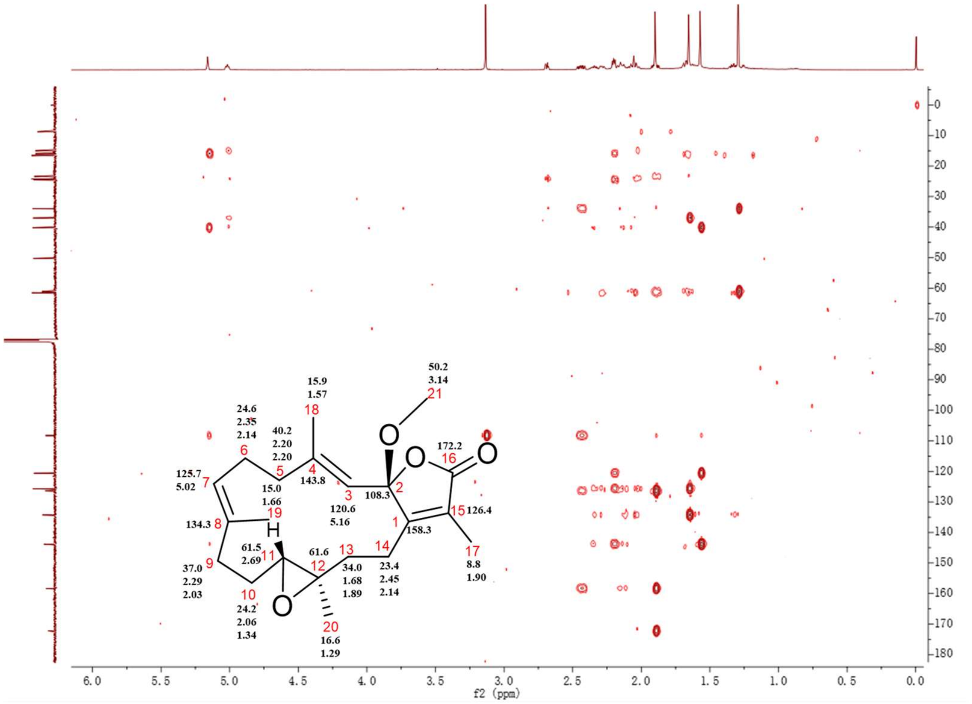

Figure S23. HMBC (150 MHz, $CDCl_3$) of compound **2.**

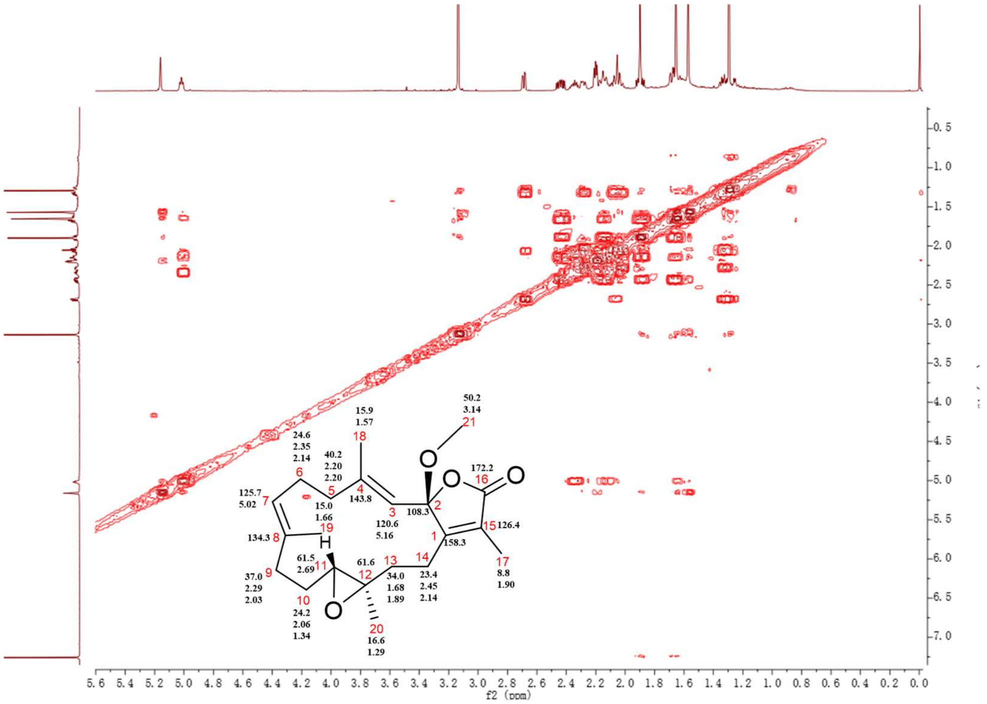

Figure S24. ^1H-^1H COSY (600 MHz, $CDCl_3$) of compound **2.**

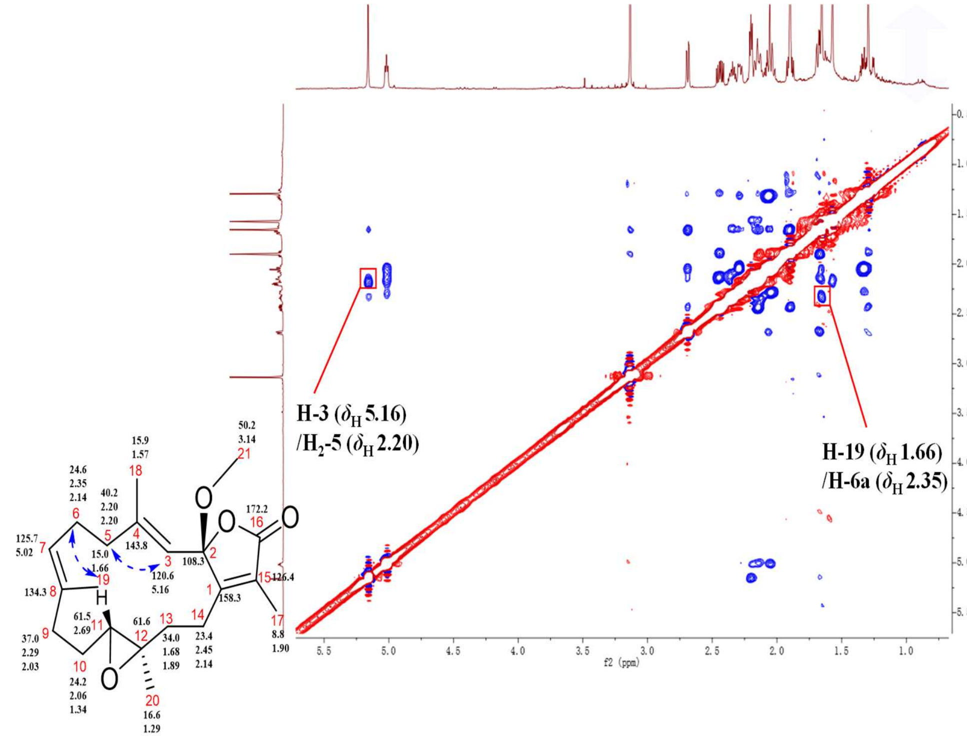

Figure S25. NOESY (600 MHz, CDCl3) of compound **2.**

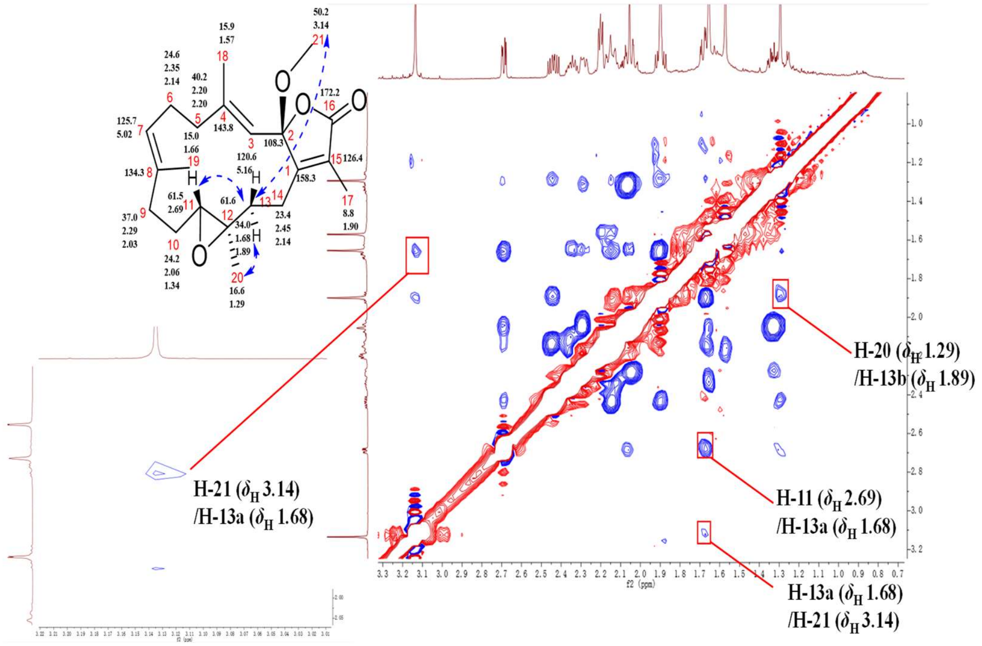

Figure S26 NOESY (500 MHz, CDCl3) of compound 2

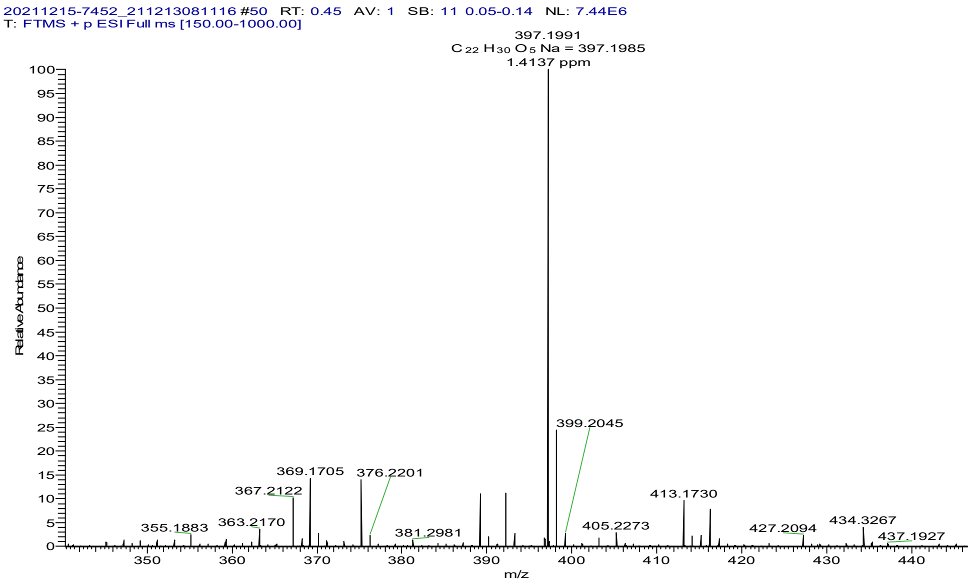

Figure S27. The positive HRESIMS spectrum of compound 3

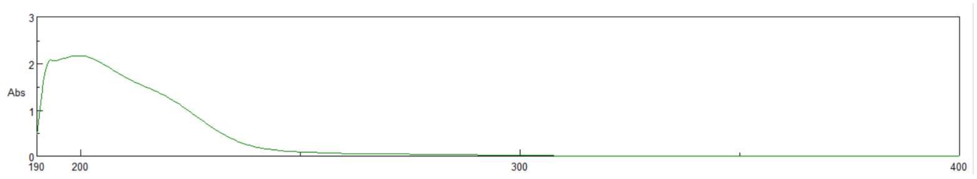

Figure S28. UV spectrum of compound 3

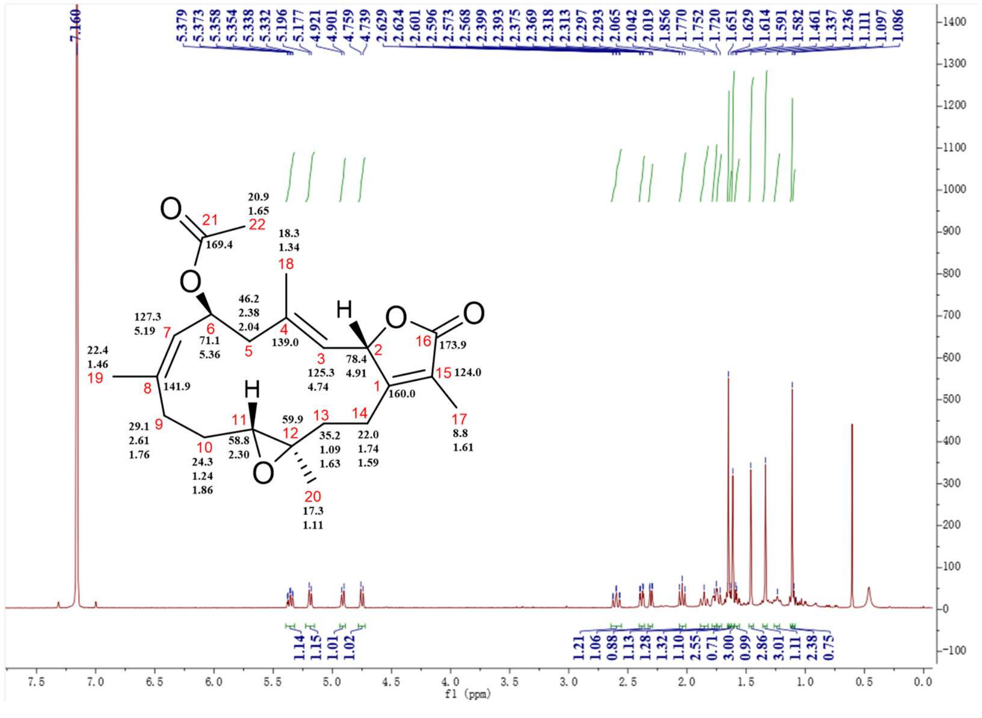

Figure S29. ^{1}H NMR spectrum (500 MHz, C_6D_6) of compound 3

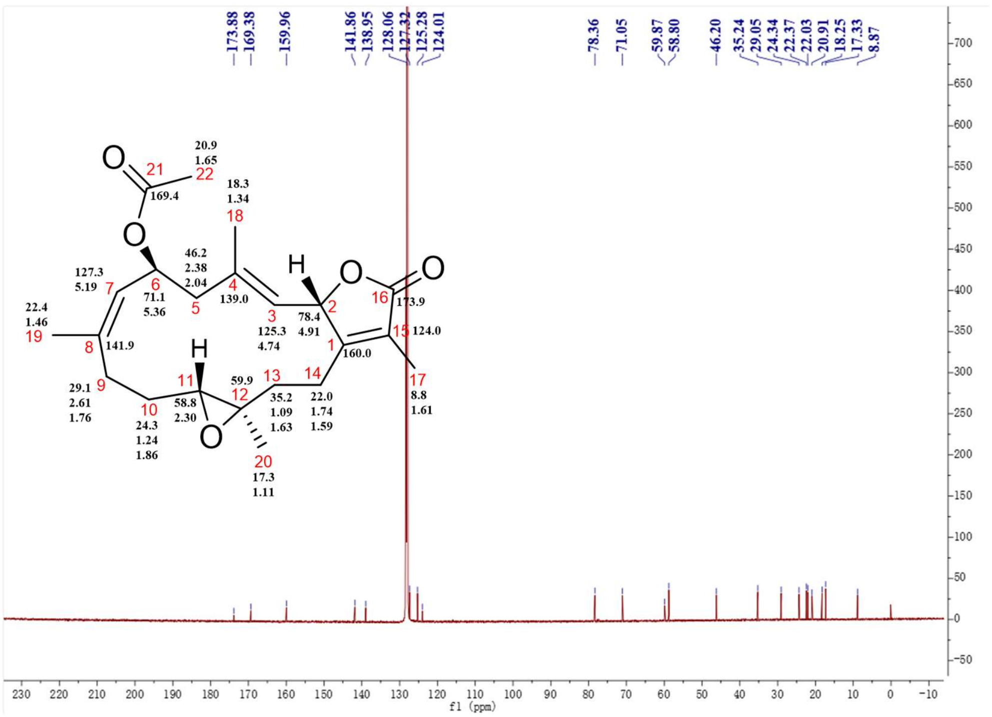

Figure S30. ^{13}C NMR spectrum (125 MHz, C_6D_6) of compound 3

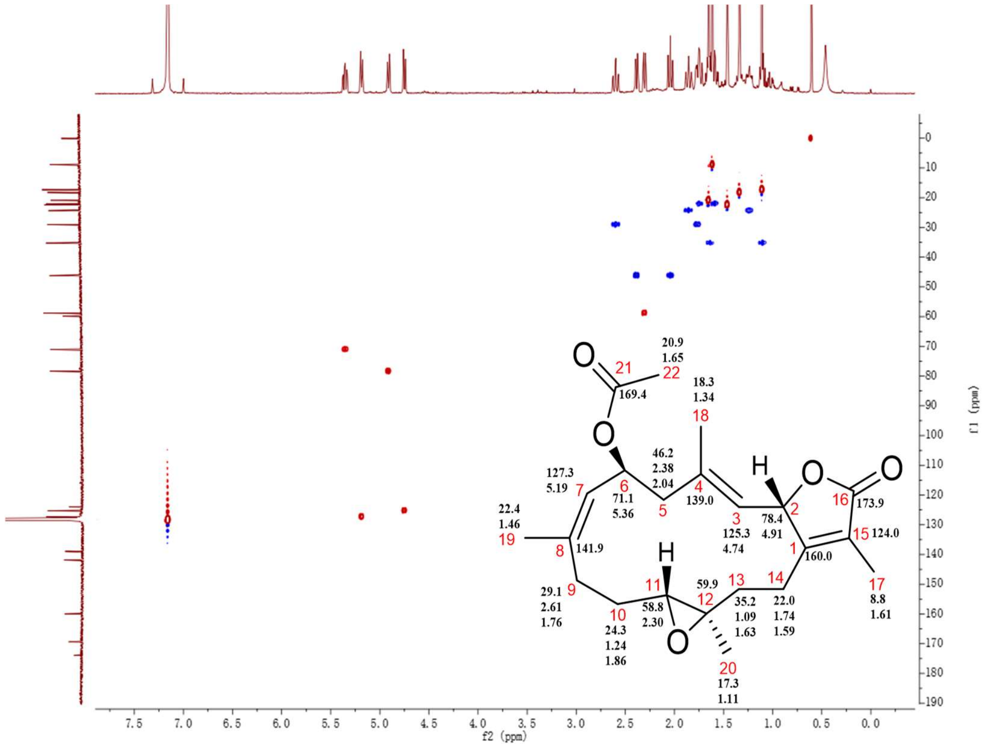

Figure S31. HSQC (500 M Hz, C_6D_6) of compound 3

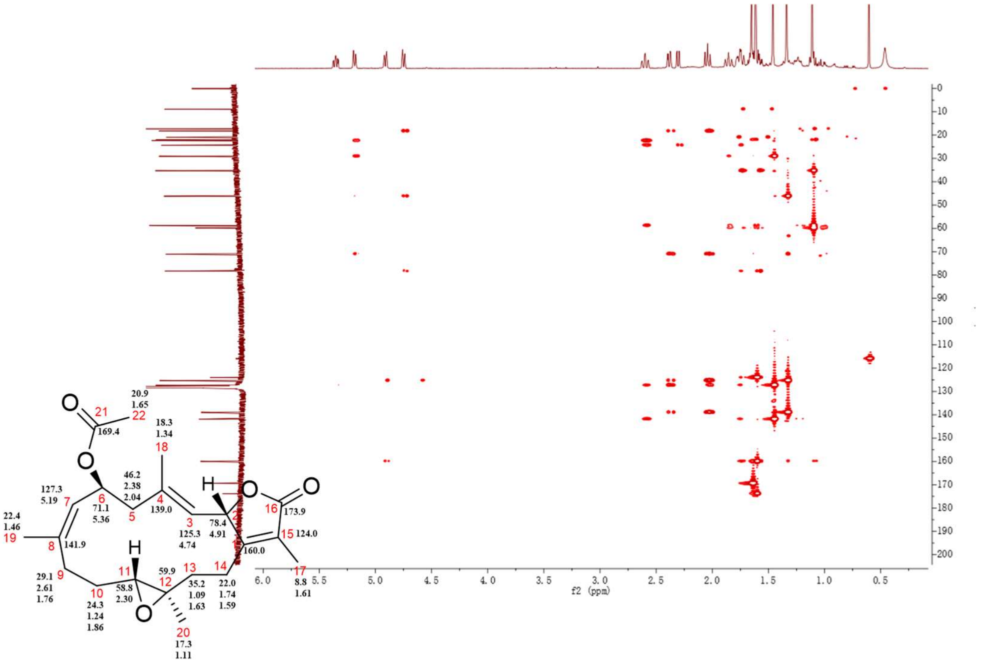

Figure S32. HMBC (125 MHz, C_6D_6) of compound **3.**

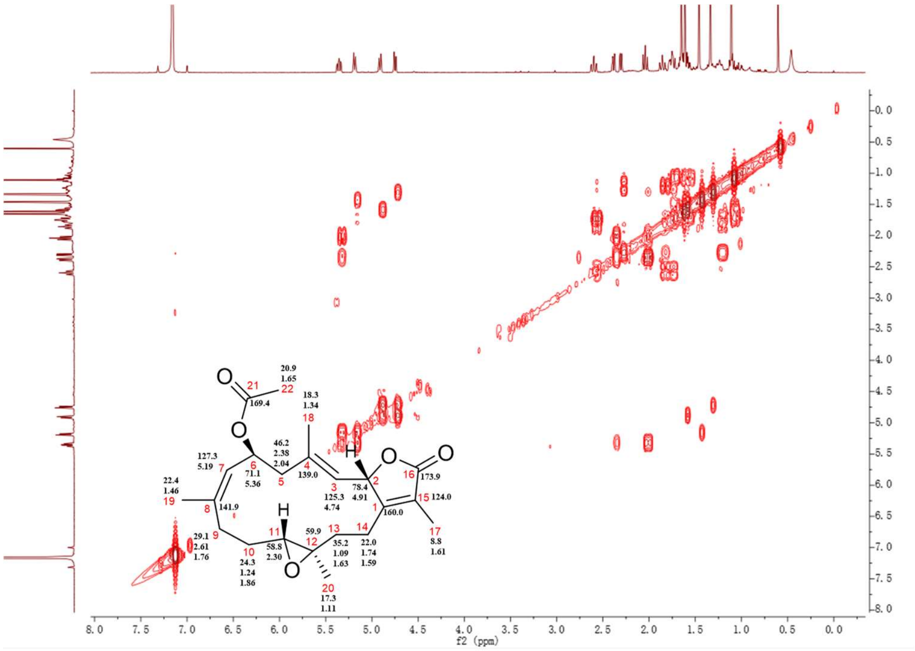

Figure S33. ^1H-^1H COSY (500 MHz, C_6D_6) of compound **3.**

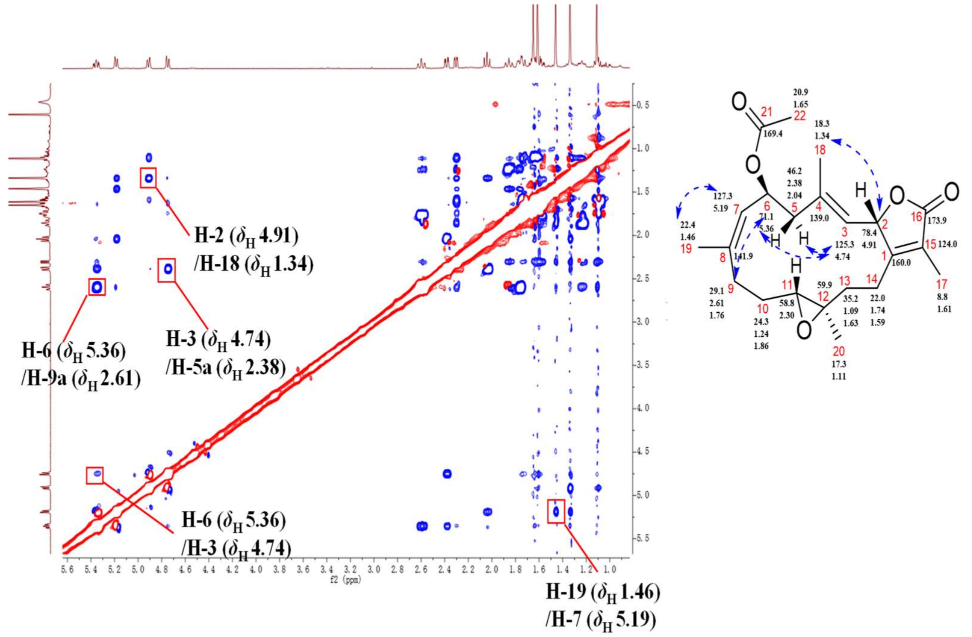

Figure S34. NOESY (500 MHz, C_6D_6) of compound **3.**

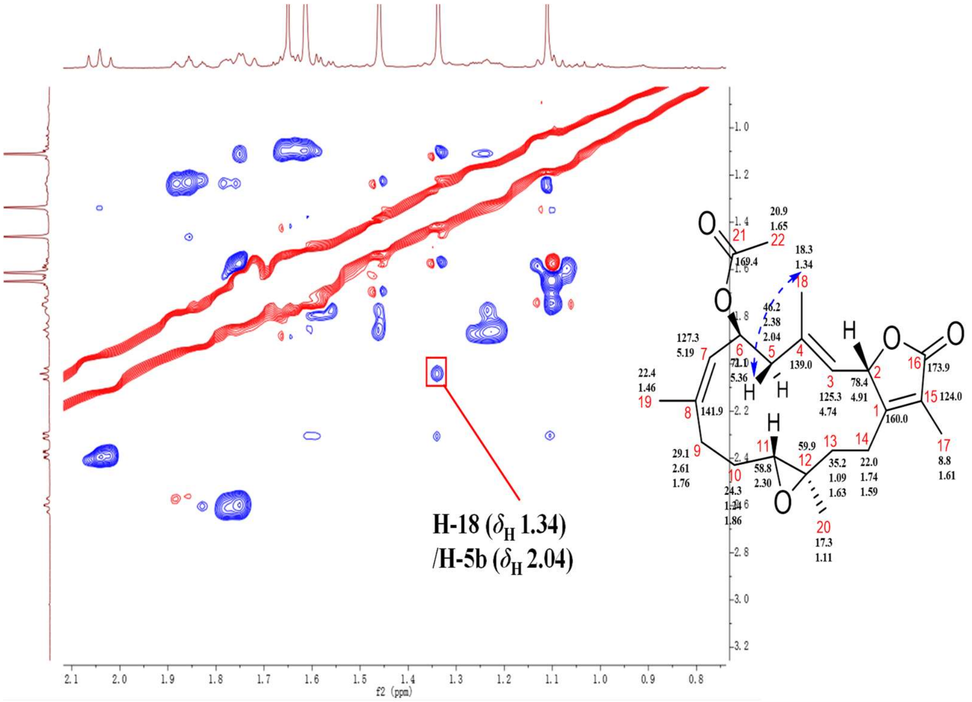

Figure S35. NOESY (500 MHz, C_6D_6) of compound **3.**

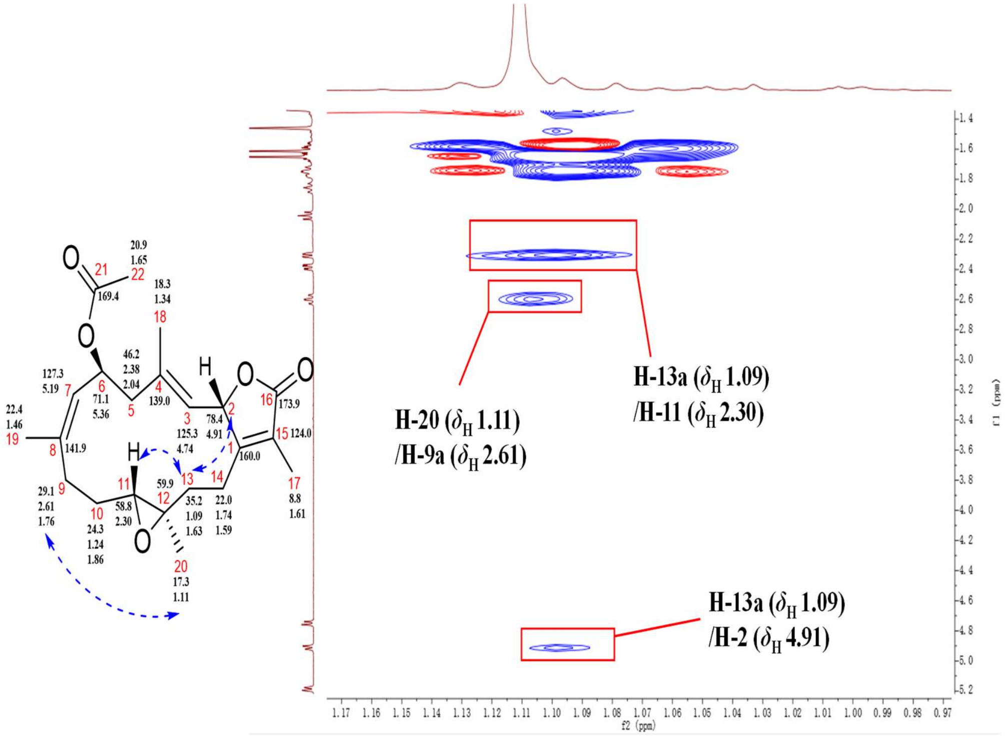

Figure S36. NOESY (500 MHz, C_6D_6) of compound **3.**

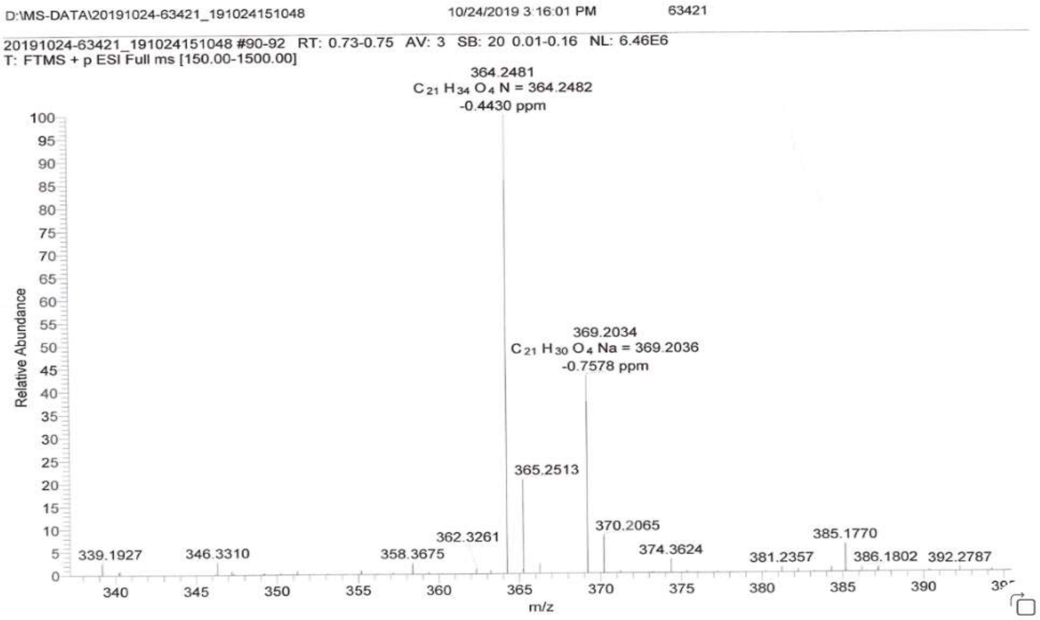

Figure S37. The positive HRESIMS spectrum of compound **4.**

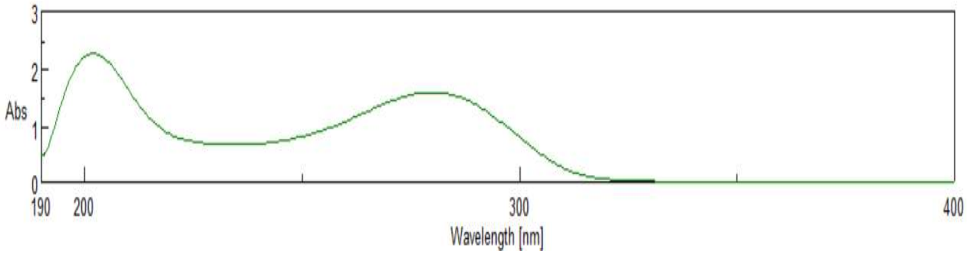

Figure S38. UV spectrum of compound **4.**

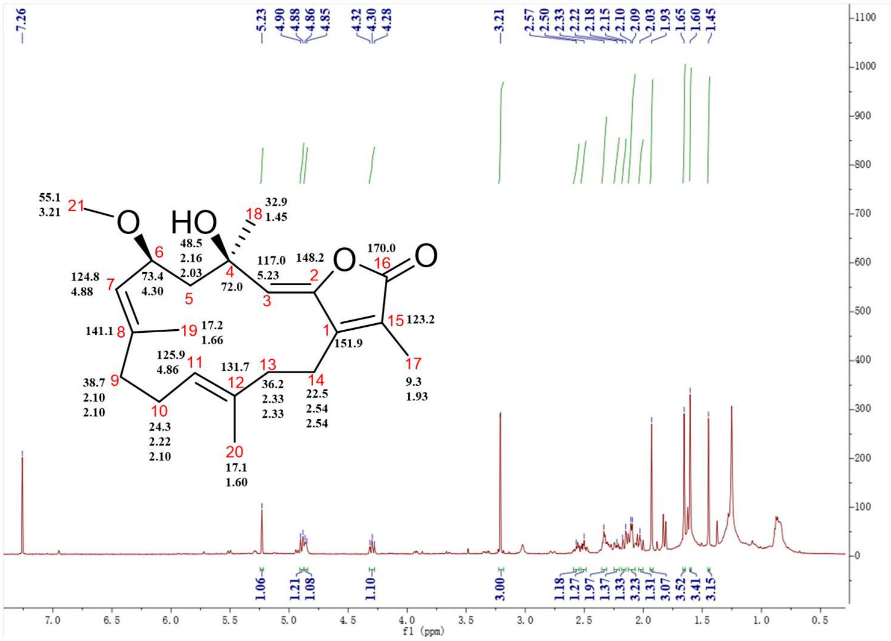

Figure S39. ${}^{1}H$ NMR spectrum (500 MHz, $CDCl_3$) of compound **4.**

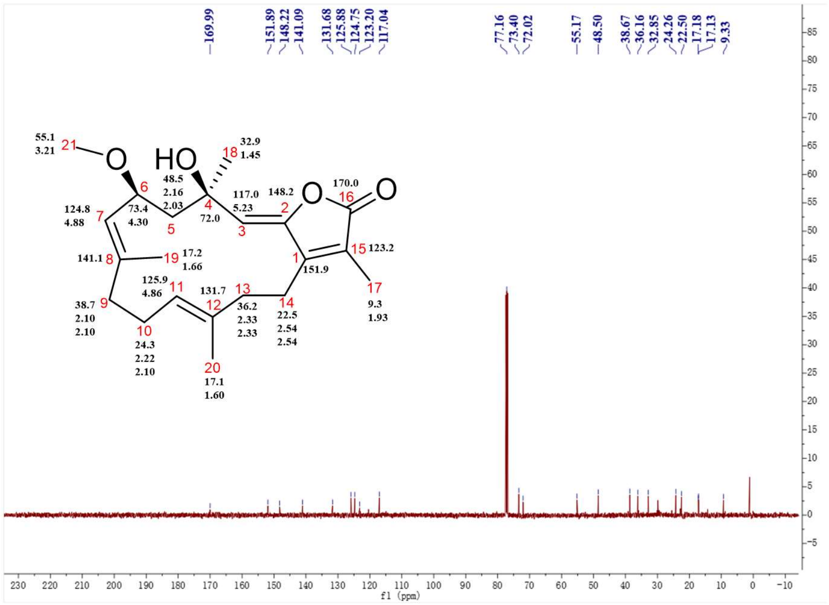

Figure S40. ${}^{13}C$ NMR spectrum 125 MHz, $CDCl_3$) of compound **4.**

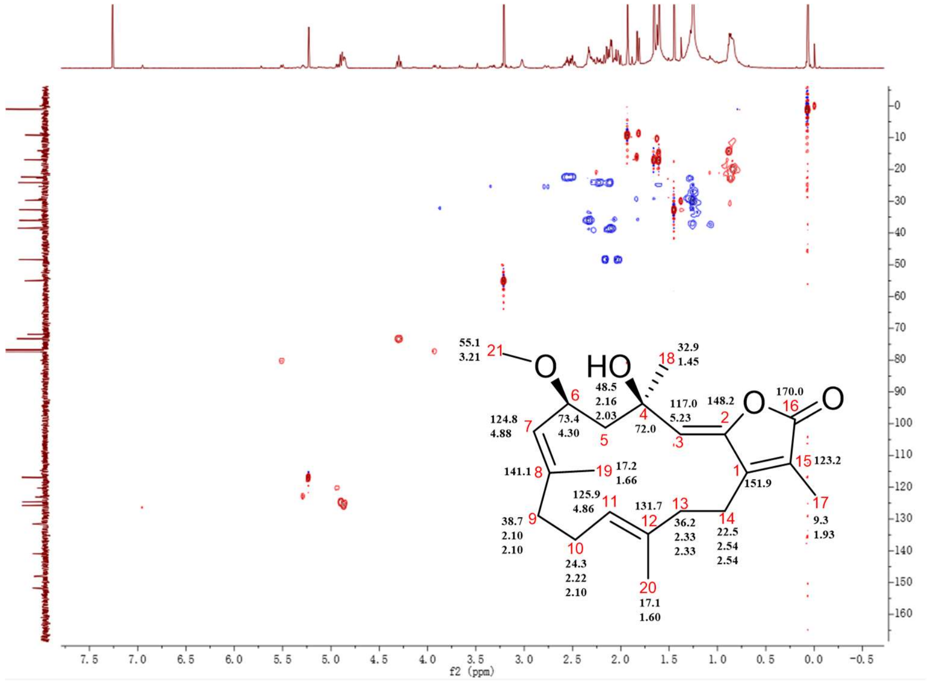

Figure S41. HSQC (500 MHz, $CDCl_3$) of compound **4.**

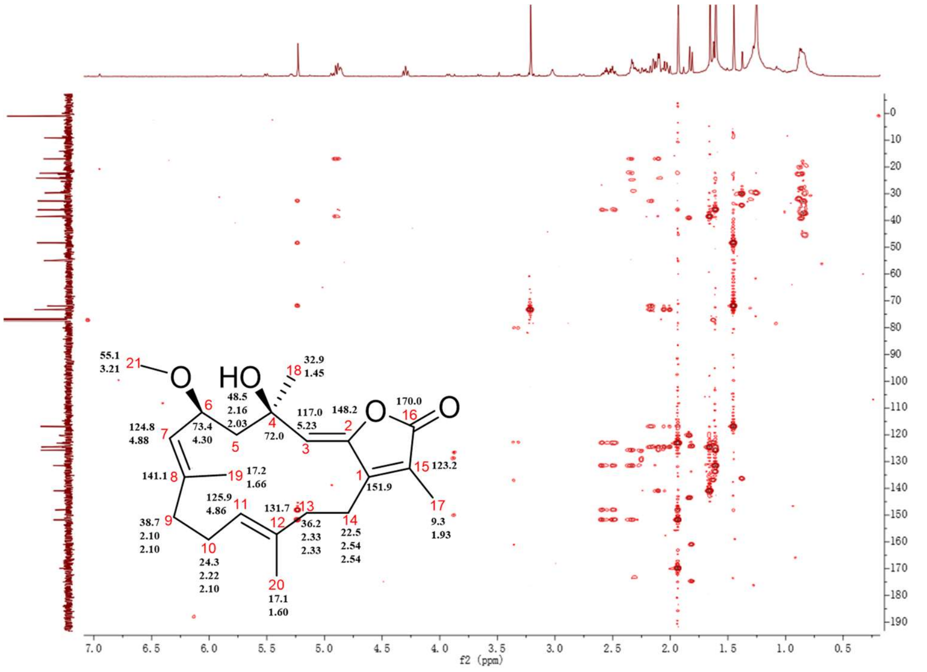

Figure S42. HMBC (125 MHz, $CDCl_3$) of compound **4.**

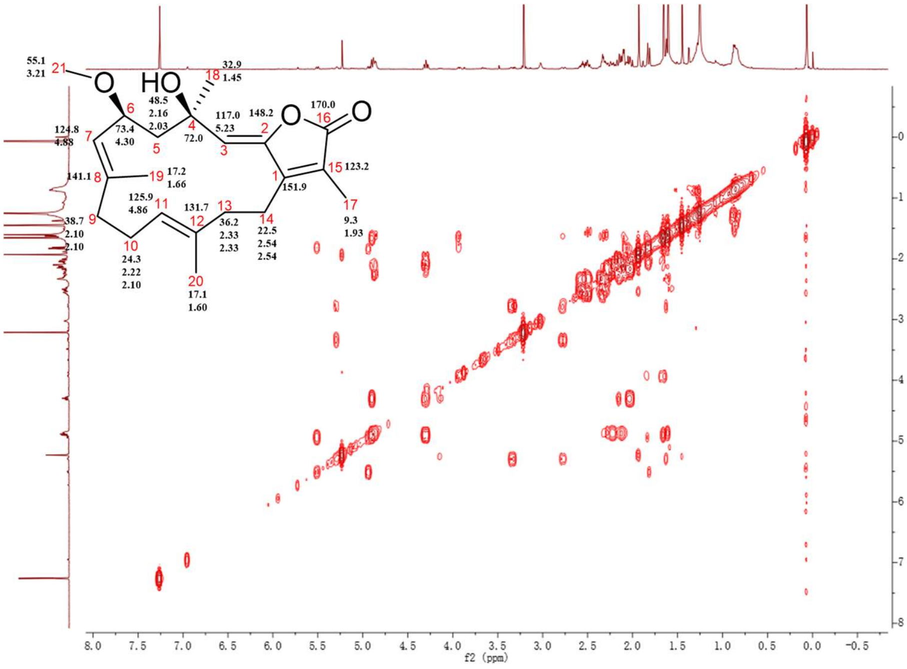

Figure S43. ^{1}H-^{1}H COSY (500 MHz, $CDCl_3$) of compound **4.**

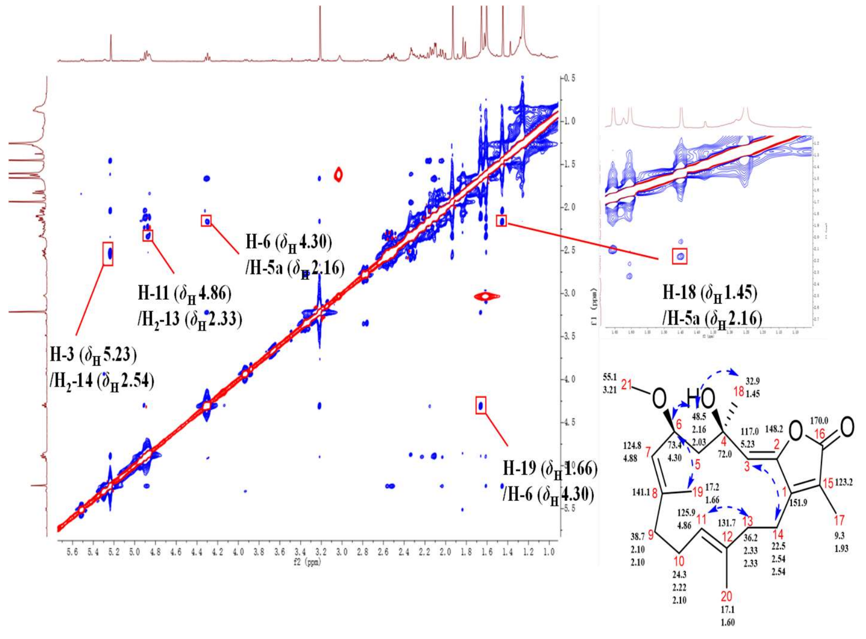

Figure S44. NOESY (500 MHz, $CDCl_3$) of compound **4.**

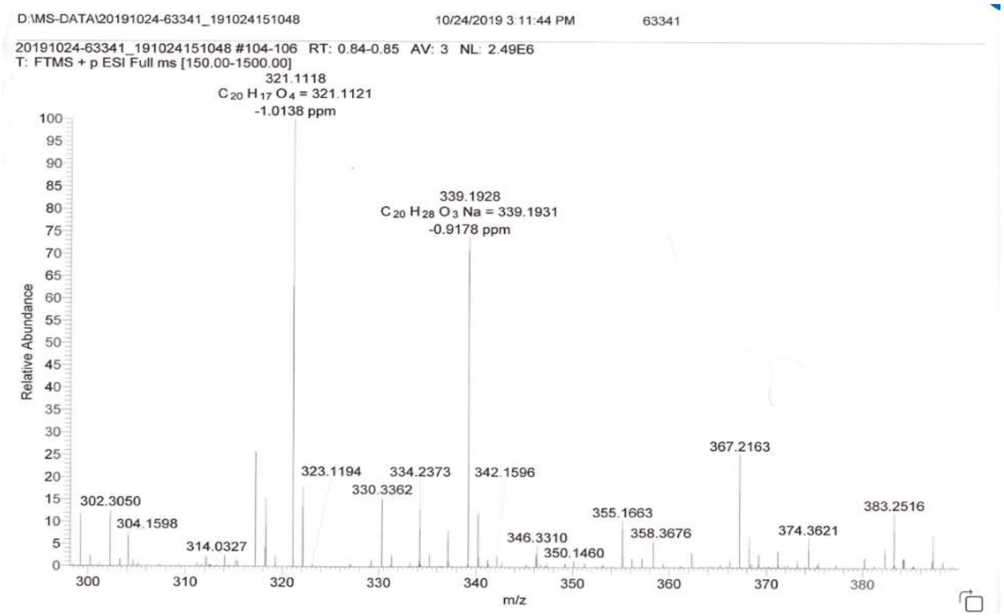

Figure S45. The positive HRESIMS spectrum of compound **5.**

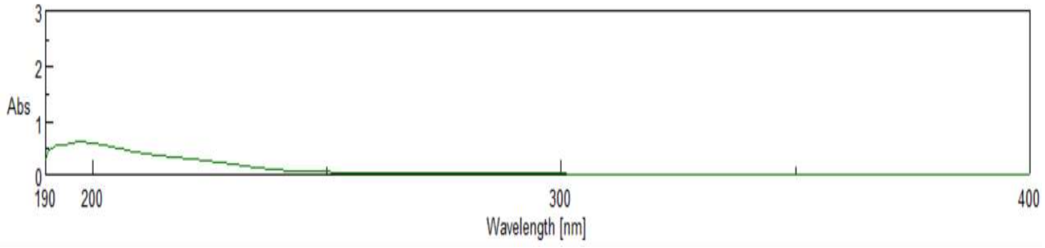

Figure 46. UV spectrum of compound **5.**

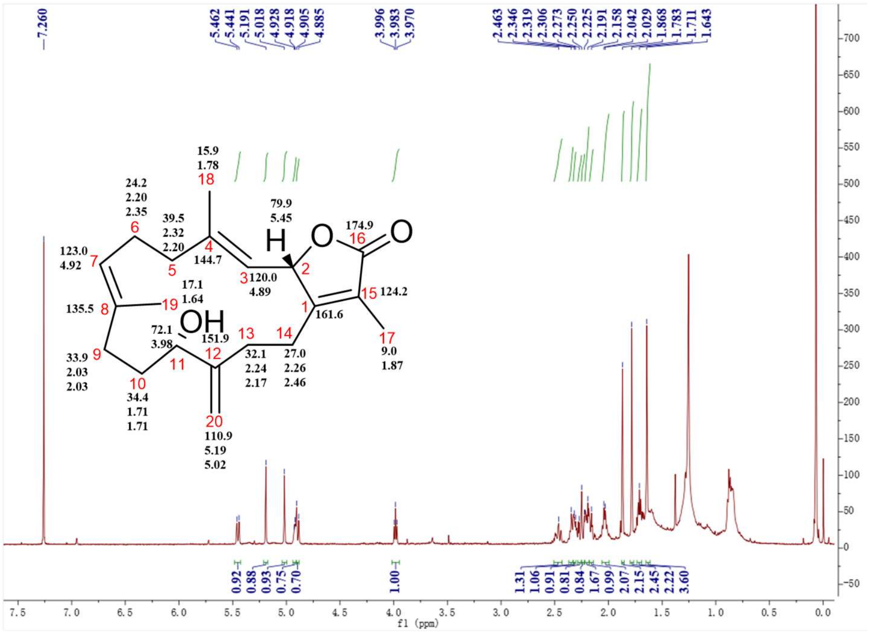

Figure S47. ^{1}H NMR spectrum (500 MHz, $CDCl_3$) of compound **5.**

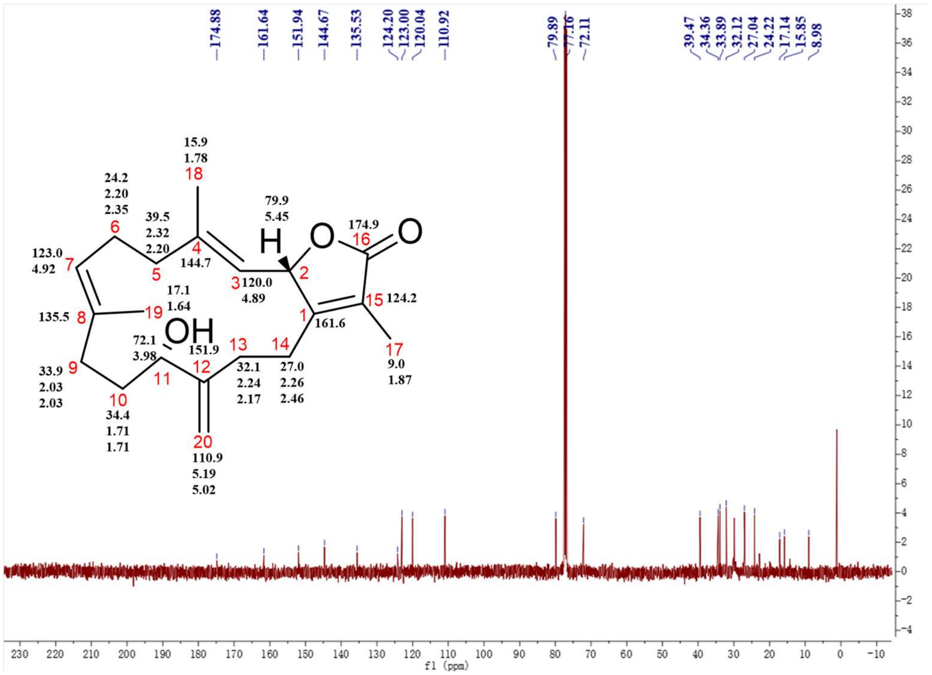

Figure S48. ^{13}C NMR spectrum(125 MHz, $CDCl_3$) of compound **5.**

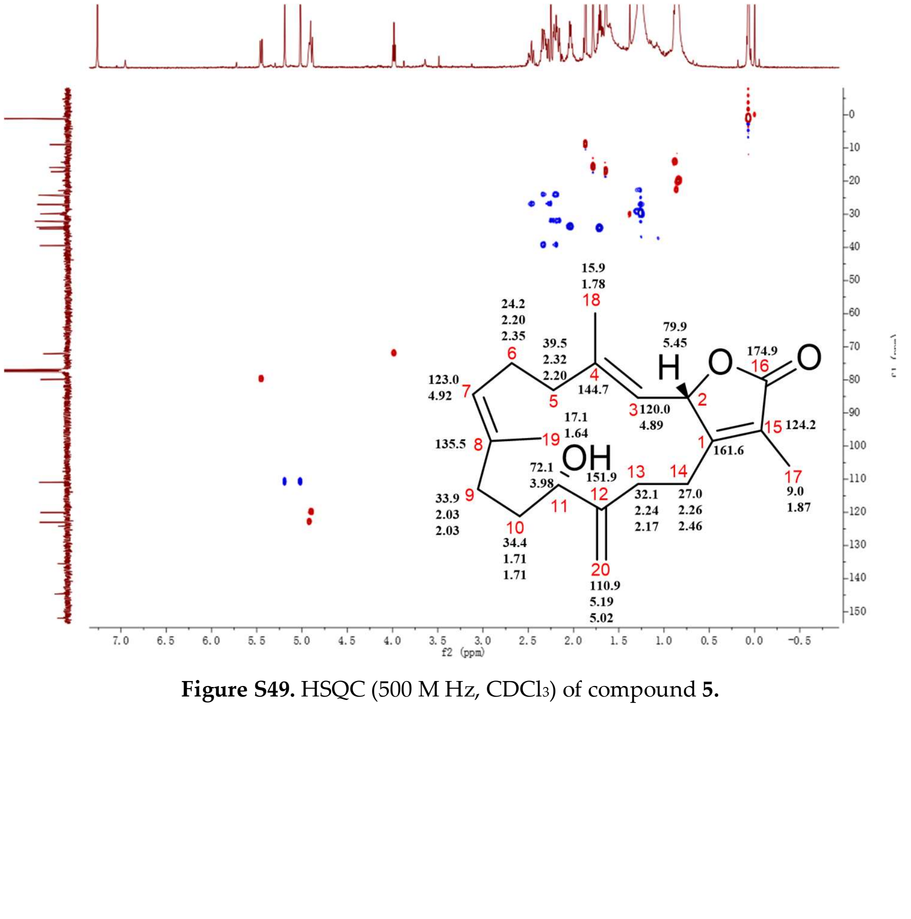

Figure S49. HSQC (500 M Hz, $CDCl_3$) of compound **5.**

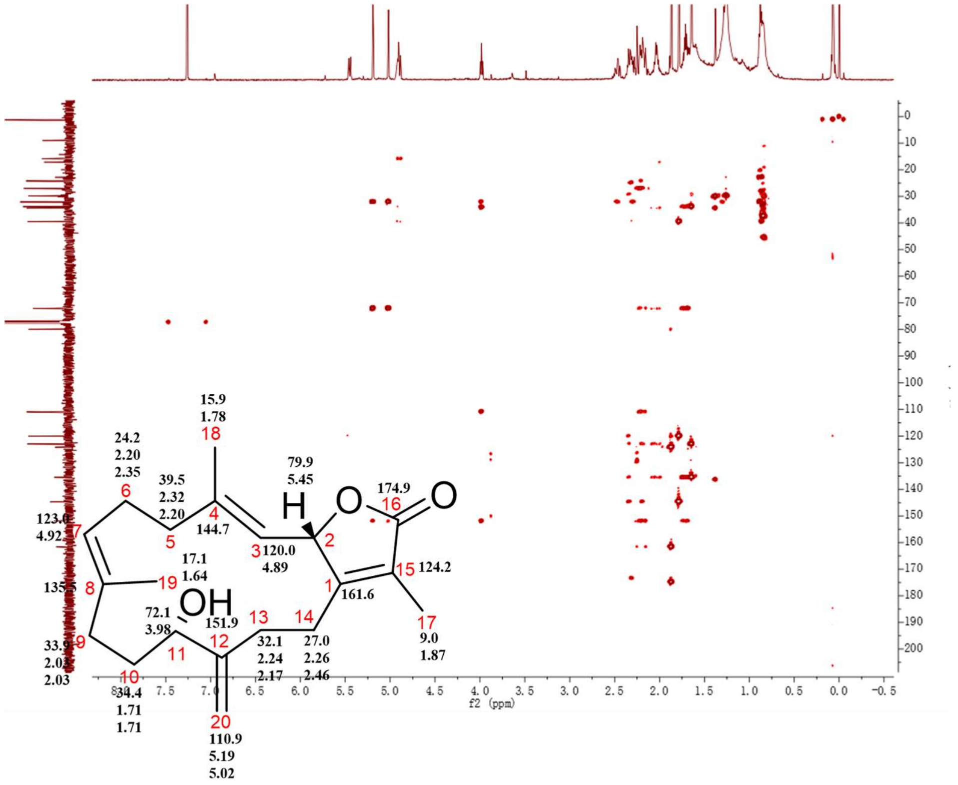

Figure S50. HMBC (125 MHz, $CDCl_3$) of compound **5.**

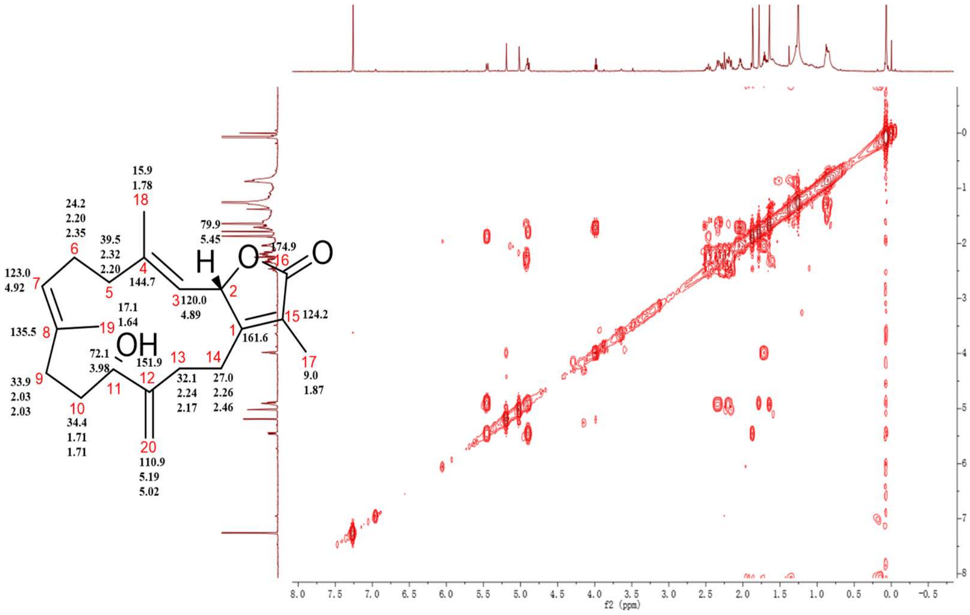

Figure S51. ^1H-^1H COSY (500 MHz, CDCl3) of compound **5.**

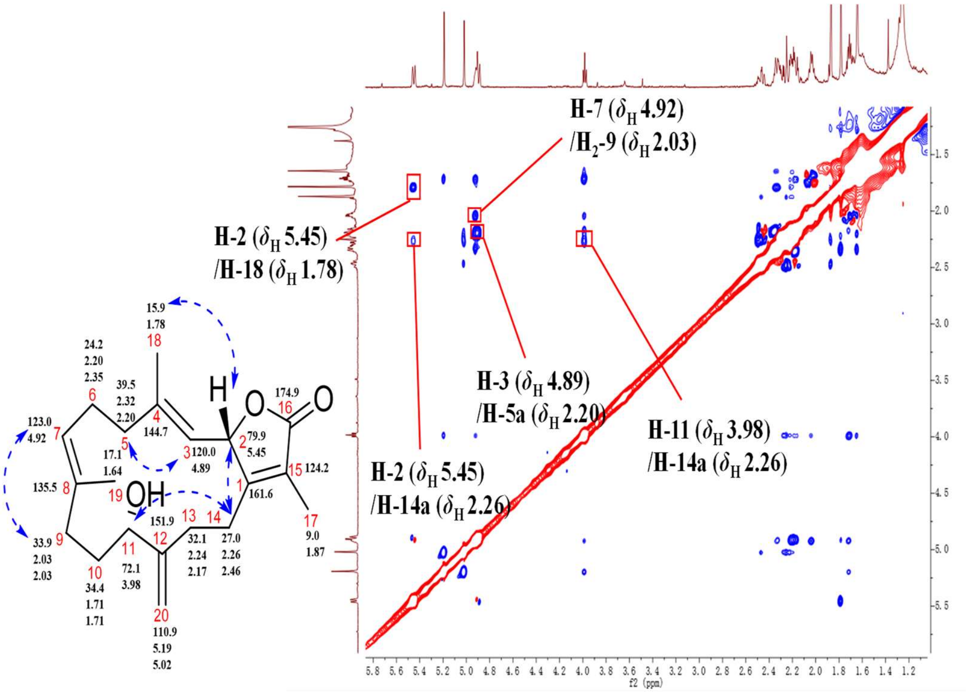

Figure S52. NOESY (500 MHz, CDCl3) of compound **5.**

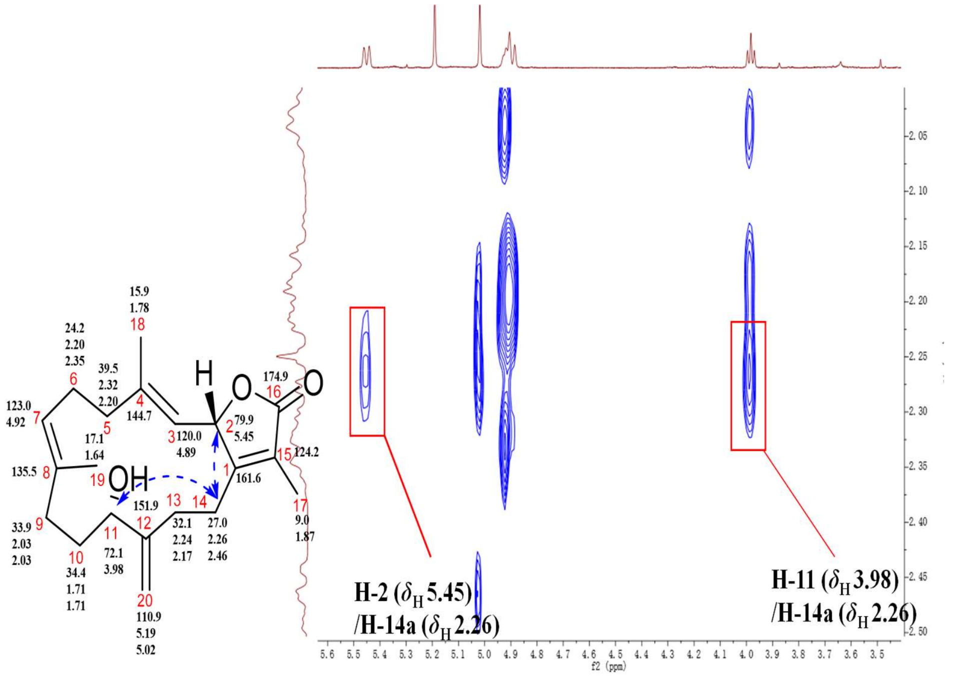

Figure S53. NOESY (500 MHz, CDCl3) of compound **5.**
